# Analysis of official deceased organ donation data casts doubt on the credibility of China’s organ transplant reform

**DOI:** 10.1186/s12910-019-0406-6

**Published:** 2019-11-14

**Authors:** Matthew P. Robertson, Raymond L. Hinde, Jacob Lavee

**Affiliations:** 1 Present Address: School of Politics & International Relations, College of Arts & Social Sciences, Australian National University, Canberra, Australia; 2 China Studies Research Fellow, Victims of Communism Memorial Foundation, Washington, DC USA; 3 Canberra, Australia; 4 Heart Transplantation Unit, Department of Cardiac Surgery, Leviev Heart Center, Sheba Medical Center, Sackler Faculty of Medicine, Tel Aviv University, Tel Aviv, Israel

**Keywords:** Organ transplantation, Transplant ethics, Organ transplantation in China, Organ donation, Statistical forensics, Data falsification

## Abstract

**Background:**

Since 2010 the People’s Republic of China has been engaged in an effort to reform its system of organ transplantation by developing a voluntary organ donation and allocation infrastructure. This has required a shift in the procurement of organs sourced from China’s prison and security apparatus to hospital-based voluntary donors declared dead by neurological and/or circulatory criteria. Chinese officials announced that from January 1, 2015, hospital-based donors would be the sole source of organs. This paper examines the availability, transparency, integrity, and consistency of China’s official transplant data.

**Methods:**

Forensic statistical methods were used to examine key deceased organ donation datasets from 2010 to 2018. Two central-level datasets — published by the China Organ Transplant Response System (COTRS) and the Red Cross Society of China — are tested for evidence of manipulation, including conformance to simple mathematical formulae, arbitrary internal ratios, the presence of anomalous data artefacts, and cross-consistency. Provincial-level data in five regions are tested for coherence, consistency, and plausibility, and individual hospital data in those provinces are examined for consistency with provincial-level data.

**Results:**

COTRS data conforms almost precisely to a mathematical formula (which first appeared to be a general quadratic, but with further confirmatory data was discovered to be a simpler one-parameter quadratic) while Central Red Cross data mirrors it, albeit imperfectly. The analysis of both datasets suggests human-directed data manufacture and manipulation. Contradictory, implausible, or anomalous data artefacts were found in five provincial datasets, suggesting that these data may have been manipulated to enforce conformity with central quotas. A number of the distinctive features of China’s current organ procurement and allocation system are discussed, including apparent misclassification of nonvoluntary donors as voluntary.

**Conclusion:**

A variety of evidence points to what the authors believe can only be plausibly explained by systematic falsification and manipulation of official organ transplant datasets in China. Some apparently nonvoluntary donors also appear to be misclassified as voluntary. This takes place alongside genuine voluntary organ transplant activity, which is often incentivized by large cash payments. These findings are relevant for international interactions with China’s organ transplantation system.

## Background

China’s practices of organ procurement and transplantation have been a matter of international medical and ethical attention for several decades. Since the inception of the field, organ procurement in China has been closely tied to the state judicial system, which provided nonvoluntary organ donors to transplant hospitals. In 2010, in response to increasing attention and criticism, China’s medical administrators began the first of a series of pilot programs that had the stated goal of transitioning the country from the use of prisoners as an organ source to voluntary donors, in accordance with international medical norms.

The new system has a number of key features that differ from the past: a national computerized organ allocation and registration infrastructure, known as the China Organ Transplant Response System (COTRS); clear definitions of death by both circulatory and neurological diagnostic criteria; a network of hospital-based organ procurement organizations (OPOs); transplant coordinators affiliated with both local hospitals and branches of the Red Cross Society of China; and a policy of “humanitarian aid” for families of deceased donors.

Chinese health authorities report that they have been steadily implementing the above policies and regulations since 2010. From September 1, 2013, COTRS was mandated for the allocation of all organs, no matter the source [1]. In December 2014, Chinese officials announced that beginning on January 1, 2015, hospital-based procurement would be the only lawful source of organs in China [2].

Since the pilot program began, China’s healthcare officials have reported extraordinary successes. According to COTRS data, from 2010 to 2018, annual voluntary deceased donors went from 34 to 6316, an increase by 185 times; kidneys and livers transplanted went from 63 in 2010 to 10,481 in 2016 (the last year for which precise data is available), an increase by 166 times.

These reports of success in reform have been welcomed by international medical organizations, including The Transplantation Society (TTS) and the World Health Organization (WHO) [3, 4]. Dr. Huang Jiefu, China’s former vice minister of health and the architect of the organ transplant reforms, was invited to the Pontifical Academy of Sciences (PAS) at the Vatican in February 2017 to present data on China’s successes. While there, he also provided an outline for a China-backed WHO task force that would “eradicate organ trafficking” internationally [3]. The proposal was endorsed by both WHO and TTS officials in a written communication to Huang [4]. Subsequent interactions have further established the international support enjoyed by Dr. Huang’s reforms [5].

Yet despite being at least the second-largest organ transplant country in the world [6], China’s standards for public provision of its transplant data differ significantly from elsewhere. None of the official organ transplant registries are publicly accessible. With rare exceptions, gathering data on organ transplant activities in China requires manual collection from official sources, including state-run publications, official websites, and clinical papers. Official data, typically provided by Dr. Huang Jiefu and his protégé, Dr. Wang Haibo, director of COTRS, is often sparse in detail and difficult to independently corroborate.

China’s claims of no longer using prisoners as an organ source are likewise difficult to corroborate. For decades the only nationally-applicable regulation in the transplant field was the 1984 “Temporary Rules Concerning the Utilization of Corpses or Organs from the Corpses of Executed Criminals,” issued by China’s Supreme People’s Court, Supreme People’s Procuratorate, Ministry of Public Security, Ministry of Justice, Ministry of Health, and Ministry of Civil Affairs. It was and remains the only known quasi-legal basis justifying the use of executed prisoners for their organs [7]. Dr. Huang Jiefu has claimed no knowledge of this document [8].

China’s organ transplant sector underwent a period of rapid growth beginning in 2000, during which time no formal regulations were put in place until July 2006 [9]. Those Interim Regulations, banning the sale of organs and setting standards for organ transplants, were issued by the then-Ministry of Health on March 16, 2006 [9], 1 week after witnesses emerged alleging that practitioners of Falun Gong, a persecuted Chinese spiritual practice, were being used as an organ source, a claim that Chinese officials deny. The Interim Regulations were formalized in the Regulation on Human Organ Transplantation issued by the State Council in May 2007 [10].

Other regulatory and legislative changes since then include an amendment to the Criminal Law in 2011 making organ trafficking illegal, and the National Health and Family Planning Commission’s (NHFPC) provisions on organ donations in August 2013. These documents are the backbone of the current legal and regulatory framework for China’s organ transplantation sector. According to Huang Jiefu: “These three documents prescribe that the source of the organ must be voluntary, unpaid, open, transparent and traceable. That is, knowing where the organ is from and where the organ is to go.” [8] In the context of China’s voluntary organ donor reform, Chinese officials have said that both the deceased and their family must affirmatively consent to the donatio n[11].

The use of prisoners as an organ source has been a major source of controversy for China’s organ transplantation system. The negative international attention it brought has been a key impetus behind the reform efforts [12]. The path to the final declaration that prisoners would no longer be used, however, has been complicated.

For many years, Chinese officials denied that prisoners were used as an organ source. In 2001, after a Chinese surgeon defector told U.S. Congress that he was involved in the removal of skin from executed prisoners, Zhang Qiyue, the foreign ministry spokeswoman, called his testimony “sensational lies” and “vicious slander” [13]. Zhang said: “With regard to the trade in human organs, China strictly prohibits that. The major source of human organs comes from voluntary donations from Chinese citizens” [13].

Despite performing around 120,000 organ transplants from 1977 to 2009, according to Allison et al., Chinese officials admitted to only 130 being voluntary by end 2009 [14].

In late 2005, Chinese officials first publicly admitted to the use of organs from executed prisoners [15]. Human rights organizations, independent researchers, and legislative bodies have subsequently presented evidence alleging that detained practitioners of Falun Gong, Uyghurs, and other prisoners of conscience have been used on a wide scale as an organ source [16–20]. Chinese officials deny this.

After several years of defending the use of death row prisoner organs, the first promise that the practice would end took place on November 2, 2013, during an organ transplant conference in Hangzhou. There, leaders of China’s 38 largest transplant centers “voluntarily made a written commitment to the cessation of organs from executed prisoners.” The National Health and Family Planning Commission (NHFPC) also “expressed the resolution of the government of China that the dependence upon organs from executed prisoners must be terminated” [21].

The practice continued, however, to the frustration of international transplantation authorities. The leadership of The Transplantation Society and the Declaration of Istanbul Custodian Group (DICG) penned an open letter to China’s president Xi Jinping in March 2014 stating that China is “scorned by the international community for this practice” [22].

The next promise that prisoners would no longer be used for organs was made by Huang Jiefu on December 3, 2014, at China’s national organ transplantation conference in Kunming, Yunnan Province [23]. But both before and after making this vow, Huang told Chinese media that prisoners may in fact still be used.

On January 11, 2015, he told a state-linked broadcaster: “I’m not saying I oppose death-row prisoner donations. If the death-row prisoners are truly moved by their conscience, then it’s not impossible. But it must go through the citizen organ donation system, through the Red Cross, through the online computer system for a fair and equitable distribution. That’s transparent.” [24] On January 28, 2015, he told People’s Daily: “Death-row prisoners are also citizens. The law does not deprive them of the right to donate organs. If death-row prisoners are willing to atone for their crime by donating organs, they should be encouraged.” [25]

The negative attention these remarks received, once translated into English and publicized, led Huang to backtrack on the statements. In November 2015 he told The New York Times that he had meant the multiple comments only “philosophically,” and that “on a practical level, we cannot do that, to put them into the civilian donation” system [26].

These reassurances appear to have mollified international medical organizations. In August 2016 Huang was invited as a representative of China to provide a special lecture to all attendees at The Transplantation Society’s biennial conference in Hong Kong [27]; in February 2017 he was invited to the PAS meeting at the Vatican where he proposed support for a WHO organ trafficking task force.

In July 2017, according to Chinese media reports, WHO, PAS, TTS, and DICG issued a joint letter to Huang Jiefu saying that China’s organ transplantation system was “ethically proper,” and called for “more engagement from the country to global governance in the sector.” [28] Representatives from the four organizations attended China’s transplant congress in Yunnan in August 2017, further affirming the reform program in media interviews.

China’s transplant administrators have also been supported by the Rockefeller Foundation-endowed China Medical Board, which has provided grants of nearly $2 million for reforms to China’s transplant system since 2006, including for the construction of COTRS, on the basis that it would become an accurate allocation system free of prisoner organs [29, 30].

These international medical organizations have in general based their change of policy towards China upon pre-arranged visits to only few transplant centers, and have not questioned how China managed to achieve such extraordinary rates of growth in hospital-based organ procurement, nor sought to verify the claims made by Chinese officials about the cessation of use of nonvoluntary donors [31].

There currently exists no independent, scientific examination of China’s ambitious program of organ transplant reform, a gap that this study seeks to fill.

This work is based upon data gathered from the limited revelations made by Chinese officials and from authoritative sources available online. These data are submitted to a battery of mathematical tests to examine their internal consistency (that the data in a single series is consistent), congruence between one another (that two datasets that ought to correspond to one another, in fact do so), and integrity (that the data is within the realm of medical possibility and plausibility).

The chief aim of this study is to ascertain whether the pronouncements by Chinese officials claiming enormous successes in transplant reform are based upon sound and credible accretive data created by the real-time organ donation and transplantation activity taking place in hospitals across the country, or are instead based upon artificially manufactured data at both central and provincial levels.

## Methods

The study design involves an examination of the known and available datasets associated with China’s voluntary organ donation system: COTRS, Central Red Cross, and provincial Red Cross data. The COTRS and Central Red Cross datasets were individually examined for internal integrity and consistency, and then compared to one another for congruence. Finally, five provinces were sampled: provincial Red Cross data in those provinces were examined for internal integrity, and then compared to reported transplant activity at hospitals in the respective province for congruence.

### China organ transplant response system data

#### About the data

COTRS is the basis of China’s current voluntary organ donation reforms. It is a mandated organ allocation system, meaning that every organ transplant must be allocated solely through COTRS.

China’s top sixteen transplant officials make this clear in an August 2016 paper:

*The China Organ Transplant Response System (COTRS,* *http://www.cot.org.cn* *) is the sole legitimate official organ allocation computer system in China. Starting from September 1, 2013, all community-based donated deceased organs are mandated to be allocated through COTRS. Out of COTRS allocation is forbidden and subjected to the revoke of license of organ transplantation and possible incrimination according to the Amendment VIII of Criminal Law. Thus, COTRS documented every legitimate organ donation and allocation since then* *[32]* *.*

#### Data collection

Though this data is not accessible via the COTRS website, it has been made public on two occasions, and the deceased donor portion of it updated subsequently.

The first known publication of part of this dataset (hereafter “COTRS 2014”) was on August 18, 2014, in the pages of The Beijing News, a major newspaper under the administration of the Beijing municipal propaganda department. The article cited Huang Jiefu as the source of the data, and contained an interview with him. It featured cumulative annual figures for voluntary deceased donors and donated organs from 2010 to August 14, 2014 [32].

A fuller dataset, from 2010 to 2016, was made public during a presentation by Huang at an anti-organ trafficking meeting at the Vatican’s Pontifical Academy of Sciences in February 2017 (“COTRS 2017”) [33, 34]. Huang presented the data to over 200 international organ transplantation experts, with a live feed to media in an antechamber [35]. In this public presentation, only figures for donors and transplanted livers and kidneys (both living and deceased), rather than all organs, was provided.

After the preparation and submission of an initial version of this manuscript in April 2018, a portion of this dataset was updated again. On July 2, 2018, at The Transplantation Society’s biennial conference held in Madrid, Dr. Wang Haibo, the head of COTRS, presented new data from the COTRS database (“COTRS 2018”) in a presentation titled “Achieving organ donor reform in China.” [36]

In his presentation, Dr. Wang stated that there were 5146 voluntary deceased donors in China in the year 2017. This datapoint has subsequently appeared in authoritative Chinese media, including Xinhua, the official news agency of the People’s Republic of China, further clarifying that it is the authoritative, official figure [37]. Dr. Wang did not provide updates of the precise number of deceased kidney and liver donations as in the COTRS 2017 series.

### Central Red Cross data

#### About the data

The Red Cross Society of China manages the China Organ Donation Administrative Center, which during the majority of the period of our data collection published voluntary organ transplant data on its website www.china-organdonation.org.cn. This data was mirrored on www.china-organdonation.org, www.rcsccod.org.cn and www.rcsccod.org, which are also run by the Red Cross. Subsequent to completing an initial draft of this paper, several of these websites were taken offline; currently the website www.codac.org.cn remains online and continues the same data series.

The data is updated at nonuniform intervals ranging from days to months. In theory the sources of the data come from Red Cross office branches in provinces across the country, since it is these local Red Cross staff who register the donations in the central database, as well as report them locally.

The first known update to the central database was on March 31, 2014. Each update provides four items of data: the date at which the update took place, the cumulative total of voluntary deceased donors, the cumulative total of organs transplanted, and the current total of registered volunteer donors. Registered volunteers, also known as designated donors, refer to living individuals who have expressed a willingness to donate after death.

When this data is updated, a new set of figures appears on the websites above. None of the Red Cross websites provide the historical series.

This dataset is termed “Central Red Cross” to make clear its institutional affiliation and to distinguish it from provincial Red Cross data.

The Red Cross’s website notes that its figures come from organ procurement organizations (OPOs) across the country, which allocate the organs through COTRS and then register them in the Red Cross database. Every transplant is logged in both COTRS and Red Cross [38].

There is one exception to the method of Red Cross’s provision of data. Subsequent to COTRS 2018, further data — of 6316 deceased donors in the year 2018 (“Red Cross 2019”) — was published in the People’s Daily in April 2019 and attributed to the Red Cross Society of China with no mention of COTRS [39]. (A figure of 6300 donors for year 2018 was presented as “Predicted” on a slide by Dr. Wang during his July 2, 2018 TTS session, based on data ending in April 2018 [40]. As of May 2019, COTRS’ website, www.cotdf.org, was inaccessible.)

#### Data collection and processing

The Red Cross website and its mirrors were accessed on a regular basis from April 2014 to the end of October 2017. Each time the data was updated, the page was saved in an archive using the online Internet Archive service at www.archive.org. Redundant backups of these archives were made at www.archive.is. When pages were unable to be archived for technical reasons, screenshots were made and uploaded to the Internet Archive.

The sets of four items of data were logged in a table, creating a first of its kind historical series of Central Red Cross data, which enabled analysis of the dataset. (Additional file 2).

#### Data analysis

The following methods of analysis were applied to both the COTRS (2017 and 2018) and Central Red Cross datasets.

Each dataset was first assessed for anomalies of internal consistency and integrity. This included searching for:

- Unambiguously contradictory data — for example, mismatches in what are supposed to be identical data series or decreases in cumulative series;
- Anomalies that undermine the integrity of the data — for example, implausibly high ratios of transplants per donor.

Secondly, each dataset was examined for features that would distinguish it as having been generated via an artificial process indicative of human manipulation. This involved searching for mathematical patterns or anomalies that are inconsistent with unpredictable transplant activity taking place across a large country. Tests included:

- Extremely close adherence of trends to simple mathematical formulae or patterns;
- Persistent adherence to arbitrary internal ratios between data series;
- The occurrence of apparent non-randomness in specific numbers — for example, data series increments that are a precise doubling, or are a precise number of thousands.

In the case of series that appeared to conform too closely to a simple mathematical function, their closeness of fit statistics (R-squared) were analyzed to see if they were distinctly unusual. Due to the complex and random data pattern expected to be exhibited by a growing and geographically-distributed voluntary organ allocation and transplantation system, it is unsuitable to use formal measures of statistical significance for establishing whether data is *too close* to a given model of behavior. Instead, the close adherence of Chinese data to the model was compared to that of other countries, to assess abnormality. This involved use of comparable data for 50 other countries found in the Global Observatory on Donation and Transplantation (GODT) database available at www.transplant-observatory.org. This analysis, which examines the R-squared and mean squared errors, is presented in Additional file 1.

COTRS 2018 data, which became available after the completion of the initial draft of this manuscript, was also used as a test case for the persistence of adherence to the mathematical formulae found in COTRS 2017.

#### Comparison between COTRS and Central Red Cross data

Central Red Cross data was compared to COTRS data to assess whether or not it was identical and/or congruent. Tests of identicality and congruence included:

- Whether the data featured the same number of donors on the same dates;
- Whether the gap between the number of organs transplanted in the COTRS dataset (which for some years includes only livers and kidneys) and the Red Cross dataset (all solid organs) was able to be accounted for via the number of other known organ transplants (primarily heart and lungs) during the periods in question.

### Local Red Cross data

#### About the data

The Red Cross Society of China has branches across the country at the provincial-, and sometimes county- and municipal-levels. Red Cross employees are supposed to participate in every transplant by acting as a third-party witness and in some cases transplant coordinators. This is separate from the hospital performing the operation.

Huang Jiefu stated in April 2017: “If there was no Red Cross participation, there would be no organ donations in China.” [41]

With the exception of four regions — Beijing, Qinghai, Ningxia, and Tibet — all Red Cross branches report the number of voluntary donors and transplants performed in that region either on local Red Cross websites or in local authoritative (typically state- or Communist Party-affiliated) media during the period of data collection. In the absence of access to the Red Cross database which contains each provincial data series, incremental reports on provincial Red Cross websites and in the local state-run press are the only channels through which the information is available. These updates are taken as authoritative, official figures by actors within the Chinese system.

#### Data collection and processing

Local Red Cross data for 28 (out of a total of 31) provincial level administrative units (including regions and directly-administered municipalities) was collected via searches in Google and Baidu, China’s largest search engine, using keyword strings such as: “*province name + organ donation + case*” or “*province name + Red Cross + organ donation.*”

Reference was made to data presented directly on local Red Cross websites or in authoritative (usually state-affiliated) media who cite Red Cross personnel. The provincial Red Cross dataset was submitted to the same tests for integrity and internal consistency as the Central Red Cross and COTRS data.

#### Comparison between local Red Cross and hospital-level data

In order to examine whether hospital activity in provinces supported the reported Red Cross figures in those provinces, five provinces were selected for forensic data analysis. The provinces were selected based on a number of heuristics for the detection of questionable data, including: precise doubling of numbers, implausible transplants-to-donor ratios, or reports of a large number of transplants in regions with relatively less developed healthcare infrastructure.

The websites of each of the transplant hospitals in these provinces were examined, with this data compared to the provincial Red Cross data.

There is no official and transparent dataset for the transplant activity that takes place at any particular hospital. Thus, the absence of data on hospital websites or in clinical papers, while suggesting the absence of transplants, does not rule out unpublicized yet legitimate transplants.

The Results of this analysis may be found in Additional file 5.

## Results

Following are the results of the analysis of the three datasets according to the methods outlined, followed by comparisons between them.

### COTRS 2017 data

Data from COTRS presented by Huang Jiefu publicly at the Vatican in February 2017 is shown in Table 1.

**Table 1:** COTRS Central-level Annual Deceased Donor and Transplant Figures. Presented by Huang Jiefu in February 2017

Year	Deceased donors	Deceased kidney transplants	Deceased liver transplants
2010	34	33	30
2011	132	166	94
2012	433	743	318
2013	849	1533	629
2014	1702	2986	1301
2015	2766	4931	2150
2016	4080	7224	3257

**Fig. 1:**
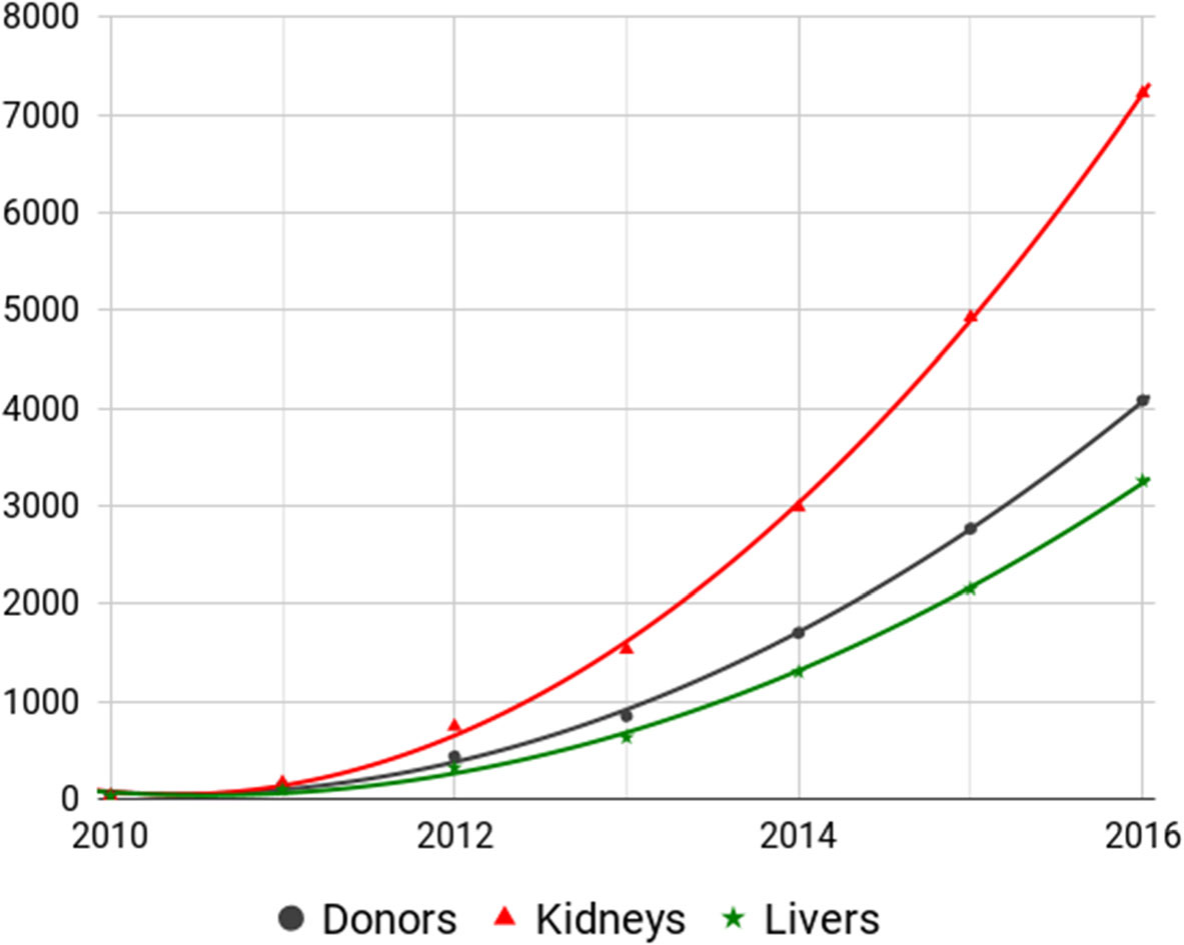
COTRS Central Level Annual Figures Presented by Huang Jiefu in February 2017. The line of best fit, added, is a quadratic formula with a growth curve that takes the form *y = ax*^*2*^ *+ bx + c.* The coefficients for the three lines and a full mathematical explanation by which the line of best fit was derived can be found in Additional file 1

The procedure of data analysis described in the Methods reveals that the data exhibits an extremely close adherence to a simple mathematical function, specifically a quadratic equation. There is no particular reason that the growth rate of a large, complex and geographically scattered industry would adhere so closely to *any* simple mathematical function (i.e. a mathematical function with few parameters). This is shown in Fig. 1.

The mathematical identity known as the R-squared statistic, a widely known measure of closeness of fit, was used to measure how close the COTRS data is to the fitted quadratic functions. An R-squared of 1 is a perfect fit, meaning that there is no difference between the data and the line to which it is being fitted. If the R-squared statistic in this case were 1, it would mean that not only was the underlying growth rate quadratic, but also that there was no random variation due to any other background cause.

The R-squared statistics for these data are 0.9993, 0.9995 and 0.9989 for donors, kidneys and livers respectively. This is extremely close to the fitted quadratic functions.

The R-squared statistic can be difficult to interpret correctly. It is problematic to apply a formal statistical significance test to this data to determine whether it is *too close* to a quadratic curve, primarily because the behavior of structural growth in an industry, especially one involving as many contingencies as voluntary organ transplantation, is difficult to quantify. For this reason the same measures of closeness of fit were calculated for comparable data for 50 other countries from the Global Observatory on Donation and Transplantation (GODT) database.

It was found that when fitted to quadratic equations, every other country was between one and two orders of magnitude further away from the perfect R-squared of 1 compared to China. Of all other countries, the closest R-squared was 1.30% away from a perfect 1, with others ranging down to 99.9% away from 1; China’s three values ranged between .112% to .0478% away from 1. It was further discovered that the mean squared errors of China did not conform to the pattern exhibited by all other countries. Additional file 1 contains the technical details of this analysis and further discussion on the issue of statistical significance.

### COTRS 2018 data

Data from COTRS presented by Wang Haibo publicly at TTS 2018 in Madrid in July 2018 is shown in Table 2. Analysis of this data allows for a simplification of the quadratic model discovered in the COTRS 2017 data, wherein the underlying data appears to be based on a simpler one-parameter quadratic formula.

**Table 2:** COTRS Central-level Annual Deceased Donor Figures With X Values. Presented by Wang Haibo in July 2018

Year	X value	Deceased donors
2010	0	34
2011	1	132
2012	2	433
2013	3	849
2014	4	1702
2015	5	2766
2016	6	4080
2017	7	5146

This extended data was also fitted to a quadratic equation. The following table shows the quadratic line of best fit without, and then with, the 2017 data point (Table 3). (Note that the parameters a, b and c for the 2010–2016 data are different from those given in Additional file 1. This is because the previous analysis used 2009 as year zero while now 2010 is used.)

**Table 3:** Results of Fitting y = a.x^2^ + b.x + c to Annual Deceased Donors From COTRS 2018 Data

Parameter	Data from 2010 to 2016	Data from 2010 to 2017
a	128.2	107.9
b	− 102.3	−.5
c	68.1	7.1

Parameters b (−.5) and c (7.1) have become very small, meaning that they contribute very little to the goodness of fit, so that fitting y = a.x^2^ gives almost as good a result as fitting y = a.x^2^ + b.x + c. The table below shows the results of fitting y = a.x^2^ compared to y = a.x^2^ + b.x + c(Table 4).

**Table 4:** COTRS 2018 Data Fitted From 2010 to 2017 With R-Squared Values

Parameter	y = a.x^2^ + b.x + c	y = a.x^2^
a	107.9	108.0
b	−.5	
c	7.1	
r^2^	.99692	.99691

It is again evident that there is virtually no difference in the parameter ‘a’ nor in the r^2^ values.

A significant remaining question is whether the value of 5146 for 2017 is in some sense significant or special. This was tested by looking at scenarios where the 2017 value ranged from 4950 to 5350. For each value, the two models (y = a.x^2^ + b.x + c and y = a.x^2^) were both fitted, and the reduction in SSE (sum of squares of errors from the model) obtained by using the former instead of the latter was calculated.

**Fig. 2:**
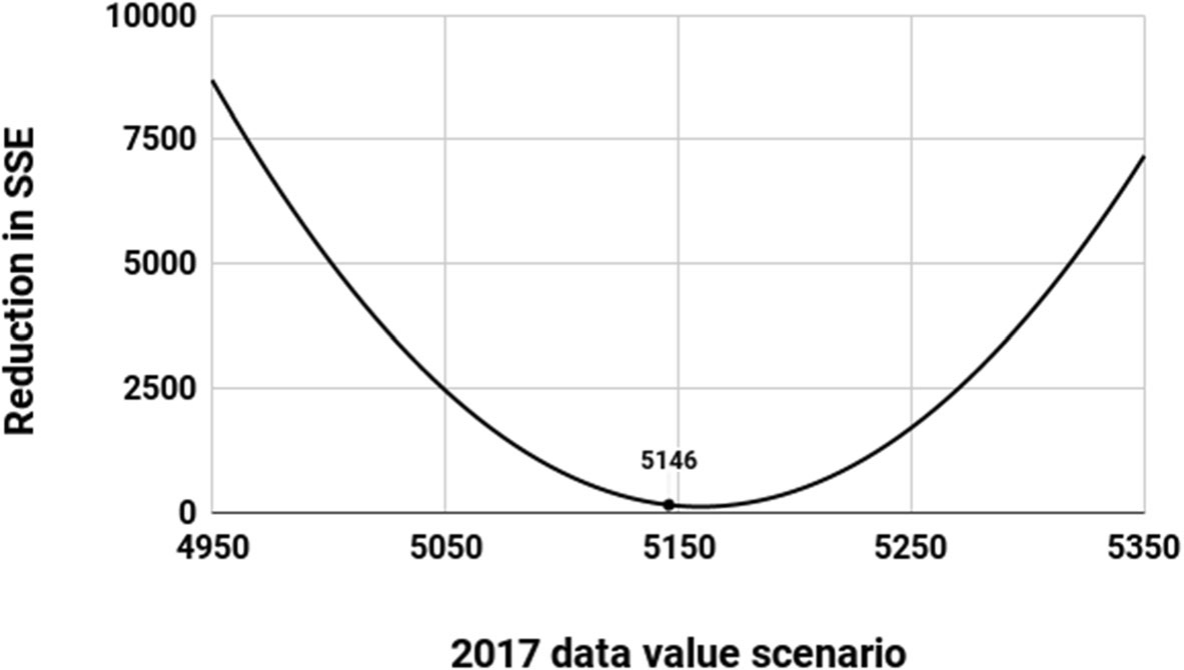
Reduction in SSE by Using y = a.x^2^+ b.x + c Instead of y = a.x^2^. The figure plots a range of values that the 2017 annual deceased donor figure may have taken in the COTRS 2018 dataset, showing that the actual value (5146) was extremely close to the value that would have most supported simplification of the model

The resulting graph gives a visual indication of the significance of the datapoint 5146. It can be seen that 5146 lies very close to the minimum contribution from b and c which occurs at 5160. In other words it is almost exactly the 2017 value that would *most* strongly reinforce the model simplification to y = a.x^2^(Fig. 2).

The reduction of the model to y = a.x^2^ allows for further analysis of the power function. While there is no reason real world data would conform extremely closely to any power function y = a.x^q^ for any value of q, if it did there is no reason why the power q should be an integer such as q = 2 giving y = a.x^2^.

**Fig. 3:**
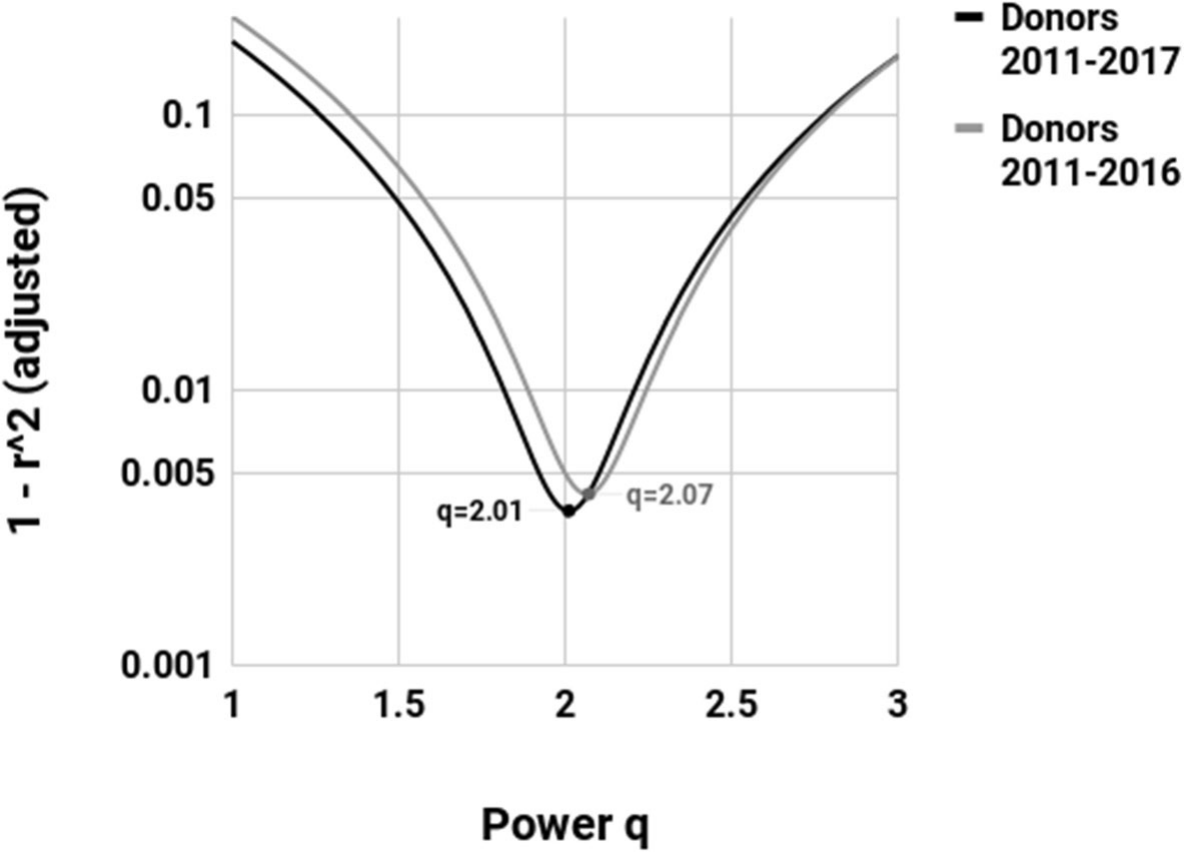
Sensitivity to q When Fitting the Model y = a.x^q^. The figure shows a range of values for q in the COTRS 2017 and COTRS 2018 series for deceased donors, showing that the addition of the 2017 value of 5146 moves q, for the optimal y = ax^q^, much closer to 2 (from 2.07 to 2.01) and further reduces 1-r^2^(adjusted)

Figure 3 compares fitting q = 2 to fitting other values of q. This was done by fitting a.x^q^ to the data, for q ranging from 1 to 3, and calculating the (adjusted) r^2^ for each q.

By adding the 2017 datapoint, the optimal q shifts from y = a.x^2.07^ (the lowest point of the grey line) to y = a.x^2.01^ (the black line). The optimal power model is now almost identical to y = a.x^2^. (In Fig. 3, the power models do not include the datapoint for the year 2010. This is because the model y = a.x^q^ is not influenced by the value at x = 0 and including it would only have the effect of distorting the comparisons of the r^2^ values).

### Central Red Cross data

The data on which this analysis is based is found in Additional file 2.

**Fig. 4:**
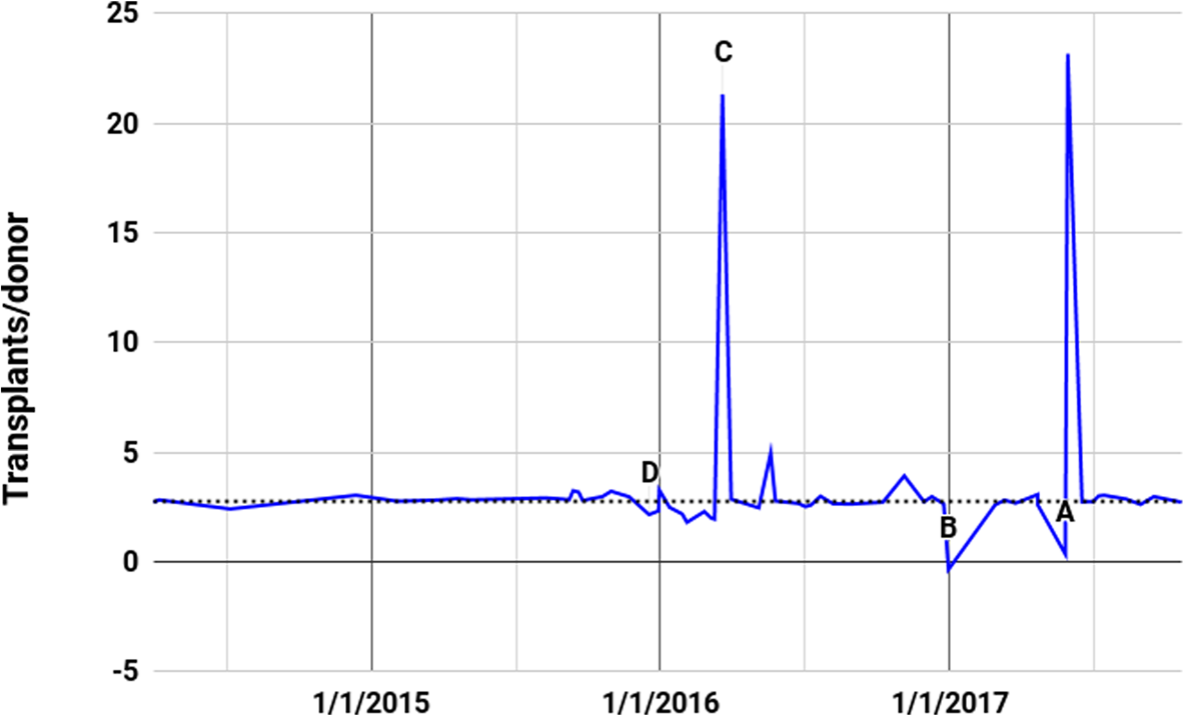
Organs Transplanted per Donor During Each Intervening Period in the Central Red Cross Series Since 4/7/2014. Points on graph are values during the prior time interval. The horizontal dotted line indicates transplants/donor equal to 2.75

Five anomalies were identified in this data, which will be marked as A (including A1 and A2), B, C, and D in the following three graphs. A1 and A2 are grouped together, because it appears that they were caused by the same set of data entry or manipulation problems, and occur consecutively in the series (Fig. 4).

### Anomaly A1: 4/23/2017–4/30/2017

**Fig. 5:**
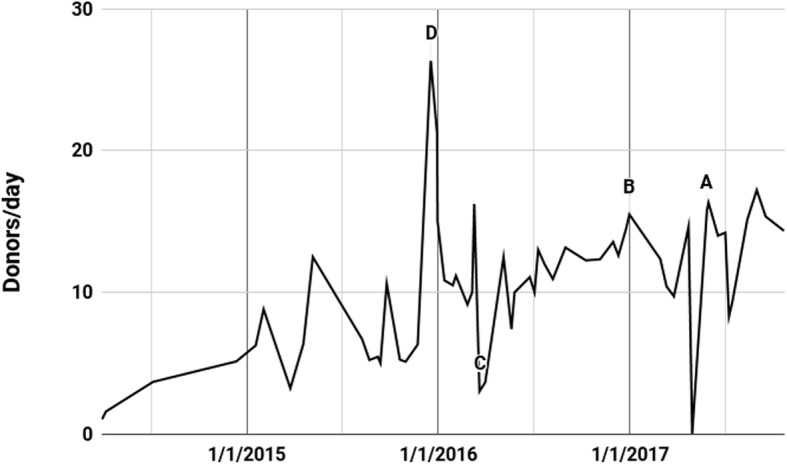
Central Red Cross Data Donors Per Day During Intervals Since 3/31/2014. Points on graph are values during the prior time interval

Anomaly A1 can be seen in Fig. 5 where the donors/day is zero. The Red Cross website china-organdonation.org (since taken offline) updated its website on April 23, 2017 and again on April 30 — though the data remained the same at 11486 donors, 31,703 transplants, and 256,570 volunteers. One of the Red Cross’s mirror websites www.codac.org.cn, however, did update its data on April 23, showing an inconsistent 11,396 donors, 31,470 transplants, and 252,999 volunteers.

### Anomaly A2: 5/28/2017

Anomaly A2 can be seen in Fig. 4 where the transplants/donor rate is 0.33 in the interval ending 5/28/2017 and 23.16 in the following interval ending 5/31/2017. These are both clearly erroneous. Because they are incorrect in the opposite direction it suggests a single transcription error in a cumulative total that affects the apparent rates in the intervals immediately before and after the error. If the cumulative total of organs transplanted of 31,849 at 5/28/2017 were adjusted by one digit to 32,849, the two parts of the anomaly vanish. It is therefore reasonable to accept that this could be an transcription error.

### Anomaly B: 12/31/2016

At 12/31/2016 the cumulative total of transplants drops to 27,613 from the prior total of 27,647 at 12/25/2016. Obviously, cumulative figures cannot decrease. While this can also be explained by a single transcription error, it may not have been; its suspicious circumstances are discussed below.

### Anomaly C: 3/20/2016

Anomaly C is depicted in Fig. 4 on 3/20/2016, where there is a clearly impossible transplants/donor rate of over 21. Yet unlike Anomaly A2 and possibly Anomaly B, it cannot be explained as a transcription error. This is because there is no single entry that could be changed that would make the data series plausible, nor any subsequent or prior balancing error that would account for it.

Table 5 shows the anomaly in the context of the data before and after.

**Table 5:** Central Red Cross Data: Anomaly C

Website Data			Incremental Values			
Date	Cumulative donors	Cumulative transplants	Days	Donors	Organs transplanted	Transplants/donor
3/6/2016	6529	17,464	–	–	–	–
3/10/2016	6594	17,590	4	65	126	1.94
3/20/2016	6624	18,230	10	30	640	**21.33**
3/31/2016	6664	18,344	11	40	114	2.85

Transplants/donor between 3/10/2016 and 3/20/2016 exhibit an impossible rate of 21.33, as highlighted in bold

Even if one were to combine the increments between 3/6/2016 and 3/31/2016, thus averaging the rates over nearly a month, the result would still be an anomalous, and almost medically impossible, 6.5 transplants/donor (derived via (126 + 640 + 114)/(65 + 30 + 40)).

After the 21.33 transplants/donor of 3/20/2016, the *cumulative* series continues as though there was no anomaly. Each entry after the anomaly reinforces the anomaly as a part of the dataset; returning coherence to the dataset would require changing a series of entries around the anomaly.

### Anomaly D: 12/18/2015

Figure 5 represents the implied average number of donors per day during all the intervals between updates appearing on the Red Cross website. Anomaly D is better viewed in Fig. 5 as it involves anomalous behavior in the rates of donors per day.

There is a sudden increase in late 2015 of *interval* donors per day to 26.36 for nearly 1 month, followed by very high rates until 12/31/2015. This anomaly is then followed by a decline in *interval* donor rates to a low of less than 4 per day during most of March 2016.

Another explanation for anomalies C and D could be that a backlog of data was entered, and that for anomaly C the backlog quantities of donors and transplants would be mismatching. This however would contradict the collective statement of China’s top transplant officials quoted above that “…*all community-based donated deceased organs are mandated to be allocated through COTRS. Out of COTRS allocation is forbidden …*” [42].

#### Anomalies linked to a persistent cumulative transplants per donor rate

**Fig. 6:**
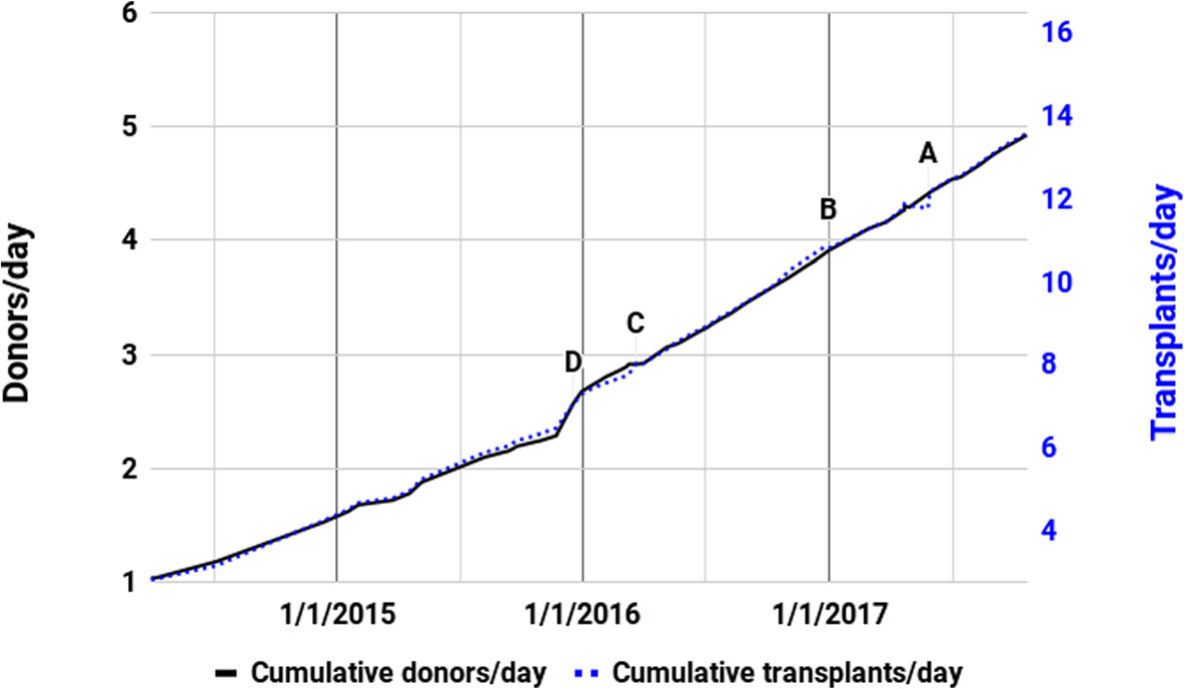
Central Red Cross Data Cumulative Rates Per Day Since 4/7/2014. Scales set so that lines coincide when cumulative transplants/donor equals 2.75

Figure 6 is a composite of the number of donors per day on the left axis and number of organ transplants per day on the right — scaled so that when donors provide 2.75 transplanted organs on average since 1/1/2010, the lines coincide.

Figure 6 shows that the cumulative rate of organs transplanted per donor has generally tracked closely to 2.75. However, in the periods immediately prior to anomalies B, C and D, a gap can be seen emerging for a few months. An update in the data then reverts the cumulative ratio to almost exactly 2.75, bringing the lines back together. These ratios can be examined in more detail in Additional file 2.

In the case of Anomaly D, the *cumulative* transplants/donor rate consistently hovered at over 2.8 for months prior to the anomaly. Its return to 2.75 was achieved partly by a marked lowering of the *interval* transplants/donor rate at D from 2.96 to 2.15 (see Fig. 4). This was combined with a simultaneous extreme increase in the number of *interval* donors per day from 6.30 to 26.36 (see Fig. 5) which had the effect of influencing the *cumulative* transplants/donor rate to a much greater degree. Together they brought the cumulative ratio back from 2.83 to 2.75 in a 25-day interval.

Anomaly C seems to serve a similar function of bringing the rate of organs transplanted per donor from 2.67 abruptly back to 2.75, this time achieved by a 10-day interval with transplants/donor exceeding 21.

Anomaly B did the same, achieved by a 6-day interval with 93 donors and *minus* 34 organs. This brought the *cumulative* rate of transplants/donor from 2.79 to 2.76 in 6 days, followed by 2.75 in the next interval.

Thus, anomalies B, C and D all exhibit the same pattern: over an extended period, the cumulative transplants/donor rate drifts away from 2.75, followed by sudden and medically implausible or impossible data that brings the rate back to 2.75.

### Unusual growth in registered volunteer donors

**Fig. 7:**
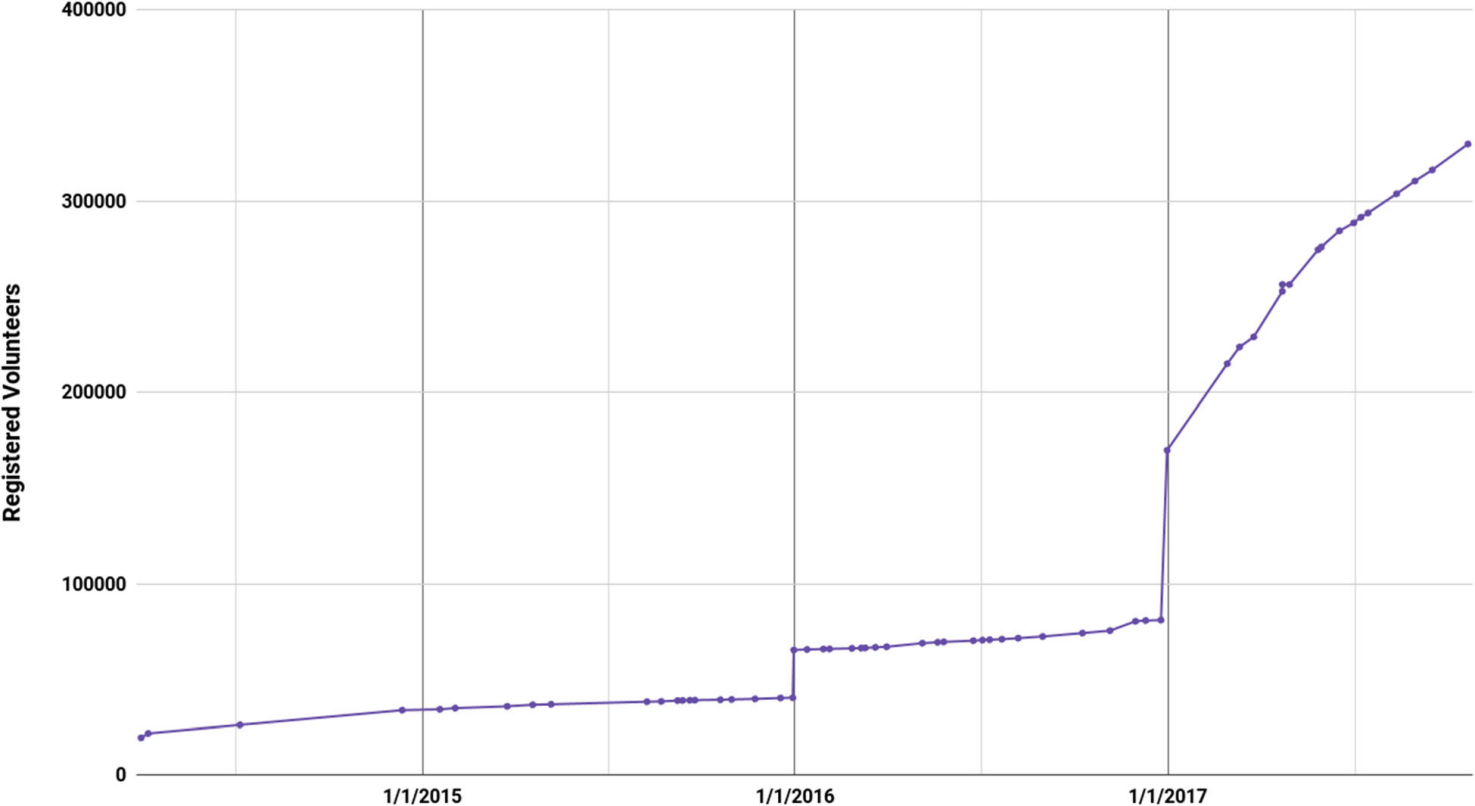
Cumulative Total of Central Red Cross Registered Volunteers

Red Cross data also includes the number of Chinese citizens who are said to have signed a statement to the effect that they are willing to donate organs upon death, assuming their manner of death makes them eligible to do so. This data, as presented by the Central Red Cross, is depicted in Fig. 7.

There are two points at which the number of registered volunteers increased significantly, at the end of 2015 and 2016. The first increase, of exactly 25,000, took place in 1 day, from 12/30/2015 to 12/31/2015, going from 40,322 to 65,322.

Growth in volunteers was modest and gradual through 2016, before doubling at the end of the year with an increase of 88,830 in a 6-day period. Subsequently through 2017 the total consistently increased at a rate far in excess of rates during previous years.

#### Red Cross 2019 data

Subsequent to the incorporation of COTRS 2018 into our analysis, Red Cross officials released the 2018 total for deceased donors, which had the value of 6316. This does not represent the same level of confirmation that y = a.x^2^ is a more powerful predictor as COTRS 2018 does, but is consistent with the simplified model. While the parameters b and c become much larger for the y = a.x^2^ + b.x + c model, the r^2^ for y = a.x^2^ remains high at .9938. The optimal q for a y = a.x^q^ model becomes q = 1.94.

We note that the final figure, 6316, is extremely close to the 6300 value predicted by Dr. Wang Haibo based on the first 4 months of 2018 data.

### Comparison between Central Red Cross and COTRS data

The data maintained by the Red Cross Society of China and COTRS is supposed to be identical, each documenting the number of voluntary organ transplants that have taken place in China. When the two datasets are compared to one another, they are found to diverge in important respects, and in ways that do not accord with official explanations of how the datasets are generated.

There are two forms of divergence: discrepancies in donors and discrepancies in number of organs transplanted. Discrepancies are shown in Table 6.

**Table 6:** Selected Comparison of COTRS and Central Red Cross Data

Date	COTRS data		Central Red Cross data	
	Donors	Kidney & liver transplants	Donors	All organ transplants
12/31/2014	3150^a^	7833	–	–
1/18/2015	–	–	2996^a^	8326
12/31/2015	5916^b^	14,914	5862^b^	15,993
12/31/2016	9996	25,395	9996	27,613

[a-b] Inconsistent pairs of numbers.

**Fig. 8:**
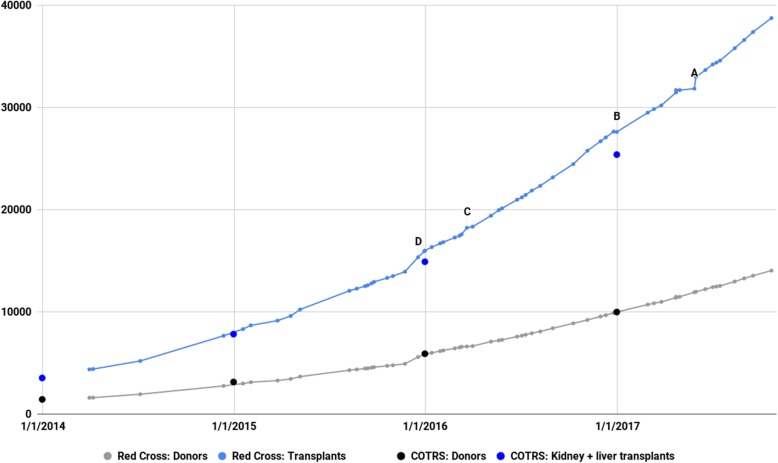
Cumulative Totals of COTRS and Central Red Cross Data

In Fig. 8, the full series of Central Red Cross data is plotted alongside COTRS 2017 figures.

The number of donors in the two datasets differs slightly until end 2016, when they converge; the number of organs transplanted is the opposite, with the figures corresponding at the beginning of 2015 before diverging at the beginning of 2016.

The gap is to be expected, given that COTRS 2017 data included only deceased liver and kidney transplants, whereas Red Cross data covers all solid organ transplants, including hearts and lungs. However, the number of lung and heart transplants performed in China does not come close to bridging this gap, as Table 7 shows.

**Table 7:** Transplant Totals for 2016

All organs^a^	Kidney and liver^b^	Lung^c^	Heart^c^	Remainder
11,620	10,481	204	380	555

^a^Central Red Cross data

^b^COTRS 2017

^c^Yang J. 黄洁夫委员:经济因素和专业医生缺乏成器官移植障碍. [Committee member Huang Jiefu: Economic factors and lack of professional doctors have become obstacles for organ transplantation]. China Central Television. 2017 March 7. Chinese. Available: http://news.cctv.com/2017/03/07/ARTInCnOadnD4BoBgEA7ES55170307.shtml Cited 1 Oct 2017

Table 7 shows that the unexplained gap in organs transplanted between the Central Red Cross and COTRS datasets in 2016 — other than kidney, liver, heart or lung — amounted to 555 (11620–10,481 - 204 - 380). This is a significant inconsistency between the two datasets.

This is noteworthy because at the end of 2016 the total number of *donors* matched perfectly between the COTRS 2017 and Red Cross figures (at 9996), and at the beginning of 2016 there was a discrepancy of only 54 donors, implying close synchronization between the two sources during that year (see Table 6).

### Local hospital activity

See Additional files 3 and 5.

## Discussion

While the analyzed transplant data presented in this manuscript raises severe doubts about the veracity of the claims of ethical reform in China, it is important to highlight qualitative indications that some hospitals in China are clearly engaged in the lifesaving work of voluntary organ procurement, allocation, and transplantation.

On December 31, 2015, Shanghai reported its 138th voluntary deceased donor. A television documentary featured the case of the 125th donor in the city, whose organs were procured at Huashan Hospital in November 2015; 40 doctors were involved in the procurement of organs from the donor; family members, doctors, and nurses were all interviewed [43].

Wuhan Tongji Hospital in Hubei Province reported the establishment of a full-time post for a transplant coordinator from January 2014, outlining the curriculum, the hours studied, the day to day work, and the increase in donations that resulted: from 98 donors in 2013, to 176 in 2015 [44]. Wuhan Tongji is one of the most advanced transplant hospitals in China, having acquired organs from brain dead donors since 2001 [45].

Jilin Province’s Red Cross reports of voluntary deceased transplants (183 donors by end March 2016) broadly correspond with hospital reports (about 220 donors by October 2016) [46].

The preceding examples are consistent with statements by leading Chinese medical administrators that most of the country’s voluntary donation activity takes place at a small number of major transplant centers [11, 47].

Modest success in organ transplant reform at a few key hospitals, however, is not all that Chinese medical officials have claimed. They have claimed a revolution in transplant practice across the country, with numbers of allegedly voluntary deceased donors growing at geometric rates and the cessation of all sourcing of organs from prisoners. It is these claims that are assessed in this report.

### Inferences from the data analysis

#### COTRS data

Analysis of the COTRS 2017 data shows an average divergence of less than 0.08% from perfect quadratic formulae. We believe the assumption that these numbers emerged by chance, or as a coincidence, from organ donations taking place across China, would require an unrealistic and implausible sequence of events persisting for 7 years.

The management of a voluntary organ donation, allocation, and transplantation system involves a highly complex set of processes and interactions between thousands of individuals at hundreds of locations over many years. Almost all instances of donation first require the occurrence of a brain injury, the diagnosis of brain death, the absence in the donor of numerous disqualifying diseases or contraindicating medications, preservation of the donor, gaining consent from the family to donate, blood and tissue typing, crossmatching, location of the recipient (through COTRS), their rapid conveyance to the hospital, multiple organ procurements, and then transplantations by separate medical teams. In many cases each donor triggers activities requiring the coordination of multiple hospitals. In China at the time of this study, 173 hospitals are licensed to perform voluntary donations, with thousands of doctors, nurses, and support staff, in a country of 1.4 billion people. Moreover, during the years in question the donation system was still being constructed. Doctors were being trained in the new diagnostic criteria for brain or circulatory deaths, nurses were being trained as transplantation coordinators, the public was being educated in the need to donate, families were making culturally difficult decisions about the disposition of the bodies of their deceased relatives, and the infrastructure for connecting potential donors with recipients, transplant teams, and other hospitals, was still being built.

Given all of these variables across multiple interlocking processes, the finding that China’s data for voluntary deceased donors, kidney transplants, and liver transplants, conform to three almost perfect quadratic equations is highly surprising. Genuine data generated from a complex, growing system with all the above caveats is not expected to exhibit such a sustained, extremely smooth growth curve.

Additional file 1 compares the R-squared statistics and the mean squared errors from a quadratic against comparable multi-year annual donor and transplant data from 50 other countries in the Global Observatory of Donation and Transplantation database, managed by the World Health Organization and Spain’s National Organization of Transplants. The scatter charts show that China’s R-squared statistics for fit to quadratic formulae are between one and two orders of magnitude outside the range of all the other data, and that the mean squared error for China does not conform to the pattern of any other country. Thus, the fundamental behavior of the Chinese data — not just the rapid growth rate in transplants it depicts — is qualitatively different to every other country for which there is comparable data.

We propose that the most plausible explanation for the COTRS 2017 data is manual and deliberate manipulation in order to fit a target donor rate, with a mathematical function selected as the most efficient way to both 1) reach this goal in an apparently natural manner, and 2) provide a common reference and guide for derivative data through the Chinese system. It is the exquisite precision of the fit of the data to the Procrustean Bed of a smooth mathematical formula that we believe rules out competing explanations.

A unique window of opportunity for the testing of our initial hypothesis was opened when Chinese authorities published updated COTRS data in July 2018.

The statistical analysis of COTRS 2018 data demonstrates that the extra datapoint strengthens the initial hypothesis of data falsification.

While we are aware that a range of values for 2017 would be in conformance with the model, it is highly significant that the value that did appear — 5146 — allowed for a major simplification and therefore strengthened the hypothesis of a mathematical model being used.

The significance of the new datapoint for deceased donors in 2017 is that it lies in a very restricted range that implies a more significant conformance not to a general quadratic equation, but to a one-parameter quadratic, of the form y = a.x^2^ (where x = 0 corresponds to 2010, the year in which Chinese authorities state they began the system of voluntary deceased organ allocation).

The analysis shows that the far more parsimonious model of y = a.x^2^ is just as powerful as y = a.x^2^ + b.x + c for explaining the growth curve. This model simplification significantly increases the leverage of the argument that the data was not generated from real world transplant activity.

In the Results, we further asked: *how surprising is it that b and c should become so insignificant*? Would this have happened for other 2017 values, or is 5146 in some sense special? This was tested by looking at scenarios where the 2017 value ranged from 4950 to 5350. For each value, the two models (y = a.x^2^ + b.x + c and y = a.x^2^) were both fitted, and the reduction in SSE (sum of squares of errors from the model) obtained by using the former instead of the latter was calculated.

The resulting graph (Fig. 3) gave a visual indication that the 2017 value (5146) was almost exactly the value that *most strongly* reinforces the model simplification to y = a.x^2^.

We extended the analysis yet further, probing whether the power function also appeared to be the result of a manmade mathematical model. While there is no reason why real world data would conform extremely closely to any power function y = a.x^q^ for any value of q, if it did there is no reason why the power q should be an integer such as q = 2 giving y = a.x^2^. Thus, we fitted a.x^q^ to the data, for q ranging from 1 to 3, and calculated the (adjusted) r^2^ for each q.

The resulting graph (Fig. 4) demonstrated that the optimal q went from y = a.x^2.07^ to y = a.x^2.01^ between COTRS 2017 and COTRS 2018 data, meaning that the optimal power model was shown to be virtually identical to y = a.x^2^, further reinforcing the inexplicable and unrealistic simplicity of the real world data.

The simpler the model required to explain the data, the more difficult it becomes to argue that it was generated through a random and complex series of organ donation and transplantation events — and the stronger the leverage in the argument that the data was in fact generated by a simple model in the first place.

A discussion of the statistical significance of these findings, newly possible due to the simplification of the model (which allowed a linear regression to be fitted), is available in Additional file 4.

The Red Cross 2019 data was found to remain consistent with a quadratic formula. While it was not as supportive of the simplified y = a.x^2^ model as COTRS 2018 was, it was still consistent with it with a high r^2^.

#### Central Red Cross data

Data from the China Organ Donation Administration Center, managed by the Red Cross Society of China, is supposed to provide third party witness to and registration of every voluntary transplant. It contains five identified internal anomalies, at least three of which we believe are extremely difficult to explain without human-directed manipulation of the dataset.

Anomaly A1, where only the date but not the data was updated between April 23 and 30 on china-organdonation.org, but www.codac.org.cn showed different data on April 23, is perplexing. It could be interpreted in multiple ways. On its face, if the codac.org.cn update is disregarded, it indicates that no transplant activity at all took place during the intervening 7 day period — a highly unlikely scenario. Or, again disregarding the conflicting data, it could be that the date change was merely a clerical error. Alternatively, the April 23 www.codac.org.cn update may have been the ‘real’ data intended for that date, and the china-organdonation.org update on April 23 an accidental revelation of the predetermined April 30 figures. We have no way of adjudicating between these possibilities, though we believe the accrual of such anomalies speaks to data integrity problems.

Anomaly A2 may have been transcription error.

Anomalies B, C, and D, while potentially having innocent explanations, have the effect of vectoring the internal relationship in the data for transplants per donor back to an arbitrary ratio of 2.75, raising questions as to whether the anomalies are signs of manipulated data.

Anomaly C is the most remarkable, in which for a 10-day period in March 2016, the dataset reports that 21.3 organs were obtained per donor, which is clearly impossible. The addition of this data again “corrected” the dataset to be in line with the arbitrary organs/donor ratio. This anomaly cannot be discounted, because each subsequent cumulative number has built into it this clearly impossible figure. To make the dataset coherent, a series of entries around Anomaly C on 3/20/2016 would need to be retroactively modified. Furthermore, the possibility of backlogs of COTRS data being entered into the database as an explanation for C and D is in direct contradiction to the required procedures of COTRS allocations.

While these anomalies now appear obvious, it is understandable that they have not been discovered until now, as the Central Red Cross data did not previously exist in a series. It is only after the full series was captured, archived, logged, and analyzed that these anomalies revealed themselves.

The extraordinary growth in registered volunteer donors also raises questions. Given the paucity of data on this at the local level, no other metric exists with which to compare the figures, and thus they cannot be invalidated outright. It is possible that the one-day leap of exactly 25,000 on 12/31/2015 was due to a batch upload of data on a single day — yet this pattern did not occur before or since, and was followed by steady growth through 2016. Then in 2016, again at the end of the year, the entire dataset doubles within the space of a week, from 12/25/2016–12/31/2016. These two sudden changes to the data raise questions about the integrity of the series.

Collectively, we maintain that these apparent human-directed alterations of the Central Red Cross data are consistent with the contention that, like the COTRS dataset, the data was not formed by the accretion of individual cases of successful voluntary organ donation and allocation, but instead was manually manipulated to fit the formula-derived COTRS master version, albeit with imperfect results.

#### Comparison between Central Red Cross and COTRS data

The comparison between the two datasets revealed two important discoveries. The first is that, in confirmation of official statements, they tend to have identical numbers of donors. Although this is not the case for several years, for reasons detailed later, the convergence is very close by the end of 2015 (with a discrepancy of 54 donors) and identical by the end of 2016, where the Central Red Cross database is updated to 9996 donors on the last day of the year, in line with COTRS. Thus, by end 2016 the datasets are in agreement to the day.

In contrast, the number of transplanted organs diverges, even after adding the national heart and lung transplants, which are not reported in the COTRS data. There is an inexplicable gap of 555 transplants for the year 2016.

A potential explanation for this gap that preserves the integrity of the data would be that it is filled by other solid organs — pancreatic and small intestine transplants. This explanation does not appear to hold, however. While data on transplants of these organs in China is difficult to come by, according to a 2011 Chinese medical paper, only 200 pancreas transplants had been performed since 1989 [48]. The vast majority, if not all of these, would have used organs from prisoner sources. Small intestine transplants are far rarer still. There is no subsequent data suggesting that pancreas transplants increased at the extremely high rate that would have been required to close the gap between the Red Cross and COTRS datasets, in particular under the more constrained conditions of voluntary donor sourcing. Thus, the gap in reports of the transplanted organs remains.

The finding of both a concordance between donors in the datasets, yet a persistent, inexplicable gap in organs, casts doubt on the integrity of both datasets. Had the number of donors not been identical, it may have been possible to reason that the two databases are in fact different in some fashion — for example that the Red Cross data captures some transplant activity, while the COTRS data captures other activity.

We believe that the inexplicable divergence supports the hypothesis that the datasets are updated by design, rather than real organ allocation data. Any such manual manipulation contains the potential for the kinds of discrepancies we have discovered.

#### Comparison between provincial Red Cross data and hospital activity

The examination of hospital-level transplant activity (detailed and discussed in Additional file 5) fails to disconfirm the data-based findings, but rather tends to corroborate them. While this finding cannot be conclusive due to the lack of transparency around hospital activity, we believe that the most plausible interpretation of the data is that it is part of a pattern of data fabrication extending to the provincial level.

#### General remarks about the data analysis

In light of the accumulation of findings above, we believe that two propositions can be advanced about the datasets under examination.

The first is that the unusual and anomalous features in the data are due to deliberate human intervention. We believe this is the only plausible explanation for the qualities identified in the COTRS, and central and provincial Red Cross data, which include mirroring of quadratic formulae, stubborn adherence to arbitrary ratios, anomalies that abrogate the mathematical integrity of data series, unsubstantiated growth patterns, and other irregularities. It is difficult to imagine how such data from three sources could have come to possess these qualities if not for deliberate, ongoing and imperfect human intervention.

The second is that this intervention could not have been piecemeal or without forethought. This proposition is based primarily on the COTRS datasets, which are comprised of three sets of seven (COTRS 2017), and one set of eight (COTRS 2018), data points that we believe were evidently derived from arbitrary mathematical formulae. Whatever the real data, this result was conceived as a single act: It could not be an accretion of manual updates, since the probability of accretive manipulations arriving at such a perfect mathematical function must be roughly equivalent to such an outcome being arrived at by natural activity. The other two data series — central and local Red Cross figures — have apparently been made in the image of this data. If the COTRS data was falsified through a top-down process, and the central and local Red Cross data adhere to the COTRS data, then they must have been derived in a similar fashion, with top-level coordination to maintain a semblance of congruence. We believe that the failure of congruence in organ transplants between Central Red Cross and COTRS figures, the simplification of the model between COTRS 2017 and COTRS 2018 series, and the failure of hospital activity to substantiate provincial Red Cross figures, all corroborate the hypothesis that the annual figures were handed down as quotas — albeit quotas that were imperfectly implemented across a fractured bureaucratic and administrative apparatus, thus exposing the discrepancies identified.

### Additional sources and considerations

The statistical forensics and data-based findings must be situated within the broader context of the challenges, obstacles, and distinctive features of China’s voluntary organ transplant reforms. In broad terms, these include the following, details of which may be found in Additional file 6:

1. Chinese authorities describe Chinese transplant data as a state secret; previously public transplant registries have been removed from public view; and custody of organ transplant registries has been transferred from Hong Kong to the mainland. Of the 12 officially-controlled transplant datasets we have identified, none are transparently displayed for public scrutiny; data from 3 can be manually and painstakingly collected (as this research shows), while 9 of the 12 are closed to the public entirely.
2. During the period of our study, Beijing municipality had not reported any Red Cross deceased donor data, despite being the capital and having the most number of, and most sophisticated, transplant hospitals in China. We believe this is contrary to claims of transparency.
3. During the period of our study, China had established no system of required referral for imminent deaths, while such systems are the lifeblood of any voluntary organ donation and allocation system. We believe this is a significant barrier to achieving the scale of voluntary donation activity claimed, and will need to be surmounted for China to develop a genuine voluntary, hospital-based organ donation system.
4. Key agencies in the transplant sector have been locked in bureaucratic turf battles and have, by the admission of top officials, failed to coordinate their activities.
5. A number of major hospitals report performing transplants for which they have no authorization from the NHFPC. We were unable to find reports of any sanctions for these open violations of regulations.
6. A number of leading transplant surgeons appear to have, whether deliberately or not, misclassified donors as “voluntary” where a range of other evidence indicates that such donors were not in fact voluntary.
7. Chinese hospitals continue to struggle with low rates of consent for organ donation, complicated by the fact that family members — not merely donors themselves — must provide positive (i.e. opt-in) consent.
8. China’s policy of ‘humanitarian aid’ often involves large cash payments to poor rural families, a violation of WHO guidelines.

## Conclusion

Any evaluation of the current state of the Chinese transplant sector is enormously complicated. Most of the data held by Chinese governmental agencies on the matter is not available for public inspection. We have compiled and analyzed the three national datasets that are available for collection.

The analysis has revealed that COTRS 2017 data are extremely close to quadratic functions, in other words that the series exhibit extraordinarily smooth growth rates. Subsequent analysis, following the drafting of our initial findings but prior to their being made public, revealed that updated COTRS 2018 data for deceased donors can be explained by an even more parsimonious mathematical model.

We believe that the only plausible explanation for this is that the data were man-made, based on a mathematical formula; we believe that the sheer smoothness of the growth curve precludes it from having resulted from voluntary transplant activity or from minor manipulations of real data. This is borne out by the comparison to every other country with comparable data in the GODT database.

The COTRS 2018 update significantly strengthened the initial findings by allowing a simplification of the mathematical model.

Further investigation of central and provincial Red Cross datasets corroborates the prediction. It was found that the Central Red Cross data exhibits several implausible anomalies, which undermine the integrity of the data and bear the hallmarks of human manipulation. These anomalies are not explicable as isolated transcription errors, as a significant amount of retrospective modification to the cumulative series would be required for the anomalies to be removed. The anomalies were also found to be consistently associated with a rapid return of the cumulative organs/donor to an arbitrary rate of 2.75.

Provincial Red Cross data (see Additional file 5) was found to exhibit various anomalies including implausible rates associated with transplants and inconsistencies with hospital-based donation reports. Some of the anomalies strongly imply significant use of nonvoluntary donors.

We believe that, given current information, the only plausible explanation that accounts for all of our observations is that the three datasets were manufactured and manipulated from the central levels of the Chinese medical bureaucracy. The goal of these elaborate efforts appears to have been to create a misleading impression to the international transplantation community about the successes of China’s voluntary organ donation reform, and to neutralize the criticism of activists who allege that crimes against humanity have been committed in the acquisition of organs for transplant.

We wish to make clear that we have no information about the individuals who may have orchestrated these efforts of data falsification, nor do we attribute any such activity to any named parties, or to the named Chinese officials who publicly disseminated the data.

The incongruence between the datasets, which sometimes resulted in contradictions, appears to demonstrate the difficulty of the complex task of falsification across three datasets involving numerous bureaucratic actors with sometimes competing institutional interests. Qualitative analysis of the systemic challenges and contingencies involved in procuring and allocating voluntarily-donated organs in Chinese hospitals adds further weight to these findings.

While we consider that central-level data falsification has clearly taken place, we have found that genuine efforts of voluntary organ donations are also underway, which we consider to be an encouraging development. At the same time, the large cash sums offered to poor rural families to donate their relative’s organs (documented in Additional file 6) raise concerns about financial coercion and violations of World Health Organization organ donation guidelines.

The current voluntary system appears to operate alongside the continued use of nonvoluntary donors (most plausibly prisoners) who are misclassified as “voluntary.” (see Additional file 6.)

Thus, rather than the solely prisoner-based organ transplant system of years past, or the untarnished voluntary system promised by officials, the available evidence indicates in our view that China has a complex hybrid transplant program: voluntary donations, incentivized by large cash payments, are apparently used alongside nonvoluntary donors who are marked down as citizen donors.

Based on the data we have examined, there is no way of determining the proportion of each of these organ sources, nor of China’s total transplant volume. Our analysis of cases of misclassification (see Additional file 6) found that the apparent prisoner donors were up to seven times more numerous than the apparently legitimate voluntary donors, though there is no basis for extrapolating these instances to every hospital, nor for extrapolating such data to the actual number of transplants. Like many forms of enterprise in China, there is likely significant regional variation.

China’s apparent systematic falsification of national organ donation data severely undermines the good faith efforts being made to integrate China into the international transplantation community. The World Health Organization, The Transplantation Society, the Declaration of Istanbul Custodian Group, and the Pontifical Academy of Sciences have all provided endorsements of the reforms based on what appears to be contaminated data. Medical journals have published transplant data on the good faith assumption that their Chinese counterparts have not falsified the true source of organs.

We believe that one of the most troubling consequences of the apparent data falsification and apparent continued use of nonvoluntary organs in the official allocation mechanisms is that it impugns the reputations of Chinese surgeons dedicated to the highest standards in ethical transplant medicine, and undermines their efforts at establishing a trustworthy, transparent, and ethical system.

While it appears from our research that legitimate voluntary organ donation is indeed taking place in some hospitals, the corruption of the process with misclassified nonvoluntary donors makes it almost impossible to determine whether any particular claim of ethical organ sourcing is indeed what it purports to be.

In recent years, following decades of scorn for practices that violated widely-held standards of medical ethics, Chinese transplant surgeons have been welcomed back into the international transplantation community, able to attend conferences and publish in respected journals. China’s return to the fold was predicated on a revolution in organ sourcing practice, supported by the data we have forensically analyzed. Given that this data appears to have been falsified, international medical organizations may wish to reassess their stance. The welcoming of China’s organ transplantation system into the international medical community has been based on trust; in light of our findings, we believe this trust has been violated.

## Supplementary information

**Additional file 1.** Statistical comparison of China’s transplant data with comparable data from 50 other countries in GODT database.

**Additional file 2.** Central Red Cross data.

**Additional file 3.** Provincial Red Cross data.

**Additional file 4.** Comments on statistical significance from the analysis of COTRS 2018 data.

**Additional file 5.** Results and Discussion of analysis of Red Cross and hospital-level data in five provinces.

**Additional file 6.** Additional sources and considerations regarding China’s deceased organ donation system.

## Data Availability

The datasets supporting the conclusions of this article are included within the article and its additional files. Data for COTRS 2017, Central Red Cross, and local Red Cross datasets was collected until the end of October 2017. COTRS 2018 data was collected until July 2018.

## Abbreviations

CODAC: China Organ Donation Administrative Center
COTRS: China Organ Transplant Response System
DICG: Declaration of Istanbul Custodian Group
GODT: Global Observatory on Donation and Transplantation
ICUs: Intensive care units
NHFPC: National Health and Family Planning Commission’s
OPOs: Organ procurement organizations
PAS: Pontifical Academy of Sciences
TTS: The Transplantation Society
WHO: World Health Organization
