# Supplementary material for: Analysis of official deceased organ donation data casts doubt on the credibility of China’s organ transplant reform
Source: BMC Med Ethics. 2019 Nov 14;20:79. doi: 10.1186/s12910-019-0406-6 (PMC6854896; doi:10.1186/s12910-019-0406-6)
Supplement: Supplementary file 1 — Additional file 1. Statistical comparison of China’s transplant data with comparable data from 50 other countries in GODT database. [file 12910_2019_406_MOESM1_ESM.docx]

# **Additional file 1. Statistical comparison of China’s transplant data with comparable data from 50 other countries in GODT database.**

The COTRS 2017 data contains total annual deceased voluntary donors, deceased voluntary kidney transplants, and deceased voluntary liver transplants in China over 7 years. The data show extremely close fits to quadratic lines. The data is plotted in Fig 1 with quadratic least squares lines of best fit.

Table A shows the equations of the fitted lines in Fig 1 in the manuscript.

**Table A. Equations for the lines in Fig 1.** *x* is the number of years since 2009.

| Deceased donors | *y = 128.2x^2^ - 358.65x + 298.57* |
| --- | --- |
| Deceased kidney transplants | *y = 225.7x^2^ - 615.35x + 462.28* |
| Deceased liver transplants | *y = 107.88x^2^ - 335.33x + 295 = 0* |

This appendix compares the COTRS 2017 data to the best available comparable data from 50 other countries with the aim of assessing how unusual the closeness to fit is.

We compare China’s data to comparable data from other countries, rather than directly assess the statistical significance of the *R^2^s* for China’s data, for technical reasons: assessing that China’s data is *too close* to a simple mathematical formula does not lend itself well to formal hypothesis testing, where typically the initial presumption is that data *does* conform to a certain well-defined behavior (in this case a quadratic formula), and the test is aimed at determining whether the specific data implies it *is not close enough*.

In this instance, there is no well-defined way of expressing how erratically a rapidly developing voluntary organ procurement and transplantation system should grow. We considered whether it could be shown that the expected random fluctuations of availability in deceased donors alone (which can be modelled if one makes technical assumptions of randomness and independence) would be sufficient to prove that the *R^2^s* are too close to 1, but found that this was not the case. This analysis is therefore directed at investigating the plausibility of a near perfect quadratic growth in infrastructure, which by its nature is unquantifiable.

This meant that this part of our analysis was heuristic and our aim was to establish if the smoothness of China’s data showed a compelling divergence from the behaviour of the 50 other countries in its smoothness of industry growth. Establishing this would not in isolation be a statistical proof of the falsification of data, but rather would add weight to the overall argument presented in this paper.

Because of this, the most appropriate method of assessing the smoothness of China’s data (i.e. the closeness of its adherence to mathematical formulae) is to compare it to similar real world data.

The data of other countries was obtained from the Global Observatory on Donation and Transplantation (GODT) via the database at <http://www.transplant-observatory.org/export-database>. The data series ‘Total Actual DD’, ‘DD Kidney Tx’ and ‘DD Liver Tx’ were used. Every country was tested to see if it had seven consecutive years of data for all three series. If so, the *earliest* seven continuous years of data was selected for inclusion in the analysis. Fifty countries satisfied this condition. These formed the basis for comparison with the seven continuous years of China’s data.

For each country, the quadratic least squares line of best fit were obtained for all three series. This line minimizes the Root Mean Square Error (RMS Error) and maximizes the *R^2^* statistic which is defined by:

[
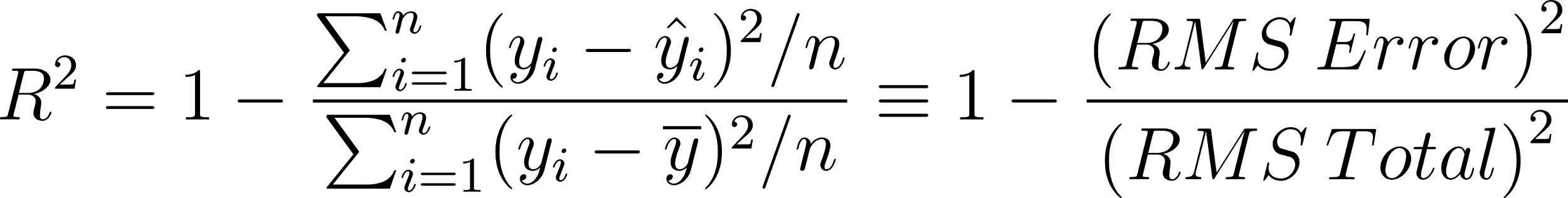
](https://www.codecogs.com/eqnedit.php?latex=R%5E2%3D1-%5Cfrac%7B%5Csum_%7Bi%3D1%7D%5E%7Bn%7D%20(y_i-%5Chat%7By%7D_i)%5E2%2Fn%7D%7B%5Csum_%7Bi%3D1%7D%5E%7Bn%7D%20(y_i-%5Coverline%7By%7D)%5E2%2Fn%7D%5Cequiv%201-%5Cfrac%7B%5Cleft%20(%20RMS%5C%3A%20Error%20%5Cright%20)%5E2%7D%7B%5Cleft%20(%20RMS%5C%3A%20Total%20%5Cright%20)%5E2%7D)

where

[
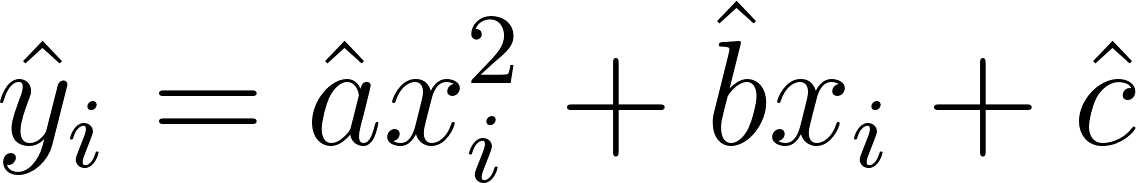
](https://www.codecogs.com/eqnedit.php?latex=%5Chat%7By%7D_i%20%3D%20%5Chat%7Ba%7Dx_i%5E2%20%2B%20%5Chat%7Bb%7Dx_i%20%2B%5Chat%7Bc%7D%20)

are the fitted values of the data [
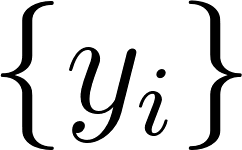
](https://www.codecogs.com/eqnedit.php?latex=%5C%7By_i%5C%7D) and years [
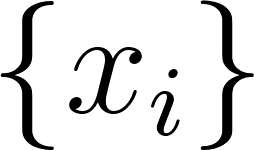
](https://www.codecogs.com/eqnedit.php?latex=%5C%7Bx_i%5C%7D).

The Root Mean Square Total (RMS Total) is a measure of the total variability between the data values [
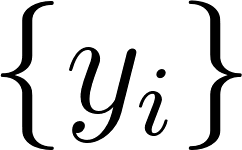
](https://www.codecogs.com/eqnedit.php?latex=%5C%7By_i%5C%7D). The RMS Error is a measure of the differences between the data values [
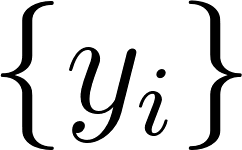
](https://www.codecogs.com/eqnedit.php?latex=%5C%7By_i%5C%7D) and the fitted values [
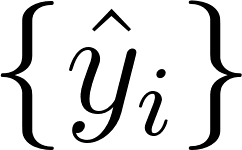
](https://www.codecogs.com/eqnedit.php?latex=%5C%7B%5Chat%7By%7D_i%5C%7D).

Each line of best fit has only four degrees of freedom, and so we would expect some of the fits to have high *R^2^* values. For China the *R^2^* of these three series are .99925, .99952 and .9989 for donors, kidneys and livers respectively. These correspond to distances of .075%, .048% and .11% from a perfect *R^2^* of 1. These are at least one order of magnitude closer to a perfect *R^2^* of 1 than any of the series for any other country, the closest of which is 1.30%, and represent an extraordinarily close fit to the quadratic equation. This can be seen graphically in Fig A below.

**Fig A. RMS Total by 1-R^2^ for Fifty Countries and China.** Each country has 3 points for donors, kidneys and livers. Horizontal and vertical scales are both logarithmic as the data spans several orders of magnitude. The three points annotated with a ‘C’ are those of China.


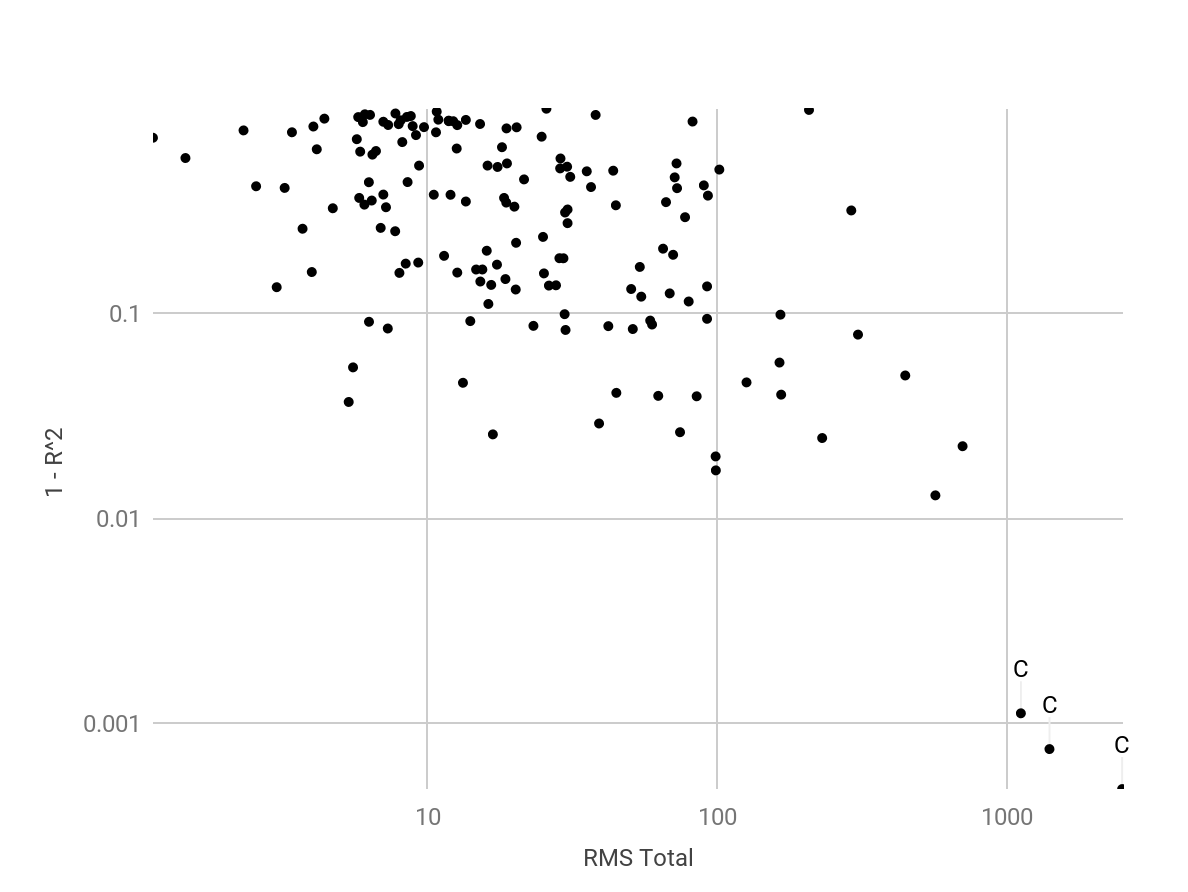


The vertical axis is *1-R^2^*, where a value of zero indicates a perfect fit, and is presented in a log scale to better reflect proximity to zero. The Root Mean Square Total (RMS Total) is on the horizontal axis to see how *1-R^2^* might change as a result of higher variability in the data. The chart shows clearly that for China the *1-R^2^* values are the smallest of all countries while the RMS Totals are the largest. Also observe that for all countries other than China, there is very little change in the typical vertical distribution of values of *1-R^2^* as RMS Total changes, just a slight decrease and a slightly greater spread where RMS Total is greater. For China, however, this slight trend does not hold as its values of *1-R^2^* are significantly lower.

However, China has distinct properties that should be taken into account as they could potentially confound this analysis. China has one of the largest transplant industries, while also being a rapidly growing industry, which makes it unique. These qualities can, for technical reasons, contribute to a larger *R^2^*. On the other hand, it could also be argued that such a rapid growth in infrastructure, for practical reasons, is inconsistent with a very smooth rate of growth.

Because the properties of industry size and industry growth have a direct impact on *R^2^*, the comparisons with other countries will be more transparent if RMS Error is compared with RMS Total. For this, the following Fig B was produced.

**Fig B. RMS Total by RMS Error for Fifty Countries and China.** Each country has 3 points for donors, kidneys and livers. Horizontal and vertical scales are both logarithmic as the data spans several orders of magnitude. The three points annotated with a ‘C’ are those of China.


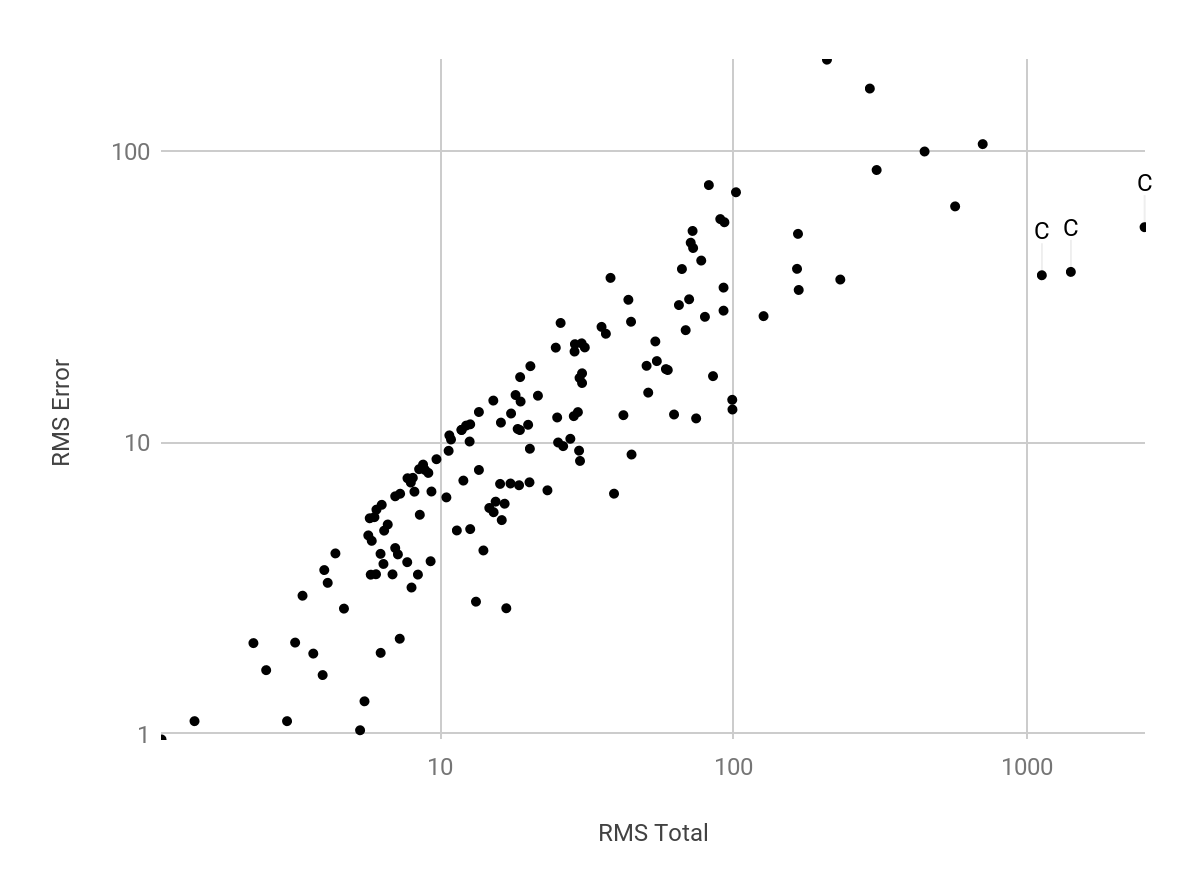


As expected, the RMS Error increases with an increase in RMS Total. For all countries other than China, the data consistently falls in a narrow band rising at 45 degrees. China, however, is a distinct outlier from this, lying below the band. This indicates that the absolute size of the errors are significantly below what would be expected given the size of its transplant sector.

**Table B. GODT dataset used for Fig A and Fig B scatterplots, with calculated *1-R^2^*, RMS Total, and RMS Error figures.** All data (except China’s) was obtained from the GODT database; ‘Donors’ is ‘Total Actual DD’ per the GODT categories; ‘Kidneys’ is ‘DD Kidney Tx’ and ‘Livers’ is ‘DD Liver Tx’.

| **Country** | **Category** | **Start Year** | **Year 1** | **Year 2** | **Year 3** | **Year 4** | **Year 5** | **Year 6** | **Year 7** | **1 - R^2^** | **RMS Total** | **RMS Error** |
| --- | --- | --- | --- | --- | --- | --- | --- | --- | --- | --- | --- | --- |
| China (COTRS) | Donors | 2010 | 34 | 132 | 433 | 849 | 1702 | 2766 | 4080 | 7.49E-04 | 1,406.44 | 38.49 |
| China (COTRS) | Kidneys | 2010 | 33 | 166 | 743 | 1533 | 2986 | 4931 | 7224 | 4.78E-04 | 2,507.59 | 54.82 |
| China (COTRS) | Livers | 2010 | 30 | 94 | 318 | 629 | 1301 | 2150 | 3257 | 1.12E-03 | 1,120.27 | 37.49 |
| Argentina | Donors | 2004 | 403 | 407 | 462 | 486 | 519 | 500 | 583 | 9.23E-02 | 58.73 | 17.85 |
| Argentina | Kidneys | 2004 | 573 | 607 | 662 | 714 | 753 | 839 | 846 | 1.72E-02 | 99.00 | 12.97 |
| Argentina | Livers | 2004 | 197 | 208 | 236 | 247 | 247 | 245 | 289 | 1.37E-01 | 27.76 | 10.29 |
| Australia | Donors | 2007 | 198 | 259 | 247 | 302 | 337 | 354 | 391 | 3.96E-02 | 62.61 | 12.47 |
| Australia | Kidneys | 2007 | 342 | 424 | 446 | 548 | 570 | 606 | 630 | 2.01E-02 | 98.83 | 14.00 |
| Australia | Livers | 2007 | 147 | 192 | 185 | 204 | 213 | 227 | 248 | 8.31E-02 | 29.95 | 8.64 |
| Austria | Donors | 2000 | 194 | 191 | 195 | 189 | 185 | 203 | 207 | 3.30E-01 | 7.18 | 4.12 |
| Austria | Kidneys | 2000 | 357 | 362 | 368 | 336 | 348 | 371 | 374 | 6.38E-01 | 12.60 | 10.07 |
| Austria | Livers | 2000 | 151 | 126 | 151 | 141 | 126 | 133 | 137 | 8.12E-01 | 9.72 | 8.75 |
| Belgium | Donors | 2000 | 256 | 222 | 223 | 237 | 227 | 248 | 282 | 1.31E-01 | 20.15 | 7.29 |
| Belgium | Kidneys | 2000 | 448 | 358 | 334 | 413 | 353 | 358 | 445 | 4.98E-01 | 43.77 | 30.87 |
| Belgium | Livers | 2000 | 136 | 174 | 196 | 239 | 204 | 190 | 218 | 2.76E-01 | 30.44 | 15.99 |
| Brazil | Donors | 2004 | 1408 | 1688 | 1112 | 1049 | 1331 | 1553 | 1934 | 3.18E-01 | 290.73 | 164.03 |
| Brazil | Kidneys | 2004 | 1846 | 1878 | 1520 | 1883 | 2033 | 2524 | 2946 | 4.98E-02 | 446.67 | 99.72 |
| Brazil | Livers | 2004 | 780 | 872 | 1020 | 978 | 1053 | 1197 | 1295 | 5.76E-02 | 164.26 | 39.43 |
| Bulgaria | Donors | 2005 | 6 | 19 | 9 | 8 | 11 | 20 | 4 | 9.09E-01 | 5.76 | 5.49 |
| Bulgaria | Kidneys | 2005 | 8 | 30 | 11 | 8 | 17 | 36 | 8 | 9.67E-01 | 10.75 | 10.57 |
| Bulgaria | Livers | 2005 | 3 | 9 | 6 | 5 | 9 | 13 | 3 | 7.66E-01 | 3.40 | 2.97 |
| Canada | Donors | 2000 | 475 | 420 | 411 | 428 | 414 | 414 | 468 | 2.36E-01 | 25.04 | 12.18 |
| Canada | Kidneys | 2000 | 764 | 661 | 627 | 650 | 603 | 612 | 773 | 2.07E-01 | 65.06 | 29.61 |
| Canada | Livers | 2000 | 404 | 297 | 339 | 335 | 367 | 421 | 422 | 3.37E-01 | 44.69 | 25.96 |
| Chile | Donors | 2005 | 134 | 154 | 135 | 119 | 111 | 92 | 113 | 3.48E-01 | 18.69 | 11.02 |
| Chile | Kidneys | 2005 | 234 | 263 | 228 | 206 | 199 | 163 | 195 | 3.11E-01 | 29.84 | 16.64 |
| Chile | Livers | 2005 | 67 | 86 | 75 | 74 | 69 | 60 | 78 | 9.47E-01 | 7.74 | 7.53 |
| Colombia | Donors | 2008 | 436 | 554 | 569 | 392 | 373 | 329 | 346 | 4.22E-01 | 89.97 | 58.44 |
| Colombia | Kidneys | 2008 | 641 | 777 | 798 | 727 | 693 | 587 | 643 | 4.62E-01 | 71.36 | 48.49 |
| Colombia | Livers | 2008 | 198 | 237 | 218 | 184 | 211 | 178 | 201 | 8.00E-01 | 18.73 | 16.75 |
| Croatia | Donors | 2000 | 12 | 32 | 41 | 39 | 59 | 44 | 57 | 1.64E-01 | 14.71 | 5.95 |
| Croatia | Kidneys | 2000 | 24 | 61 | 77 | 73 | 106 | 85 | 106 | 1.37E-01 | 26.24 | 9.71 |
| Croatia | Livers | 2000 | 6 | 18 | 24 | 28 | 43 | 39 | 45 | 4.59E-02 | 13.24 | 2.84 |
| Cuba | Donors | 2004 | 159 | 126 | 157 | 200 | 186 | 136 | 104 | 4.64E-01 | 31.09 | 21.19 |
| Cuba | Kidneys | 2004 | 169 | 115 | 111 | 150 | 136 | 130 | 111 | 8.10E-01 | 20.30 | 18.27 |
| Cuba | Livers | 2004 | 26 | 20 | 29 | 39 | 28 | 23 | 22 | 6.16E-01 | 5.85 | 4.59 |
| Czech Republic | Donors | 2003 | 191 | 211 | 207 | 193 | 217 | 198 | 200 | 8.21E-01 | 8.88 | 8.05 |
| Czech Republic | Kidneys | 2003 | 352 | 404 | 384 | 362 | 360 | 305 | 346 | 5.71E-01 | 28.77 | 21.74 |
| Czech Republic | Livers | 2003 | 64 | 82 | 82 | 98 | 115 | 97 | 102 | 1.64E-01 | 15.45 | 6.26 |
| Denmark | Donors | 2000 | 68 | 70 | 73 | 75 | 64 | 63 | 62 | 3.26E-01 | 4.70 | 2.69 |
| Denmark | Kidneys | 2000 | 121 | 121 | 132 | 129 | 135 | 121 | 120 | 3.66E-01 | 5.80 | 3.51 |
| Denmark | Livers | 2000 | 24 | 32 | 38 | 37 | 41 | 41 | 36 | 5.46E-02 | 5.53 | 1.29 |
| Dominican Republic | Donors | 2008 | 102 | 9 | 11 | 17 | 17 | 17 | 29 | 3.22E-01 | 30.44 | 17.27 |
| Dominican Republic | Kidneys | 2008 | 7 | 17 | 16 | 22 | 21 | 23 | 40 | 1.77E-01 | 9.28 | 3.91 |
| Dominican Republic | Livers | 2008 | 1 | 2 | 3 | 9 | 6 | 4 | 6 | 4.18E-01 | 2.56 | 1.65 |
| Ecuador | Donors | 2009 | 17 | 31 | 31 | 54 | 63 | 50 | 63 | 1.38E-01 | 16.58 | 6.16 |
| Ecuador | Kidneys | 2009 | 33 | 60 | 52 | 89 | 93 | 82 | 112 | 1.57E-01 | 25.22 | 9.99 |
| Ecuador | Livers | 2009 | 1 | 18 | 15 | 13 | 23 | 23 | 12 | 3.81E-01 | 7.03 | 4.34 |
| Estonia | Donors | 2007 | 25 | 31 | 33 | 23 | 22 | 32 | 32 | 8.93E-01 | 4.40 | 4.16 |
| Estonia | Kidneys | 2007 | 47 | 54 | 49 | 35 | 40 | 57 | 47 | 8.63E-01 | 7.03 | 6.53 |
| Estonia | Livers | 2007 | 2 | 2 | 4 | 3 | 8 | 9 | 9 | 1.34E-01 | 3.01 | 1.10 |
| Finland | Donors | 2000 | 103 | 88 | 89 | 53 | 109 | 85 | 109 | 6.48E-01 | 18.07 | 14.54 |
| Finland | Kidneys | 2000 | 187 | 165 | 169 | 156 | 192 | 161 | 207 | 5.19E-01 | 17.44 | 12.56 |
| Finland | Livers | 2000 | 31 | 38 | 47 | 43 | 50 | 43 | 53 | 2.62E-01 | 6.88 | 3.52 |
| France | Donors | 2003 | 1119 | 1291 | 1371 | 1443 | 1601 | 1610 | 1543 | 4.02E-02 | 166.46 | 33.37 |
| France | Kidneys | 2003 | 1991 | 2259 | 2375 | 2484 | 2676 | 2663 | 2603 | 2.47E-02 | 230.65 | 36.24 |
| France | Livers | 2003 | 791 | 883 | 975 | 1001 | 1043 | 1001 | 1035 | 3.94E-02 | 85.00 | 16.88 |
| Germany | Donors | 2003 | 1133 | 1075 | 1220 | 1259 | 1313 | 1199 | 1217 | 4.09E-01 | 72.72 | 46.50 |
| Germany | Kidneys | 2003 | 2111 | 1989 | 2190 | 2254 | 2340 | 2188 | 2172 | 5.04E-01 | 101.78 | 72.25 |
| Germany | Livers | 2003 | 781 | 817 | 898 | 980 | 1096 | 1067 | 1119 | 4.61E-02 | 126.35 | 27.13 |
| Greece | Donors | 2000 | 20 | 32 | 65 | 71 | 66 | 89 | 79 | 8.70E-02 | 23.20 | 6.84 |
| Greece | Kidneys | 2000 | 32 | 74 | 107 | 134 | 116 | 167 | 144 | 8.68E-02 | 42.09 | 12.40 |
| Greece | Livers | 2000 | 10 | 18 | 21 | 24 | 29 | 34 | 27 | 8.45E-02 | 7.28 | 2.12 |
| Hungary | Donors | 2000 | 138 | 137 | 167 | 161 | 160 | 181 | 177 | 2.02E-01 | 16.00 | 7.20 |
| Hungary | Kidneys | 2000 | 250 | 259 | 289 | 299 | 284 | 300 | 296 | 1.47E-01 | 18.57 | 7.13 |
| Hungary | Livers | 2000 | 23 | 19 | 17 | 31 | 43 | 44 | 45 | 1.91E-01 | 11.40 | 4.98 |
| Iran (Islamic Republic of) | Donors | 2000 | 20 | 43 | 52 | 87 | 114 | 118 | 128 | 2.91E-02 | 39.11 | 6.67 |
| Iran (Islamic Republic of) | Kidneys | 2000 | 32 | 70 | 96 | 167 | 207 | 209 | 243 | 2.63E-02 | 74.46 | 12.09 |
| Iran (Islamic Republic of) | Livers | 2000 | 5 | 16 | 22 | 49 | 66 | 53 | 97 | 9.92E-02 | 29.73 | 9.37 |
| Ireland | Donors | 2007 | 88 | 81 | 90 | 58 | 93 | 78 | 86 | 8.83E-01 | 10.89 | 10.23 |
| Ireland | Kidneys | 2007 | 141 | 136 | 154 | 151 | 165 | 131 | 147 | 7.66E-01 | 10.69 | 9.35 |
| Ireland | Livers | 2007 | 59 | 58 | 64 | 38 | 61 | 50 | 55 | 8.76E-01 | 8.07 | 7.56 |
| Israel | Donors | 2001 | 59 | 64 | 43 | 60 | 62 | 68 | 61 | 8.31E-01 | 7.31 | 6.66 |
| Israel | Kidneys | 2001 | 172 | 97 | 55 | 70 | 90 | 99 | 59 | 4.14E-01 | 36.68 | 23.61 |
| Israel | Livers | 2001 | 47 | 52 | 26 | 39 | 43 | 53 | 37 | 9.20E-01 | 8.75 | 8.39 |
| Italy | Donors | 2003 | 1042 | 1203 | 1197 | 1234 | 1194 | 1201 | 1273 | 3.50E-01 | 66.61 | 39.38 |
| Italy | Kidneys | 2003 | 1489 | 1746 | 1671 | 1665 | 1585 | 1533 | 1650 | 8.64E-01 | 82.25 | 76.47 |
| Italy | Livers | 2003 | 837 | 1016 | 1053 | 1091 | 1041 | 996 | 1061 | 2.95E-01 | 77.50 | 42.10 |
| Japan | Donors | 2005 | 91 | 112 | 105 | 109 | 105 | 105 | 112 | 6.21E-01 | 6.63 | 5.22 |
| Japan | Kidneys | 2005 | 160 | 197 | 187 | 210 | 189 | 189 | 212 | 5.27E-01 | 16.11 | 11.69 |
| Japan | Livers | 2005 | 4 | 5 | 10 | 13 | 7 | 7 | 41 | 3.80E-01 | 12.00 | 7.39 |
| Lithuania | Donors | 2006 | 33 | 48 | 33 | 50 | 36 | 39 | 41 | 9.31E-01 | 6.32 | 6.10 |
| Lithuania | Kidneys | 2006 | 54 | 83 | 46 | 75 | 63 | 72 | 76 | 8.68E-01 | 12.26 | 11.42 |
| Lithuania | Livers | 2006 | 3 | 8 | 6 | 7 | 13 | 12 | 15 | 1.59E-01 | 3.98 | 1.59 |
| Malaysia | Donors | 2006 | 25 | 25 | 13 | 18 | 18 | 23 | 18 | 6.33E-01 | 4.14 | 3.29 |
| Malaysia | Kidneys | 2006 | 13 | 24 | 22 | 34 | 34 | 40 | 29 | 1.75E-01 | 8.40 | 3.52 |
| Malaysia | Livers | 2006 | 4 | 5 | 4 | 6 | 3 | 7 | 7 | 5.74E-01 | 1.46 | 1.10 |
| Mexico | Donors | 2005 | 141 | 372 | 351 | 332 | 312 | 315 | 356 | 5.40E-01 | 72.41 | 53.21 |
| Mexico | Kidneys | 2005 | 276 | 505 | 526 | 562 | 495 | 484 | 579 | 3.76E-01 | 92.95 | 57.00 |
| Mexico | Livers | 2005 | 57 | 92 | 87 | 88 | 73 | 72 | 95 | 8.30E-01 | 12.66 | 11.54 |
| Netherlands | Donors | 2000 | 202 | 187 | 202 | 239 | 247 | 239 | 211 | 4.51E-01 | 21.53 | 14.46 |
| Netherlands | Kidneys | 2000 | 369 | 337 | 361 | 406 | 423 | 422 | 378 | 5.21E-01 | 30.33 | 21.88 |
| Netherlands | Livers | 2000 | 126 | 107 | 109 | 101 | 106 | 114 | 94 | 5.27E-01 | 9.34 | 6.78 |
| New Zealand | Donors | 2003 | 40 | 40 | 29 | 25 | 38 | 31 | 43 | 4.37E-01 | 6.27 | 4.14 |
| New Zealand | Kidneys | 2003 | 67 | 58 | 51 | 41 | 65 | 53 | 54 | 6.86E-01 | 8.17 | 6.77 |
| New Zealand | Livers | 2003 | 34 | 36 | 23 | 24 | 30 | 17 | 33 | 5.96E-01 | 6.45 | 4.98 |
| Norway | Donors | 2005 | 76 | 76 | 94 | 98 | 102 | 102 | 127 | 1.11E-01 | 16.21 | 5.41 |
| Norway | Kidneys | 2005 | 142 | 132 | 174 | 180 | 188 | 180 | 229 | 1.86E-01 | 29.46 | 12.70 |
| Norway | Livers | 2005 | 38 | 62 | 72 | 79 | 82 | 89 | 89 | 2.57E-02 | 16.80 | 2.69 |
| Poland | Donors | 2000 | 410 | 450 | 490 | 525 | 562 | 556 | 496 | 8.40E-02 | 51.14 | 14.82 |
| Poland | Kidneys | 2000 | 780 | 843 | 910 | 984 | 1045 | 1040 | 899 | 1.36E-01 | 92.32 | 34.00 |
| Poland | Livers | 2000 | 73 | 88 | 135 | 156 | 181 | 199 | 180 | 4.10E-02 | 44.86 | 9.08 |
| Portugal | Donors | 2000 | 194 | 202 | 217 | 190 | 222 | 190 | 201 | 8.73E-01 | 11.82 | 11.04 |
| Portugal | Kidneys | 2000 | 346 | 359 | 367 | 305 | 392 | 330 | 358 | 9.99E-01 | 25.72 | 25.71 |
| Portugal | Livers | 2000 | 162 | 181 | 190 | 172 | 200 | 185 | 223 | 3.66E-01 | 18.37 | 11.12 |
| Republic of Korea | Donors | 2005 | 91 | 141 | 148 | 148 | 261 | 268 | 268 | 1.25E-01 | 68.59 | 24.29 |
| Republic of Korea | Kidneys | 2005 | 4 | 263 | 280 | 289 | 488 | 491 | 491 | 9.87E-02 | 165.51 | 52.00 |
| Republic of Korea | Livers | 2005 | 4 | 118 | 122 | 125 | 236 | 232 | 232 | 1.14E-01 | 79.77 | 26.99 |
| Romania | Donors | 2000 | 25 | 21 | 13 | 8 | 10 | 11 | 22 | 9.11E-02 | 6.27 | 1.89 |
| Romania | Kidneys | 2000 | 22 | 40 | 24 | 16 | 19 | 19 | 39 | 7.42E-01 | 9.12 | 7.85 |
| Romania | Livers | 2000 | 8 | 8 | 14 | 7 | 7 | 5 | 17 | 8.17E-01 | 4.03 | 3.64 |
| Saudi Arabia | Donors | 2004 | 54 | 62 | 102 | 80 | 105 | 68 | 95 | 5.40E-01 | 18.80 | 13.81 |
| Saudi Arabia | Kidneys | 2004 | 88 | 95 | 151 | 122 | 166 | 111 | 156 | 5.11E-01 | 28.70 | 20.50 |
| Saudi Arabia | Livers | 2004 | 29 | 28 | 49 | 54 | 59 | 51 | 62 | 1.58E-01 | 12.66 | 5.04 |
| Singapore | Donors | 2005 | 21 | 30 | 26 | 27 | 25 | 26 | 20 | 4.10E-01 | 3.21 | 2.05 |
| Singapore | Kidneys | 2005 | 43 | 56 | 46 | 46 | 41 | 36 | 36 | 3.56E-01 | 6.41 | 3.82 |
| Singapore | Livers | 2005 | 2 | 7 | 12 | 17 | 17 | 15 | 16 | 3.70E-02 | 5.34 | 1.03 |
| Slovakia | Donors | 2008 | 77 | 86 | 91 | 69 | 71 | 60 | 64 | 3.80E-01 | 10.50 | 6.47 |
| Slovakia | Kidneys | 2008 | 145 | 153 | 162 | 116 | 130 | 109 | 110 | 3.32E-01 | 19.94 | 11.49 |
| Slovakia | Livers | 2008 | 12 | 24 | 33 | 25 | 29 | 22 | 23 | 3.40E-01 | 6.05 | 3.53 |
| Slovenia | Donors | 2000 | 22 | 23 | 35 | 28 | 36 | 21 | 30 | 7.09E-01 | 5.69 | 4.79 |
| Slovenia | Kidneys | 2000 | 44 | 47 | 55 | 43 | 55 | 28 | 48 | 9.10E-01 | 8.48 | 8.09 |
| Slovenia | Livers | 2000 | 10 | 9 | 11 | 9 | 15 | 13 | 8 | 7.82E-01 | 2.31 | 2.05 |
| South Africa | Donors | 2005 | 74 | 66 | 62 | 80 | 140 | 98 | 77 | 7.29E-01 | 24.77 | 21.15 |
| South Africa | Kidneys | 2005 | 148 | 132 | 124 | 160 | 140 | 135 | 154 | 8.68E-01 | 11.84 | 11.03 |
| South Africa | Livers | 2005 | 14 | 22 | 36 | 20 | 37 | 36 | 30 | 4.38E-01 | 8.53 | 5.64 |
| Spain | Donors | 2003 | 1443 | 1495 | 1546 | 1509 | 1550 | 1577 | 1606 | 1.32E-01 | 50.48 | 18.32 |
| Spain | Kidneys | 2003 | 1991 | 2064 | 2113 | 2055 | 2074 | 2073 | 2093 | 4.94E-01 | 35.46 | 24.92 |
| Spain | Livers | 2003 | 1006 | 1022 | 1045 | 1033 | 1087 | 1080 | 1070 | 1.86E-01 | 28.53 | 12.31 |
| Sweden | Donors | 2000 | 97 | 108 | 98 | 114 | 123 | 128 | 137 | 9.18E-02 | 14.04 | 4.25 |
| Sweden | Kidneys | 2000 | 193 | 188 | 194 | 215 | 230 | 219 | 234 | 1.73E-01 | 17.36 | 7.22 |
| Sweden | Livers | 2000 | 104 | 95 | 97 | 122 | 124 | 132 | 121 | 3.52E-01 | 13.55 | 8.04 |
| Switzerland | Donors | 2000 | 98 | 95 | 75 | 95 | 91 | 92 | 80 | 8.40E-01 | 7.94 | 7.28 |
| Switzerland | Kidneys | 2000 | 180 | 156 | 131 | 167 | 163 | 178 | 159 | 8.41E-01 | 15.18 | 13.92 |
| Switzerland | Livers | 2000 | 77 | 82 | 73 | 93 | 80 | 80 | 86 | 8.59E-01 | 5.97 | 5.54 |
| Thailand | Donors | 2005 | 45 | 63 | 93 | 81 | 87 | 87 | 113 | 2.21E-01 | 20.21 | 9.51 |
| Thailand | Kidneys | 2005 | 20 | 114 | 164 | 149 | 155 | 166 | 202 | 1.69E-01 | 54.07 | 22.21 |
| Thailand | Livers | 2005 | 20 | 24 | 27 | 45 | 55 | 44 | 63 | 1.43E-01 | 15.21 | 5.76 |
| Tunisia | Donors | 2003 | 5 | 8 | 7 | 4 | 8 | 14 | 14 | 2.59E-01 | 3.70 | 1.88 |
| Tunisia | Kidneys | 2003 | 10 | 15 | 13 | 7 | 14 | 28 | 28 | 2.52E-01 | 7.72 | 3.88 |
| Tunisia | Livers | 2003 | 1 | 4 | 4 | 2 | 3 | 4 | 4 | 7.20E-01 | 1.12 | 0.95 |
| Turkey | Donors | 2003 | 105 | 136 | 153 | 143 | 223 | 262 | 262 | 8.84E-02 | 59.61 | 17.72 |
| Turkey | Kidneys | 2003 | 177 | 243 | 273 | 257 | 399 | 414 | 431 | 9.42E-02 | 92.31 | 28.33 |
| Turkey | Livers | 2003 | 85 | 113 | 124 | 114 | 209 | 212 | 229 | 1.21E-01 | 54.70 | 19.02 |
| United Kingdom | Donors | 2003 | 710 | 813 | 753 | 779 | 793 | 885 | 931 | 1.93E-01 | 70.48 | 31.00 |
| United Kingdom | Kidneys | 2003 | 1246 | 1905 | 1312 | 1396 | 1414 | 1382 | 1616 | 9.85E-01 | 207.70 | 206.13 |
| United Kingdom | Livers | 2003 | 630 | 721 | 597 | 637 | 627 | 683 | 660 | 9.31E-01 | 38.05 | 36.71 |
| United States of America | Donors | 2003 | 6455 | 7150 | 7593 | 8024 | 8089 | 7984 | 8021 | 1.30E-02 | 567.61 | 64.61 |
| United States of America | Kidneys | 2003 | 8664 | 9354 | 9914 | 10654 | 10587 | 10550 | 10442 | 2.25E-02 | 704.66 | 105.72 |
| United States of America | Livers | 2003 | 5351 | 5845 | 6121 | 6362 | 6227 | 6069 | 6111 | 7.89E-02 | 306.70 | 86.14 |
| Uruguay | Donors | 2007 | 60 | 63 | 65 | 49 | 68 | 54 | 58 | 9.38E-01 | 6.07 | 5.88 |
| Uruguay | Kidneys | 2007 | 96 | 114 | 123 | 90 | 128 | 96 | 104 | 8.80E-01 | 13.55 | 12.71 |
| Uruguay | Livers | 2007 | 3 | 3 | 10 | 12 | 24 | 17 | 23 | 1.58E-01 | 7.99 | 3.17 |
