# Supplementary material for: Analysis of official deceased organ donation data casts doubt on the credibility of China’s organ transplant reform
Source: BMC Med Ethics. 2019 Nov 14;20:79. doi: 10.1186/s12910-019-0406-6 (PMC6854896; doi:10.1186/s12910-019-0406-6)
Supplement: Supplementary file 4 — Additional file 4. Comments on statistical significance from the analysis of COTRS 2018 data. [file 12910_2019_406_MOESM4_ESM.docx]

# **Additional file 4. Comments on statistical significance from the analysis of COTRS 2018 data.**

In the paper we explained why we did not quote any measures of statistical significance, in particular P-values. The primary reason is that we are looking for models that fit *too* well to data. Quantifying the *expected* deviations from a model cannot be done from the data itself; we can only quantify the *observed* deviations. The best way to determine expected deviations is from subject matter knowledge of a rapidly growing organ donation and transplantation network (which is confounded by the possibly unique conditions of organ transplantation in China). That is why we compared the data to other countries’ data.

Now, the simplified model y = a.x^2^ allows a linear regression analysis by fitting the linear model y* = m.x, where y* is the square root of the data y. (Fitting instead y* = m.x + d yields a value for d of 2.477 which has a P-value of .128 which means that it is not statistically significant.) Fitting y* = m.x (with again the 2010 value removed) yields m=10.385 which has a 95% confidence interval of 10.130 to 10.641. Squaring m gives us an estimate for a in y = a.x^2^ of 107.85 (which closely matches the estimate 107.86 of a found directly) and a confidence interval of 102.6 to 113.2. Our best guess of an artificial model is now given by:

y = 108x^2^

Previously, we also did not quantify any confidence intervals because statistically analyzing a quadratic model is fraught with technical difficulties that can invalidate the analysis. Linear models however are not subject to those difficulties and so we were now able to present a confidence interval for the parameter a.

On the significance of the model appearing *too* simple, for example the optimal q being *too* close to an integer in y = a.x^q^, there are informal ad hoc ways of looking at it. For example, if the null hypothesis is that the optimal value of q is equally likely to be anywhere between the integers, then the chance of its estimate lying within ±.07 of an integer is 14%. However the chance of lying within ±.01 is 2%. Therefore the optimal value going from 2.07 (P=.14) to 2.01 (P=.02) has gone from not statistically significant to significant. This is the flavor of why indications of a very simple model are important.
