# Supplementary material for: Analysis of official deceased organ donation data casts doubt on the credibility of China’s organ transplant reform
Source: BMC Med Ethics. 2019 Nov 14;20:79. doi: 10.1186/s12910-019-0406-6 (PMC6854896; doi:10.1186/s12910-019-0406-6)
Supplement: Supplementary file 5 — Additional file 5. Results and Discussion of analysis of Red Cross and hospital-level data in five provinces. [file 12910_2019_406_MOESM5_ESM.docx]

# Additional file 5. Results and Discussion of analysis of Red Cross and hospital-level data in five provinces.

As noted in Methods, 5 regions (out of 28 where data was available) were chosen for case study analyses of their individual transplant center data reports compared to the provincial Red Cross reports. These regions are Henan Province, Guangxi Zhuang Autonomous Region, Shaanxi Province, Sichuan Province, and Zhejiang Province. They were selected based on a number of heuristics for the detection of problematic data, including: precise doubling of numbers, implausible organs/donor ratios, or reports of a large number of transplants in regions with relatively less developed healthcare infrastructure.

Following are the Results and Discussion of this analysis.

## Results

### Henan Province

Henan, a province of 100 million people in central China, has six transplant hospitals approved by the NHFPC. According to provincial Red Cross data, Henan’s voluntary transplant record is as follows:

**Table A. Red Cross Data of Voluntary Transplants in Henan Province.**

| **Cumulative totals since 1/1/2010** | | | **Rates in interval to reporting date** | | | |
| --- | --- | --- | --- | --- | --- | --- |
| **Date** | **Cumulative total of donors** | **Cumulative total of transplants** | **Months** | **Donors per month** | **Transplants per month** | **Organs per donor** |
| 4/1/2015 | 170 | 505 | 63.0 | 2.7 | 8.0 | 3.0 |
| 3/31/2016 | 341 | 975 | 12.0 | 14.3 | 39.2 | 2.7 |
| 10/15/2016 | 518 | 1492 | 6.5 | 27.4 | 80.0 | 2.9 |
| 11/10/2016 | 554 | 1638 | .9 | 42.2 | 171.3 | 4.1 |
| 3/31/2017 | 1108 | 2741 | 4.7 | 118.2 | 235.3 | 2.0 |

Full dataset available in Additional file 3.

Fig A displays Henan’s donor and transplant figures.


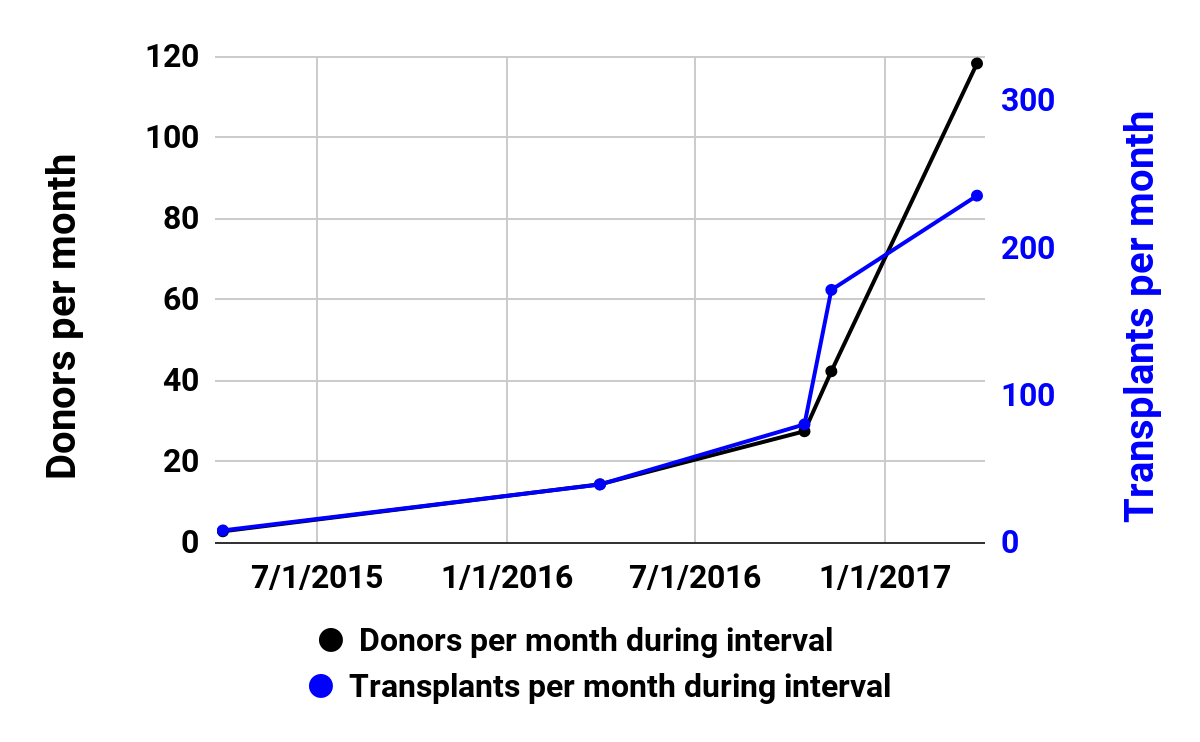


**Fig A. Henan Province Red Cross Transplant Data Since 1/1/2010.** Points on graph are values during the prior time interval. Scales set so that lines coincide when transplants/donor equals 2.75.

There are a number of peculiarities to observe about this dataset. These include:

1. The number of donors almost exactly doubles (plus one) between 4/1/2015 and 3/31/2016 (from 170 to 341);
2. The number of donors exactly doubles between 11/10/2016 and 3/31/2017 (from 554 to 1108) while in the same period the number of organs transplanted per donor is almost precisely 2 (1103/554=1.991);
3. The numbers of donors per month grows at a difficult-to-credit pace: from November 2016 to March 2017, Henan claims to have processed as many voluntary donors in four months as in nearly seven years prior, from the beginning of the pilot programs in 2010 until late 2016;
4. The number of transplants per month match this remarkable increase, but they do so inconsistently. For instance, for the 26-day period from 10/15/2016 to 11/10/2016, the average donor provided 4.1 organs, which is significantly greater than the rate in the central Red Cross data of 2.75. This indicates that every donor must on average have provided at least 2 kidneys, a liver, and either the heart or at least 1 lung, and that for every donor that donated only 3 organs, another donor must have provided 5 organs, on average. Yet in the period before and after this the rate of transplants per donor was 2.9 and 2.0 respectively.

The People’s Hospital of Zhengzhou and the Zhengzhou University First Affiliated Hospital are the most active transplant hospitals in Henan Province, and the only two hospitals that report their organ donation figures on their websites.

The People’s Hospital of Zhengzhou reports on its website that as of December 12, 2016, the hospital’s Organ Procurement Organization had performed transplants from a cumulative total of “nearly 100” volunteer donors (identified as “DCD transplants”) [[1]](https://paperpile.com/c/adxOfw/q9GZj). The same article notes that the hospital performed 60 liver and 210 kidney transplants in 2016 alone. This implies a minimum of 105 donors (i.e. two kidneys per donor) in 2016. Taken together, these two statements imply that the hospital performed almost no voluntary deceased transplants prior to 2016.

Zhengzhou University First Affiliated Hospital does not report its 2015 or 2016 voluntary deceased donor figures. Its website reports that it processed 51 cases of voluntary deceased donors in 2014, 16 at its own hospital and 35 from elsewhere. It also reports it performed 106 liver transplants and 230 kidney transplants in 2014, not noting they were voluntary and thus implicitly identifying them as sourced from prisoners [[2]](https://paperpile.com/c/adxOfw/99PTK).

Of the remaining four transplant centers, the First Affiliated Hospital of Henan Traditional Medical College, and the Zhengzhou Municipal No. 7 Hospital, provide no data on their websites about their OPO teams or voluntary transplants performed. The Jinan Military Region No. 153 Hospital does not have a website. Henan Provincial People’s Hospital says it has one staff member in its OPO, but provides no data about voluntary transplants performed [[3]](https://paperpile.com/c/adxOfw/2NvpI).

In sum, of the two hospitals known to be active in voluntary transplant work in Henan, the Zhengzhou University First Affiliated Hospital reported 51 voluntary deceased donors in 2014, and the People’s Hospital of Zhengzhou, reported 105 by mid-December 2016. Despite this, in early 2017 the provincial total voluntary deceased donors was reported as 1108.

### Guangxi Zhuang Autonomous Region

Guangxi, a large, relatively impoverished region in southwest China, has five NHFPC-authorized transplant hospitals. The region’s Red Cross statistics report that Guangxi hospitals performed the following number of transplants:

**Table B. Red Cross Data of Voluntary Transplants in Guangxi.**

| **Cumulative totals since 1/1/2010** | | | **Rates in interval to reporting date** | | | |
| --- | --- | --- | --- | --- | --- | --- |
| **Date** | **Donors** | **Transplants** | **Months** | **Donors per month** | **Transplants per month** | **Organs per donor** |
| 3/30/2015 | 339 | 894 | 63.0 | 5.4 | 14.2 | 2.6 |
| 3/29/2016 | 541 | 1399 | 12.0 | 16.8 | 42.1 | 2.5 |
| 12/31/2016 | 770 | 2511 | 9.1 | 25.2 | 122.2 | 4.8 |

Full dataset available in Additional file 3.

A problematic feature of this data is the claim of an average of 4.8 solid organs per donor from March to December 2016, a figure far higher than the national mean. Following is a table of the hospitals in the province and the transplants they have reported on their hospital websites.

**Table C. Hospital Website Data of Voluntary Transplants in Guangxi Hospitals.**

| **Cumulative totals since 1/1/2010 to end of 2016** | | |
| --- | --- | --- |
| **Hospital** | **Donors** | **Transplants** |
| People's Hospital of Guangxi Zhuang Autonomous Region | - | - |
| First Affiliated Hospital of Guangxi Medical University | 12^a^ | 32^a^ |
| Guangxi TCM College Affiliated Ruikang Hospital | - | - |
| Guangzhou Military Region No. 181 Hospital | - | - |
| Guangzhou Military Region No. 303 Hospital | 378^b^ | 1,000^b^ |

^a^ 遗体器官捐献 生命最后的馈赠. [Cadaver and Organ Donation: Life’s Final Gift]. First Affiliated Hospital of Guangxi Medical University. 2015 Dec 18. Chinese. Available: <http://www.gxmuyfy.com/gxmufy1/1fy/browse/browse6.asp?id=7711>; <http://archive.is/E5mO5> Cited 1 Oct 2017.
^b^ 303医院器官捐献首次单年突破百例大关. [303 Hospital Organ Donations Exceed the Major Barrier of 100 in One Year for the First Time]. PLA No. 303 Military Hospital Information Center. 2016 Nov 18. Available: [http://www.303yy.com/html/kswz/D30_1633_1105.html Cited 1 Oct 2017](http://www.303yy.com/html/kswz/D30_1633_1105.html%20Cited%201%20Oct%202017).

This indicates that the 303 Military Hospital had by far the most robust transplant center in the province. In 2013 the 303 Military Hospital was ranked third in the nation (in the kidney transplant registry) for voluntary donation activity [[4]](https://paperpile.com/c/adxOfw/FZOMJ), behind only the Sun Yat-sen First Affiliated Hospital in Guangdong (home base to Huang Jiefu) and the Zhejiang University School of Medicine’s First Affiliated Hospital (home base to Zheng Shusen, another high ranking transplant official), both of which are nationally renowned transplant centers. It was awarded a “national model transplant center” prize in March 2016, and carries out extensive engagement with foreign surgeons. Thus, it is far more advanced in transplant work than any other hospital in Guangxi and most hospitals in China.

According to the data above, the 303 Military Hospital processed almost half (378 of 770) of Guangxi’s reported donors prior to end of 2016 and about two fifths (1000 of 2511) of Guangxi’s reported transplants. Only one other hospital in the province reported having performed voluntary transplants, for a total of 12 donors.

When these two figures are added together (378+12=390), they come to about half of the claimed cumulative total of 770 donors in Guangxi. This leaves an unexplained 380 donors (770 - 378 - 12) and 1479 transplants (2511 - 1000 - 32) which have the high transplants/donor rate of 3.9 (1479 / 380).

### Shaanxi Province

Shaanxi is a province in northwest China with a population of 37 million. Four hospitals there are authorized by the NHFPC to perform organ donations. The region’s Red Cross statistics report that these hospitals have performed the number of transplants in Table D.

**Table D. Red Cross Data of Voluntary Transplants in Shaanxi.**

| **Cumulative totals since 1/1/2010** | | | **Rates in interval to reporting date** | | | |
| --- | --- | --- | --- | --- | --- | --- |
| **Date** | **Donors** | **Transplants** | **Months** | **Donors per month** | **Transplants per month** | **Transplants per donor** |
| 12/31/2014 | 67 | 167 | 60.0 | 1.1 | 2.78 | 2.5 |
| 3/25/2015 | 200 | 500 | 2.7 | 48.9 | 122.4 | 2.5 |
| 4/28/2016 | 334 | 900 | 13.1 | 10.2 | 30.5 | 3.0 |
| 7/18/2016 | 365 | 1000 | 2.7 | 11.7 | 37.7 | 3.2 |
| 12/31/2016 | 479 | 1320 | 5.4 | 21.0 | 59.0 | 2.8 |
| 7/17/2017 | 533 | 1400 | 6.5 | 8.3 | 12.3 | 1.5 |

Full dataset available in Additional file 3.

Table D shows that Shaanxi Red Cross reports initially processing an average of 1.1 donors per month between 2010 (when the program began) and 2014, until an abrupt increase to over one per day for the first three months of 2015. Following this period of high donations, there were two years of much more modest activity.

Table E shows the hospitals in the province and the transplants they report performing on their websites.

**Table E. Hospital Website Data of Voluntary Transplants in Shaanxi Hospitals.**

| **Cumulative totals since 1/1/2010 to April 2017** | | |
| --- | --- | --- |
| **Hospital** | **Donors** | **Transplants** |
| No.1 Affiliated Hospital of Xi'an Jiaotong University Medical School | 378^a^ | 958^a^ |
| Shaanxi People's Hospital | - | - |
| Xijing Hospital Affiliated with the Fourth Military Medical University | - | - |
| Shaanxi People’s Armed Police General Hospital | 6^b^ | - |

^a^ Chen D. 我院肾移植科党婉莹被评为2016年度全国优秀人体器官捐献协调员. [Dang Wanbao of the hospital’s kidney transplant ward has been chosen as 2016’s national outstanding organ transplant coordinator]. No.1 Affiliated Hospital of Xi'an Jiaotong University Medical School. 2017 Mar 31. Chinese. Available: <http://www.dyyy.xjtu.edu.cn/info/1779/21470.htm> Cited 1 Oct 2017.

^b^ Cardiovascular department. 公民死亡后器官捐献在我院已顺利开. [Citizen organ donation after death has begun smoothly at our hospital]. Shaanxi People’s Armed Police General Hospital. 2014 Nov 27. Chinese. Available: <http://www.wjyy029.com/NewsContent.aspx?cmscontentid=3845&categoryid=53>; <http://archive.is/oNix3> Cited 1 Oct 2017.

The totals from the four authorized transplant hospitals are significantly less than those claimed by the Red Cross. Moreover, much of the transplant activity at No. 1 Affiliated Hospital of Xi'an Jiaotong University Medical School took place within one year after the establishment of the hospital’s OPO in March 2016 [[5]](https://paperpile.com/c/adxOfw/yqLTq). This conflicts with the claim of the province having processed 365 donors by July 18, 2016.

### Sichuan Province

Sichuan, a province in southwest China, has four hospitals authorized to perform voluntary organ transplants. Table F shows Red Cross data for Sichuan province.

**Table F. Red Cross data of Voluntary Transplants in Sichuan.**

| **Cumulative totals since 1/1/2010** | | | **Rates in interval to reporting date** | | | |
| --- | --- | --- | --- | --- | --- | --- |
| **Date** | **Donors** | **Transplants** | **Months** | **Donors per month** | **Transplants per month** | **Organs per donor** |
| **12/25/2013** | 12 | - | - | - | - | - |
| **12/1/2014** | 20 | 62 | - | - | - | - |
| **1/12/2016** | 107 | 282 | 13.4 | 6.5 | 16.5 | 2.5 |
| **12/6/2016** | 235 | 500 | 10.8 | 11.9 | 20.2 | 1.7 |
| **3/10/2017** | 296 | 786 | 3.1 | 19.7 | 92.3 | 4.7 |
| **3/24/2017** | 304 | 811 | 0.5 | 17.4 | 54.5 | 3.1 |
| **4/1/2017** | 309 | 826 | 0.3 | 19.1 | 57.2 | 3.0 |

Full dataset available in Additional file 3.

The claim of 1.7 transplants/donor from 1/12/2016 to 12/6/2016 is unusually low; the claim of 4.7 from 12/6/2016 to 3/10/2017 is implausibly high. Reports on Sichuan hospital websites for transplant figures are shown in Table G.

**Table G. Hospital Website data of Voluntary Transplants in Sichuan.**

| **Cumulative totals since 1/1/2010 to April 2017** | | |
| --- | --- | --- |
| **Hospital** | **Donors** | **Transplants** |
| Sichuan University Huaxi Hospital | ~150^a^ | ~300^a^ |
| Sichuan Provincial People’s Hospital | - | - |
| Chengdu Military Region General Hospital | - | - |
| Chengdu Military Region No. 425 Hospital | - | - |

^a^ Huang ZL. 四川大学华西医院肾脏移植中心简介. [Introduction to the Sichuan University Huaxi Hospital kidney and liver transplant center]. Sichuan University Huaxi Hospital. 2017 Apr 20. Chinese. Available: <http://www.cd120.com/htmlzkjswaikexingkeshimini/66669.jhtml>. Note: These numbers are approximate minimums. The source reports 300 voluntary deceased kidney transplants by April 2017. If two kidneys were extracted from each deceased donor, which other data indicates is typical, the donors would be 150.

These figures are significantly less than claimed by provincial Red Cross authorities.

### Zhejiang Province

Zhejiang, a relatively wealthy coastal province, has eight NHFPC-authorized transplant hospitals. Table H is a selection from the provincial-level data collected for this study which can be found in Additional file 3. This data has been selected in order to more easily point out inconsistencies and anomalies.

**Table H. Selected Data of Transplants in Zhejiang Province.**

|  |  | **Zhejiang Red Cross Data** | | **Shulan Hospital** | **Zhejiang First Affiliated Hospital** |
| --- | --- | --- | --- | --- | --- |
|  | **Cumulative total to date^a^** | Donors | Transplants | Liver transplants | Liver transplants^b^ |
| **A** | **10/30/2014** | - | - | - | 1580^c^ |
| **B** | **1/1/2014 - 11/5/2014** | - | - | - | 149^c^ |
| **C** | **4/30/2016** | 472 | - | - | 1800^d^ |
| **D** | **5/30/2016** | 492 | 1400 | - | - |
| **E** | **6/28/2016** | 511 | 1530 | - | - |
| **F** | **1/1/2017 - 6/1/2017** | - | - | 100^e^ | - |
| **G** | **6/13/2017** | - | - | - | 2236^f^ |
| **H** | **7/6/2017** | 730 | - | - | - |

Full dataset available in Additional file 3.

^a^ All figures are full cumulative totals since transplants began (which for voluntary donation figures is 1/1/2010) except where indicated by the date.

^b^ Liver transplants include transplants from all sources.
^c^ Publicity Center. 中国肝移植注册中心落户浙大一院. [The China Liver Transplant Registry arrives at Zhejiang First Affiliated]. Zhejiang First Affiliated Hospital. 2014 Nov 6. Chinese. Available: <http://www.zy91.com/zxxw/2136.jhtml>
^d^ Publicity Center. 浙大一院开展全国首例绿色通道转运人体器官顺利运抵并成功移植. [Zhejiang First Affiliated is first in country to open green channel for organ transport, smoothly transporting and successfully completing transplant]. Zhejiang First Affiliated Hospital. 2016 May 8. Chinese. Available: <http://www.zy91.com/zxxw/3075.jhtml?127>
^e^树兰上半年工作超额完成 JCI动员号角已吹响. [Work at Shulan hospital in the first half of the year exceeded quotas; the bugle horn for JCI mobilization had sounded]. Shulan Hospital. 2017 July 20. Chinese. Available: <http://www.shulanhealth.com/3651.html>
^f^ Zhang M. 又游泳又长跑，想不到他是换肝人. [Swimming and running again, none would think he’s a liver transplant recipient]. Zhejiang News. 2017 June 13. Chinese. Available: <http://zj.zjol.com.cn/news/668637.html>. Note: Also reproduced on hospital’s website.

There are two items of interest in the table. The first is that data in rows D and E imply an unrealistic rate of 6.8 transplants/donor for all hospitals in Zhejiang between 5/30/2016 to 6/28/2016, calculated from the differences between the cumulative totals: (1530 - 1400) / (511 - 492).

The second is that the data in rows C and H shows the total number of voluntary deceased donors for all hospitals in Zhejiang in the period 4/30/2016 to 7/6/2017 to be 258 (730 - 472). All deceased donors from the beginning of 2015 should be volunteers. Yet in the slightly shorter subset of time from row C to G, spanning 4/30/2016 to 6/13/2017, the number of liver transplants in the First Affiliated Hospital alone is 436 (2236 - 1800), while row F shows that Shulan Hospital reports 100 in a still smaller subset of time. These two hospitals alone declare 278 (436 + 100 - 258) more donors than are accounted for in the Red Cross statistics.

## Discussion

In order to explore potential explanations for the anomalous qualities of the COTRS and central Red Cross datasets, five provinces were sampled. Provincial Red Cross data was compared to hospital activity in an attempt to ascertain whether indications of data falsification and manipulation extended down to the provincial level.

There are two parts to the analysis. The first is a test of the internal coherence and integrity of the provincial Red Cross figures. Metrics such as transplants/month and transplants/donor reveal whether the figures are consistent with data generated by actual donation and transplantation activity. If the data shows highly unusual or artificial patterns, ratios, or other disqualifying anomalies, then the contention that provincial-level Red Cross data is manufactured or manipulated to sustain central quotas is supported.

The second part to the analysis is an examination of hospital activity. Unlike a finding of contaminated provincial Red Cross data, however, examining hospital activity is unable to confirm a hypothesis of data falsification, because there is no way to guarantee that a hospital that has not reported transplants nonetheless performed transplants.

Nevertheless, the absence of congruence between hospital activity and local Red Cross data is consistent with data manipulation, especially if provincial Red Cross data fails basic tests of data integrity

The Red Cross data in five regions — Henan, Guangxi, Shaanxi, Sichuan, and Zhejiang — was compared with hospital activity in those regions. They were incongruent in each case. In several cases the Red Cross figures failed basic tests of data integrity. In four cases the number of claimed transplants were unaccountably larger, sometimes much larger, than transplant figures reported at the constituent hospitals. In Zhejiang, 278 more liver transplants took place at the province’s hospitals than there were donors reported by the provincial Red Cross, indicating the use of nonvoluntary donors. All findings are predicted by a hypothesis of systematic data falsification.

#### Henan Province

In Henan, the Red Cross reported that 1108 donors provided 2741 organs by end March 2017. Yet the two busiest transplant hospitals in the province reported only 156 donors by end of 2016 — around 13% of the stated total. Henan’s Red Cross figures also exhibited anomalous data features, including the claim of 4.1 transplants/donor for a single month.

#### Guangxi Zhuang Autonomous Region

In Guangxi, the Red Cross reported 770 cumulative donors providing 2511 transplanted organs by end 2016. Reported hospital activity showed only 390 sources and 1032 organs respectively — or around half the stated total of 770 donors and 2511 transplants by end 2016. For nine months in 2016, the Red Cross figures claimed 4.8 solid organs transplanted per donor — an extremely high number. These two findings show that the provincial Red Cross claims are not supported by data of reported hospital activity and that the provincial Red Cross data is incoherent.

#### Shaanxi Province

In Shaanxi, the Red Cross reported 479 donors supplying 1320 organs by end 2016. Reported hospital activity showed only 378 donors by April 2017. Moreover, the data on donor rates is suspicious, with 48.9 donors per month reported for the first three months of the program in the beginning of 2015. This is followed by a far more modest claim of about 10 donors per month. It is difficult to imagine why so many donors from natural deaths would suddenly emerge in the first three months of 2015, followed by a significant drop for the next two years. Common sense would suggest the opposite growth trajectory, as the new system is being built.

Shaanxi’s transplants/donor figures are within the bounds of plausibility, though 3.2 for three months in 2016 is relatively high, and an average of 1.5 for the first nearly seven months of 2017 is unusually low. There is no particular reason for transplants/donor to halve as time goes on, and as doctors and transplant coordinators gain more experience, one would expect that organ recovery rates would in fact increase. While these figures are not impossible, they are implausible.

To put the figures in context: Shaanxi, a poor province with only one major transplant hospital, is claiming to be nearly twice as industrious as Shanghai, despite the latter having 11 advanced transplant hospitals and being at the cutting edge of transplant medicine for several decades. For these reasons the figures do not appear to represent genuine transplant activity.

#### Sichuan Province

Sichuan’s Red Cross data is compromised in the same fashion as other provinces, with a claim of a very low 1.7 transplants/donor for a period of nearly 11 months, followed by the highly unlikely 4.7 transplants/donor from 60 donors over 3 months. Sichuan’s Red Cross officials claim 309 donors providing 826 organs by April 2017, but hospital website reports support only around 150 donors providing 300 organs by that date.

#### Zhejiang Province

Zhejiang presents a different inconsistency than that found in other provinces. The Red Cross figures are internally consistent except for a single anomaly — an implied transplants per donor rate of 6.8 over a two month period — but the transplant figures reported on hospital websites, rather than failing to match Red Cross numbers, instead exceed them by a significant margin.

Between 4/30/2016 and 6/13/2017, hospitals in Zhejiang performed 278 more liver transplants than there were voluntary donors accounted for by the provincial Red Cross. If living donors were assumed to compose 12.5% of this total (per the 2016 national rate, of 406 living liver donors versus 3257 deceased liver donors, according to COTRS 2017 data) the number of deceased donors of unidentified origin would be 243. These livers are in excess of the voluntary deceased donors, and are primarily attributable to Zhejiang University School of Medicine’s First Affiliated Hospital. The only possible source of nonvoluntary deceased donor livers are prisoners of one kind or another.

The case of Zhejiang supports the hypothesis that provincial transplant data is unrepresentative of actual transplant activity in constituent hospitals, this time from a different direction. Zhejiang underreported its transplants, rather than overstated them.

#### Local hospital data study limitations

The methods by which these findings were reached were necessarily limited. Hospitals in China are not required to publicly report their transplant figures. There is no open, central database, as in advanced countries with voluntary donation systems, that the public and researchers may inspect. The lack of transparency around this seemingly innocuous data hampers the ability to make definitive conclusions.

Yet this is also a key finding of the study: the unverifiable nature of China’s reports of extraordinary achievements with respect to voluntary transplant reform is itself a concern.

While the absence of some data disables conclusive statements, there are several considerations supporting the value of the findings from hospital-level analysis:

1. In general, voluntary organ transplant work has been highlighted as a political and propaganda priority by the Communist Party, and state media agencies regularly promote stories about successful voluntary transplants and the hospitals performing them [[6, 7]](https://paperpile.com/c/adxOfw/AisFZ+o5fTV). The incentives thus point in the direction of more reporting of voluntary transplants rather than less. Unlike the era when it was widely known that organ transplants in China were unethically sourced, in which case hospitals were more circumspect with their public statements, since 2015 every hospital can claim that its transplants have been voluntary. It is thus reasonable to expect that transplants reported would roughly correspond with, if not overstate, transplants performed;
2. In each province, the medical publications of each hospital were examined and found to be generally consonant with the reports of transplants on hospital websites;
3. In each province there were only one or two hospitals that performed the majority of transplants. Typically, these are run by surgeons of national repute. There are no other indications that the other, lesser-known hospitals also maintain such significant donor programs, which in most cases would be required to compensate for the missing figures. The fact that OPOs are run by hospital transplant centers, rather than being independent, geographically-based NGOs, also suggests that in any region there will be a competition for scarce organs, and the major centers will gain a greater market share. Further, leading Chinese medical administrators have stated that the majority of organ donation activity takes place in a minority of hospitals, which is consistent with the intra-provincial analysis.

The examination of hospital-level transplant activity fails to disconfirm the data-based findings, but rather tends to corroborate them. While this finding cannot be conclusive due to the lack of transparency around hospital activity, we believe that the most plausible interpretation of the data is that it is part of a pattern of data fabrication extending to the provincial level.

# References

[1. 器官移植中心：14年“跬步”积累成就今日“千里”质变 [Organ transplant center: 14 years of “steps” formed the “thousand miles” of success today]. 2016.](http://paperpile.com/b/adxOfw/q9GZj) <http://www.zzrmyy.com/art_news_4266.aspx>[.](http://paperpile.com/b/adxOfw/q9GZj)

[2. Gou ZJ, Chen F. 扎实做好人体器官移植捐献工作. [Grasp well the task of human organ donation work]. First Affiliated Hospital of Zhengzhou University Propaganda Office. 2015.](http://paperpile.com/b/adxOfw/99PTK) <http://fcc.zzu.edu.cn/newsss/vmsgisapi.dll/onemsg?msgid=1505061625479626698.> [Accessed 2 Nov 2017.](http://paperpile.com/b/adxOfw/99PTK)

[3. 关于医院内设机构副职及下设科室中层干部竞聘上岗的通知 [Notice regarding the establishment of auxiliary and mid-level administrative cadre hospital posts]. 2017.](http://paperpile.com/b/adxOfw/2NvpI) <http://www.hnsrmyy.net/OfficeNewsDetail-8175.html>[.](http://paperpile.com/b/adxOfw/2NvpI)

[4. 特色技术. [Special Techniques]. PLA No. 303 Military Hospital Information Center. 2014.](http://paperpile.com/b/adxOfw/FZOMJ) <http://www.303yy.com/html/kswz/T30_1629_721.html.> [Accessed 2 Dec 2018.](http://paperpile.com/b/adxOfw/FZOMJ)

[5. Chen D. 我院肾移植科党婉莹被评为2016年度全国优秀人体器官捐献协调员. [Dang Wanbao of the hospital’s kidney transplant ward has been chosen as 2016’s national outstanding organ transplant coordinator]. No.1 Affiliated Hospital of Xi’an Jiaotong University Medical School. 2017.](http://paperpile.com/b/adxOfw/yqLTq) <http://www.dyyy.xjtu.edu.cn/info/1779/21470.htm.> [Accessed 1 Oct 2017.](http://paperpile.com/b/adxOfw/yqLTq)

[6. Huang JF. 中国器官捐献的发展历程与展望. [The History and Outlook of Organ Donation in China]. Medical Journal of Wuhan University. 2016;37:517–22.](http://paperpile.com/b/adxOfw/AisFZ)

[7. 马奔同志到中国人体器官捐献管理中心调研 [Comrade Ma Ben visits and inspects the China Organ Donation Administration Center]. 2017.](http://paperpile.com/b/adxOfw/o5fTV) <http://www.nhfpc.gov.cn/jcj/lzgj/201707/a9b84e1ebd214f3d8f7a99795e64c72a.shtml>[.](http://paperpile.com/b/adxOfw/o5fTV)
