# Supplementary material for: Analysis of official deceased organ donation data casts doubt on the credibility of China’s organ transplant reform
Source: BMC Med Ethics. 2019 Nov 14;20:79. doi: 10.1186/s12910-019-0406-6 (PMC6854896; doi:10.1186/s12910-019-0406-6)
Supplement: Supplementary file 6 — Additional file 6. Additional sources and considerations regarding China’s deceased organ donation system. [file 12910_2019_406_MOESM6_ESM.docx]

# Additional file 6. Additional sources and considerations regarding China’s deceased organ donation system.

Following is an expanded discussion of related considerations regarding the Chinese authorities’ challenges in developing a national, hospital-based voluntary deceased donor system. These factors are important for evaluating the overall credibility of the data and in particular the claims of voluntary organ donations growing at such a rapid rate.

## Data transparency and availability

China has always been secretive about its organ transplant program. The lack of transparency is illustrated in Table A, compiled based on information found in official reports and medical publications about extant datasets, what they report, their custodianship, and their public status.

**Table A. Chinese Government Sources of Transplant Data.**

| **Dataset** | **Custodianship** | **Background** | **Public status** |
| --- | --- | --- | --- |
| National Health and Family Planning Commission (NHFPC, formerly the Ministry of Health) internal data on organ transplants | NHFPC, Beijing | Data collected since organ transplants began in China, obtained from hospitals around the country | Closed |
| China Organ Transplant Response System (COTRS)^a^ | China Organ Transplantation Development Foundation, Beijing | The sole legal allocation system for organ transplants since September 1, 2013 | Available for collection^b^ |
| China Organ Donation Administrative Center | Red Cross Society of China, Beijing | Established in April 2014. Parallel (and nominally identical) to COTRS | Available for collection^b^ |
| China Liver Transplant Registry | Zhejiang University First Affiliated Hospital, Hangzhou^c^ | Established in 2005 | Closed |
| China Lung Transplant Registry | Wuxi Provincial People’s Hospital, Wuxi^d^ | Established in 2010 | Closed |
| China Heart Transplant Registry | Fuwai Cardiovascular Hospital, Beijing^e^ | Established in 2010 | Closed |
| China Scientific Registry of Kidney Transplants | 309 PLA Military Hospital, Beijing | Established in 2009 by then Ministry of Health^f^ | Closed |
| Transplant Patient Management System | 309 PLA Military Hospital, Beijing | Established with Shanghai Roche Pharmaceuticals Ltd. in 2000^g^ | Closed |
| Hospital Information Systems at every hospital in China | Each hospital that has performed transplant surgeries | Data collected since organ transplants began in China. Each of the over 1000 hospitals that have performed transplant operations will have this data | Closed |
| Immunosuppressant drug health insurance reimbursement database | NHFPC, Beijing | Data collected since reimbursement for immunosuppressant drugs began in early 2000s | Closed |
| Local Red Cross figures | Red Cross Society of China, Beijing | Reported in local officially-controlled media or on local Red Cross websites | Available for collection^b^ |
| Chinese Society of Organ Transplantation figures | Chinese Society of Organ Transplantation, Beijing | Data collected since at least 2000 and likely earlier | Closed |

^a^ China Organ Transplant Response System [Internet]. Beijing: National Health and Family Planning Commission; c2017 [cited 2017 Oct 1]. Available: <https://www.cot.org.cn/>
^b^ Refers to data that does not exist as a complete, official dataset in a single place — it must be manually collected and archived from official websites or presentations, making it a time-consuming research procedure.
^c^ Zhao H, Li Z. 中国器官移植发展基金会：深化器官移植 落实“依法治国”. [China Organ Transplantation Development Foundation: Deepen the implementation of ‘rule of law’ in organ transplantation]. CN-Healthcare.com 2015 Oct 14. Chinese. Available: <http://www.cn-healthcare.com/article/20150203/content-469830.html>
^d^ 肺移植诊疗中心简介. [Introduction to the lung transplantation diagnosis and treatment center]. Wuxi People’s Hospital. [2015?]. Chinese. Available: <http://www.wuxiph.com/KeShi/WaiKeJiTong/188.html>
^e^ 个人简介. [Biography of Huang Jie] [Internet]. Fuwai Hospital, Chinese Academy of Medical Sciences. [Date unknown]. Chinese. Available: <http://www.fuwaihospital.org/Hospitals/Doctors/Detail/2253>
^f^  Zhang L, Zhang J, Lin J, Tian Y. 中国肾移植科学登记系统对青年医师的培训应用体会. [Experience of training for young doctors by Chinese scientific registry of kidney transplantation]. China Medical Herald. 2013 Apr;10(12):148-152; Chen LP, Shi BY. 中国肾移植科学登记系统（CSRKT）发展历程. [The history of the development of the China Scientific Registry of Kidney Transplants]. NHFPC Kidney and Liver Transplant Quality Control Center (People’s Liberation Army No. 309 Hospital). [2016?]. Chinese. Available: http://www.csrkt.org/main/history.do. Accessed 6/15/2017
^g^ 中国肾移植科学登记系统. [China Scientific Registry of Kidney Transplants]. People’s Liberation Army No. 2 Affiliated General Hospital. 2009 Nov 25. Available: <http://web.archive.org/web/20091125112308/http://csrkt.org/> Cited 5 Apr 2017.

None of these 12 data sources are fully open to public scrutiny. The three datasets that are not closed are the primary sources of the data analyzed in this assay.

In some cases, the data from closed registries is reported in medical papers or cited in news reports, and researchers can gain some insight into official figures by a close reading of these sources.

The China Organ Transplantation Development Foundation, which manages COTRS, has been described by some Western observers as independent of the Chinese government [[1]](https://paperpile.com/c/YwsriB/OqiOR). Huang Jiefu and Chinese authorities have made clear that they do not understand this to be the case: It is under the supervision and management of the NHFPC [[2]](https://paperpile.com/c/YwsriB/ZY89K); it appears on the NHFPC’s website as an affiliated department [[3]](https://paperpile.com/c/YwsriB/QAzzm); it is funded by wealthy businessmen with close ties to Chinese officialdom or who hold official posts [[4, 5]](https://paperpile.com/c/YwsriB/6azvc+XkYOj) and it is headed by Huang Jiefu, a ministerial-ranking Chinese Communist Party official [[6]](https://paperpile.com/c/YwsriB/XXncR). For these reasons, the Foundation is best understood as an arm of the Chinese government and Communist Party.

Other parts of China’s transplant system continue to be heavily influenced and controlled by political actors. The Red Cross’s China Organ Donation Administrative Center (CODAC) is headed by Dr. Shen Zhongyang, whose appointment was made through the Organization Department of the Chinese Communist Party [[7]](https://paperpile.com/c/YwsriB/UcpqU). Shen is founder of the transplant program of the Tianjin First Central Hospital, the most well-known site of transplant tourism in China [[8]](https://paperpile.com/c/YwsriB/PABN9), and director of the transplant unit at the General Hospital of the People’s Armed Police. Shen is among the many military, paramilitary, and other senior transplant surgeons on the National Organ Donation and Transplantation Committee, the steering body that oversees China’s transplant reforms. Many of these surgeons, including Zheng Shusen, Shi Bingyi, Liu Yongfeng, Zhu Jiye and others, performed hundreds, and in some cases thousands, of transplants during the period when China sourced organs almost exclusively from prisoners.

Mainland Chinese entities took further control over the organ registry systems prior to the announcement of the cessation of prisoner donors. Custodianship over COTRS and the China Liver Transplant Registry was transferred from the University of Hong Kong — where they had been stationed “to ensure fair organ allocation and transparency” [[9]](https://paperpile.com/c/YwsriB/KUYYl) and because the university was considered “a neutral party to audit, manage and analyze the data”[[10]](https://paperpile.com/c/YwsriB/0qKMr) — to two major Chinese hospitals in 2014 [[11]](https://paperpile.com/c/YwsriB/4HIc0).

In response to questions by Chinese journalists, Dr. Huang Jiefu has referred to transplant data as a “state secret.”[[12]](https://paperpile.com/c/YwsriB/mIsG)

### Absence of Beijing Red Cross data

Red Cross reports having 1151 transplant coordinators in 28 regions [[13]](https://paperpile.com/c/YwsriB/7qlSX). Qinghai, Ningxia, and Tibet do not have transplant programs, and Heilongjiang, a province in northeast China, has performed few voluntary transplants [[14]](https://paperpile.com/c/YwsriB/biBYJ).

The most notable absence from the provincial Red Cross dataset is Beijing. This is highly unusual. Beijing has 20 transplant hospitals, the most in the country, and they are among the most advanced in both medical care generally and organ transplantation specifically. It is clear from clinical papers, hospital websites, and television documentaries that voluntary (and likely nonvoluntary) transplant work is indeed taking place in Beijing. Yet Beijing’s municipal Red Cross does not state how many transplants are performed in the city. Thus, of all the regions in the country, the capital is the only one not to have made its Red Cross data available at the time of this writing. This is contrary to the repeated assurances of transparency by Chinese officials.

## No system of required potential donors' referral

Potential donors' referrals are the lifeblood of any functioning voluntary organ donation and allocation system. Only a portion of referred imminent deaths will end up being eligible for donation, with a portion of this cohort providing consent to donate, a portion of that cohort actually becoming donors, and portion again of that cohort resulting in successful transplantation. In the United States and many other countries, it is mandated that hospitals report most imminent deaths to their regional OPO [[15]](https://paperpile.com/c/YwsriB/ubGG1).

The absence of such a regulation in China has been a source of tension since April 2014, when the deputy director of the Red Cross’s Organ Donation Administration Center, Gao Xinpu, remarked that the biggest issue China has is not signing up designated donors, but “finding potential donors in hospitals.”[[16]](https://paperpile.com/c/YwsriB/dQ4gG) As of this writing, there remains no policy on the matter, and hospital-based OPOs and Red Cross coordinators must still marshal their own resources to locate eligible donors. One outcome of this is that most organs are sourced from the intensive care units (ICUs) of transplant hospitals themselves, where the OPOs are based [[17]](https://paperpile.com/c/YwsriB/JaHOw). There are around 20000 hospitals in China; only 173 of them have OPOs and are authorized to perform transplants.

## A lack of coordination between bureaucracies and hospitals

There are at least two broad forms of cooperation that China’s transplant system must bring into effect: that between national bureaucracies and that between local hospitals and personnel.

On the former, Huang Jiefu has been candid in his disappointment when discussing the matter in Chinese sources. Regarding cooperation between the NHFPC and the Red Cross, he told state media in November 2015: “To this day we have not held a single meeting.”[[18]](https://paperpile.com/c/YwsriB/8damK) The Beijing Youth Daily, an official newspaper, summarized: “As chairman of the committee, he’s completely helpless and very anxious.” Huang was quoted saying: “It always comes back to departmental interests, bureaucratism, and the passivity and ineffectiveness of the administrative layers in the official system.”[[18]](https://paperpile.com/c/YwsriB/8damK) The two agencies were supposed to have begun working together in March 2014 when they were united under a single committee [[19]](https://paperpile.com/c/YwsriB/9MdBH).

At the local hospital level, challenges relate to coordination between surgical teams and appropriate care of the donor body. These issues have been the source of complaints by Chen Jingyu, China’s leading lung transplant surgeon. China’s organ donation system is “in its preliminary stage, with so many potential donors lacking proper care, leading to failed donations or poor quality organs,” he wrote in a July 2016 medical paper [[20]](https://paperpile.com/c/YwsriB/ROZWL).

In March 2015 the Anzhen Hospital in Beijing’s website included the following line: “Only 1-2% of donors can have hearts recovered for transplant… there is a massive amount of waste. The biggest contributor to this situation is lack of coordination and communication between healthcare institutions.”[[21]](https://paperpile.com/c/YwsriB/HQ4Hs) The hospital-based character of OPOs — which is not accompanied by clearly delineated Donation Service Areas — has also led to competition for the limited organs available [[22]](https://paperpile.com/c/YwsriB/zgNPt).

## Unauthorized transplants

An unknown number of hospitals have reported performing transplants that they are unauthorized to do, based on the NHFPC’s official list. A small sampling of cases follows.

- The Shenyang Military Region General Hospital is not authorized to perform liver transplants; it published a paper in June 2015 reporting 11 deceased liver transplants [[23]](https://paperpile.com/c/YwsriB/zHmp2).
- The First Affiliated Hospital of Medical University is not authorized to perform heart transplants; on July 13, 2016, its Communist Party Committee issued a statement reporting the second deceased heart transplant performed at the hospital [[24]](https://paperpile.com/c/YwsriB/xJ5jU).
- The Ningbo Municipal Yinzhou No. 2 Hospital in Jiangsu Province is not authorized to perform liver transplants; on December 6, 2016 the hospital performed a liver transplant [[25]](https://paperpile.com/c/YwsriB/Pc5I3).
- The First Affiliated Hospital of Jilin University is not authorized to perform heart or lung transplants; between January 2013 and December 2015, it reports performing three heart and five lung transplants [[26]](https://paperpile.com/c/YwsriB/efwSH).

There is no indication in these and other cases that grave ethical violations are taking place, as long as the organs were procured from voluntary donors. However, according to official statements the breach of regulations (whether such regulations are well-considered or not) is subject to sanction, and there are no known reports of sanctions in these cases. The hospitals report having performed these unauthorized transplants on their websites.

## Possible misclassification of donors and continued use of prisoner donors

While the data manipulation so far explored relates largely to boosting the statistics of voluntary donors, there also appear to be instances of reclassification of nonvoluntary donors as “volunteers.”

This inference is made based on the following factors: prisoners were almost the sole source of organs in China until 2015; Chinese authorities have a history of vowing the cessation of this activity but persisting in it; there is no law or regulation criminalizing this activity, and no known sanctions of surgeons for engaging in it; the quasi-legal basis for this practice, the 1984 Temporary Rules, has not been rescinded; prisoner organs have been allocated via COTRS before, and high ranking Chinese officials have advocated this practice; apart from voluntary donors, nonvoluntary, prisoner donors are one of the few other possible source of deceased donor organs. (Eligible, nonconsenting, non-prisoner, hospital-based donors are another potential source, but we believe this pool would likely be low, for the reasons discussed in this section.) Examples follow.

### Zhejiang University School of Medicine’s First Affiliated Hospital

In October 2016, Liver International published a paper, co-authored by 17 Chinese transplant doctors, about fatty liver transplants at the Zhejiang University School of Medicine’s First Affiliated Hospital. The authors claimed that of the 564 liver transplants performed at the hospital between April 2010 and October 2014, all were voluntary — i.e. none were from prisoners [[30]](https://paperpile.com/c/YwsriB/5Bt3V). This claim was challenged by Wendy Rogers, Maria Fiatarone Singh and Jacob Lavee, who showed that China’s pilot program only began in 2010, and that the claim that 564 liver transplants at the hospital were all from voluntary deceased donors contradicted numerous other official pronouncements [[31]](https://paperpile.com/c/YwsriB/j6Is3). The corresponding authors — Zheng Shusen, a leading liver transplant surgeon and president of the hospital where the liver transplants took place, and his colleague Yan Sheng — issued a rebuttal to these arguments [[32]](https://paperpile.com/c/YwsriB/nIoos). Rogers et. al made a response showing that Zheng and Yan relied on misleading references and unsubstantiated assertions that undermined their own claims [[33]](https://paperpile.com/c/YwsriB/2Wuxc).

This response led to the retraction of the paper and a lifetime embargo of the authors from publication in Liver International [[34]](https://paperpile.com/c/YwsriB/VOhBZ). After their paper was retracted, the authors refused Liver International the right to publish their response to the initial complaint; the journal’s editors instead offered a summary.

Later, Huang Jiefu was quoted in state-affiliated media reprimanding Zheng for “falsifying data.” Huang said that the First Affiliated Hospital had only procured livers from 166 voluntary donors during the period in question, not 564 as claimed. Zheng in turn blamed the episode — which he had defended in written correspondence — on a junior colleague [[35]](https://paperpile.com/c/YwsriB/MT1cT). Huang’s statement indicates that Zheng Shusen and 16 colleagues falsely classified 398 nonvoluntary donors as voluntary.

While care must be taken not to make sweeping generalizations about an entire country’s transplant surgeons and medical authorities based on a single instance, there are a number of important considerations related to this case.

The first is that Zheng Shusen is one of China’s most well-known and prolific surgeons and transplant leaders. He is the president of the First Affiliated Hospital of Zhejiang University’s School of Medicine, a leading transplant hospital, and as the director of the transplant department has performed at least 2000 liver transplants (the majority from nonvoluntary donors) [[36]](https://paperpile.com/c/YwsriB/krOPM). He is the editor-in-chief of China’s preeminent medical journal on organ transplantation, the Chinese Journal of Organ Transplantation [[37]](https://paperpile.com/c/YwsriB/b3jGw). He is an Academician with the prestigious Chinese Academy of Engineering, the deputy director of the Chinese Medical Association, and president of China’s Organ Transplant Doctors Association [[38]](https://paperpile.com/c/YwsriB/TTkuX). His hospital is custodian to the China Liver Transplant Registry, which is supposed to have logged every liver transplant performed in China since 1980 [85]. He is also given the honor of leading the opening ceremonies of annual organ transplant events and conferences in China [[39]](https://paperpile.com/c/YwsriB/XnzqO).

After misclassifying 398 donors in a paper for an international medical journal, then persisting in the claim after being challenged, then shifting the blame to a junior colleague, Zheng was not subject to any professional or official sanction. None of his prestigious titles were stripped from him, nor was his profile in China’s transplant sector diminished. He presided over the China Organ Donation and Transplantation Conference in Kunming, Yunnan Province, in August 2017, leading the opening ceremony and moderating several panels [[38]](https://paperpile.com/c/YwsriB/TTkuX). This suggests that dubious scientific practice and misclassification of nonvoluntary donors carries few or no consequences.

At a national liver transplant conference in Sichuan in November 2015, Zheng presented data showing that around 10% of livers were sourced from prisoners that year (compared to 56% in 2014), contradicting the no-prisoner promises of Huang Jiefu [[40]](https://paperpile.com/c/YwsriB/lOPqA).

Zheng Shusen’s First Affiliated Hospital is also the majority contributor to the 278 liver transplants in Zhejiang Province between 4/30/2016 and 6/13/2017 that exceed the number of voluntary deceased donors in the province as reported by the provincial Red Cross.

### Wuxi People’s Hospital

Chen Jingyu, based at the Wuxi People’s Hospital in Jiangsu Province, is well known both in China and internationally for being almost the sole lung transplant surgeon in China. Chen reports performing lung transplants from 104 donors in 2014, 106 in 2015 (82 of which were dual) [[41]](https://paperpile.com/c/YwsriB/AOXmq), and 136 in 2016 [[42]](https://paperpile.com/c/YwsriB/Gw9JW). The claim that he managed to perform slightly more hospital-based transplants in 2015 over 2014, when the new policy banning the use of executed prisoners was put in place, appears to be inconsistent with other information.

According to the Wuxi municipal Red Cross, by end 2014 only 10 volunteers had donated organs in the city [[43]](https://paperpile.com/c/YwsriB/V2snh). The first lung transplant from a brain-dead patient in 2015 took place in early March of that year at Wuxi People’s Hospital [[44]](https://paperpile.com/c/YwsriB/2ZLNS). Wuxi Daily, the official publication of the city’s Communist Party Committee, reported that as of January 2017, the city had procured 94 organs from 29 voluntary donors in the five years since organ donation trial began in 2011 [[45]](https://paperpile.com/c/YwsriB/QKVKf). Given that Chen Jingyu does not state how many of his donors in 2015 and 2016 were local and how many from hospitals outside the region, and making the generous assumption that each of the donors in Wuxi provided lungs (whether one or both), this would indicate that 213 (106 + 136 - 29) donors in 2015 and 2016 must have been from outside Wuxi. Chen has publicly highlighted the immense difficulties in transporting allocated organs in China. [[46]](https://paperpile.com/c/YwsriB/YYzYc) All of these factors raise questions about the credibility of the claim of switching sourcing from nearly all prisoner donors to solely volunteer, hospital-based donors between 2014 and 2015.

### Organ Transplant Center at the Qingdao University Affiliated Hospital

An organ transplant center was established in the Qingdao University Affiliated Hospital in February 2014 with an ambitious plan to perform 100 organ transplant surgeries by the end of the year. The lead surgeon, Zang Yunjin, is one of China’s most industrious liver transplant surgeons, having performed 1,500 liver transplants over his career and serving as deputy director of the liver transplant center at the Beijing Armed Police General Hospital [[48]](https://paperpile.com/c/YwsriB/l2Y8y).

By August 2014 the center reported performing 30 liver transplants and 38 kidney transplants, including 6 surgeries within a 24-hour period. It reported that 10 of these were from voluntary donors [[49]](https://paperpile.com/c/YwsriB/foybi). By February 24, 2016, the hospital reported having performed 131 liver transplants and 193 kidney transplants; 72 voluntary deceased donors were recorded in 2015 [[50]](https://paperpile.com/c/YwsriB/7LgDL). In March 2016 Zang Yunjin had performed 1,800 liver transplants (up from 1,500 two years previously), according to the hospital’s website.

From this it can be inferred that from February 2014 to February 2016, the center performed 131 liver transplants, 82 of which were from voluntary deceased donors (10 in 2014, at most 72 in 2015), 49 of which were from unidentified donors. There is also the claim that Zang Yunjin performed 300 liver transplants in the two-year period, which, assuming he performed all 131 above, would leave another 169 livers sourced from unidentified donors. We believe that prisoners are the most plausible explanation for most of these over-200 otherwise unidentified deceased sources.

### General remarks on the apparent misclassifications

In the cases above, the number of donors that appear to have been misclassified prisoners far exceeded — by up to 7 times — the number of donors known to be voluntary. Each of these cases was discovered serendipitously. There is no systematic means of assessing the entire country for this conduct and determining whether the cases are representative or merely indicate of individual, rogue behavior.

The individuals in each case, however, are among the most prestigious and influential transplant surgeons in China. There are no reports of any of these doctors being investigated or sanctioned for what appears to be at least unethical scientific conduct.

These activities call into question official promises about the cessation of use of prisoners as donors. The phenomena also raise questions about organ sources in the cases where hospitals report 8 transplants within 12 hours [[51]](https://paperpile.com/c/YwsriB/rCv9q), 14 transplants within 48 hours [[52]](https://paperpile.com/c/YwsriB/KGyIp), or the ability to locate a donor for a recipient within 24 hours. Given that misclassification of donors can take place on a significant scale with no apparent consequences, questions naturally arise about the sources of the organ in these cases, each of which would require a series of fortunate coincidences if the donors were voluntary.

## Willingness to donate

All hospital-based deceased organ donations in China, according to official statements, require consent (opt-in) from family [[53]](https://paperpile.com/c/YwsriB/bhfp6). Yet the question of organ donation in China remains a vexing cultural issue, and there is no indication that traditional attitudes on donation have changed dramatically in the last few years, despite attempts to promote the practice in state media reports. Surveys from Chinese hospitals illustrate current trends.

A study of 67 eligible donors between March 2010 and December 2011 at the Tianjin First Central Hospital found that 16 went on to donate, a success rate of 23.9% [[54]](https://paperpile.com/c/YwsriB/1BBBG).

A study of 1,436 potential donors between March 2010 and August 2015 at Zhongnan Hospital, Wuhan, found that — after 201 became ineligible due to family indecision — 204 (16.5%) agreed to donate while 1,031 (83.5%) refused [[55]](https://paperpile.com/c/YwsriB/yaaO2).

A study of 291 cardiac death potential donors from July 2011 to December 2014 at Jilin University First Affiliated Hospital found that family consented in 152 (52.2%) of cases, with 93 (31.9%) going on to donate [[56]](https://paperpile.com/c/YwsriB/gOeDT).

A study of 778 potential donors between January 2010 and December 2015 at the Guangzhou Military Region General Hospital found a conversion rate of 31% (n=241) [[57]](https://paperpile.com/c/YwsriB/zQAqk). Of the 537 non-donors, 46.4% refused due to “family and social factors,” 25.7% became ineligible for medical reasons, and 27.9% became ineligible due to sub-optimal communication between hospital staff and potential donor families. The 241 cases yielded 234 livers with 26 (11.1%) discarded due to a variety of complications.

A 2015 study by Shandong University states that between June 2012 and June 2015, 104 potential donors were identified at a certain 3A (advanced) hospital. Consent was obtained in only 7 (6.7%) cases, with family members refusing in the other 97 (93.3%) [[58]](https://paperpile.com/c/YwsriB/5ELV0).

A general unwillingness to donate is reflected in the difficulties of the authorities in training and retaining transplant coordinators. Zhang Weiye, director of the transplant center at Tianjin First Central Hospital, said in June 2017 that the hardship and unrewarding nature of the occupation has led transplant coordinators to exit the field at a rate of 15% per year [[59]](https://paperpile.com/c/YwsriB/rvM2s).

The above factors demonstrate the complexity and challenges in China’s efforts to source donors from hospitals. The mathematically precise growth trajectories and transplants/donor ratios reported in central government datasets contrast sharply with the day-to-day realities of locating eligible donors, securing family consent, mobilizing surgical teams, and much more.

## Policy of ‘humanitarian aid’

Official statements about China’s voluntary donation system often make reference to “humanitarian aid.” The policy was outlined by Huang Jiefu in a March 2013 interview as one of the three planks of the voluntary transplantation system [[60]](https://paperpile.com/c/YwsriB/k5OtU). In English-language policy statements, the meaning of this phrase has not been spelled out fully [[61]](https://paperpile.com/c/YwsriB/Zear3). Chinese-language documents, medical papers, and news reports shows that the term refers to coverage of medical expenses and large cash payments to families that consent to the donation of their deceased relative’s organs.

In Zhejiang Province, the Red Cross offered $1600 (about 10500 yuan) for funeral expenses and an allowance of $3200 (about 21000 yuan), plus additional compensation of a maximum of $4800 (about 31000 yuan) “if the families of deceased donors face hardship.”[[62]](https://paperpile.com/c/YwsriB/LVR3s)

In September 2015, Hubei Daily News reported that seven families were paid a total of 510000 yuan in relief, an average of 72857 yuan per family. The Hubei Red Cross reported in September 2015 that standard compensation to families were between 50000 and 90000 yuan [[63]](https://paperpile.com/c/YwsriB/qHOOD). China Daily cites 50000 yuan as standard [[64]](https://paperpile.com/c/YwsriB/irL2x).

These are significant sums relative to the family income of most donors. According to a study of 1235 donors at Zhongnan Hospital in Wuhan, 57% had income less than 500 yuan per month, 30% had income between 500-2000 yuan per month, and 13% had income over 2000 yuan per month [[55]](https://paperpile.com/c/YwsriB/yaaO2).

According to the figures cited in the Zhongnan Hospital study, a sum of 60000 yuan is around 3 times the annual income of 87% of donor families, and at least 10 times the annual income of 57% of donor families. Depending on how much lower than 500 yuan per month the family income is, these ratios may be far greater. The poverty threshold of farmers in China is 2300 yuan annually [[65]](https://paperpile.com/c/YwsriB/k2zuX). If 50000 yuan were paid in financial assistance to a family at the poverty threshold, this would be 21 times annual income. If 90000 yuan were paid to one of these families, it would be nearly 40 times annual income.

China does not publish data on the proportion of families who receive payments, but it appears to be the vast majority. According to a 2012 Tianjin First Central Hospital study, 88% of the consenting families demanded financial compensation [[54]](https://paperpile.com/c/YwsriB/1BBBG). During the pilot program that procured organs from 207 donors from March 2010 to March 2012, most families belonged to the rural poor, and 90% asked for financial aid as a condition of donation [[66]](https://paperpile.com/c/YwsriB/S6kwt).

Given the structure of China’s fee-for-service hospital system, it is justifiable that poor families who elect to donate their deceased relative’s organs be exempt from the health care expenses incurred. The state’s payment for funeral arrangements may also be justified. Large cash payments of many times annual income, however, may constitute coercive payments to obtain consent for donation. Such payments are considered to be financial incentives for consenting to organ donation, and are strictly prohibited by relevant international organizations [[67]](https://paperpile.com/c/YwsriB/R4S3M). Access to data is impeded by a lack of transparency around the policy, the amounts paid, and the criteria for payment. Further research in this area is warranted.

# References

[1. Joint Hearing Before the Subcomm. on Africa, Global Health, Global Human Rights, and International Organizations and the Subcomm. on Europe, Eurasia, and Emerging Threats of the House Comm. on Foreign Affairs, 114th Cong. 2nd Sess. Organ Harvesting: An Examination of a Brutal Practice. 2016.](http://paperpile.com/b/YwsriB/OqiOR) <http://docs.house.gov/meetings/FA/FA16/20160623/105116/HHRG-114-FA16-20160623-SD006.pdf>[.](http://paperpile.com/b/YwsriB/OqiOR)

[2. 机构简介. [Organizational Introduction]. China Organ Transplantation Development Foundation. 2016?](http://paperpile.com/b/YwsriB/ZY89K) <http://www.yzjjh.org/index.php?m=content&c=index&a=lists&catid=13.> [Accessed 1 Oct 2017.](http://paperpile.com/b/YwsriB/ZY89K)

[3. 关于建立人体捐献器官转运绿色通道的通知. [Notice regarding the establishment of an organ donation transport green channel]. National Health and Family Planning Commission; Attachment One. 2016.](http://paperpile.com/b/YwsriB/QAzzm) <http://www.nhfpc.gov.cn/yzygj/s3585/201605/940f44e39f1e452e8e35c37593025537.shtml.> [Accessed 1 Oct 2017.](http://paperpile.com/b/YwsriB/QAzzm)

[4. A Life Changing New Milestone for China’s Organ Transplantation Reforms The Li Ka Shing Foundation Supports Move Towards Transparency | Li Ka Shing Foundation. Li Ka Shing Foundation. 2015.](http://paperpile.com/b/YwsriB/6azvc) <https://www.lksf.org/a-life-changing-new-milestone-for-chinas-organ-transplantation-reforms-the-li-ka-shing-foundation-supports-move-towards-transparency/.> [Accessed 27 Mar 2018.](http://paperpile.com/b/YwsriB/6azvc)

[5. Chen HY. 梵蒂冈教皇科学院院长来华 中国器官捐献与移植制度获国际认可. [Vatican’s Pontifical Academy of Sciences Chancellor‎ comes to China; China’s organ donation and transplantation system receives international acknowledgement]. Caixin. 2017.](http://paperpile.com/b/YwsriB/XkYOj) <http://m.china.caixin.com/m/2017-08-04/101126322.html.> [Accessed 9 Sep 2017.](http://paperpile.com/b/YwsriB/XkYOj)

[6. 黄洁夫: 现任中央保健委员会副主任（正部长级） [Huang Jiefu: Currently serving as vice chairman of the Central Health Care Committee (Ministerial-level)]. Chinese Communist Party Leading Cadre Database.](http://paperpile.com/b/YwsriB/XXncR) <http://archive.is/3F4jv.> [Accessed 5 Oct 2017.](http://paperpile.com/b/YwsriB/XXncR)

[7. 沈中阳同志担任中国人体器官捐献管理中心主任. [Comrade Shen Zhongyang is made director of the China Organ Donation Administration Center]. China Organ Donation Administration Center. 2013.](http://paperpile.com/b/YwsriB/UcpqU) <https://www.codac.org.cn/news/dynamics/20170601/694350.htm.> [Accessed 25 May 2017.](http://paperpile.com/b/YwsriB/UcpqU)

[8. Delmonico F, Chapman J, Fung J, Danovitch G, Levin A, Capron A, et al. Open letter to Xi Jinping, President of the People’s Republic of China: China's fight against corruption in organ transplantation. Transplantation. 2014;97:795–6.](http://paperpile.com/b/YwsriB/PABN9)

[9. Huang J, Millis JM, Mao Y, Millis MA, Sang X, Zhong S. Voluntary organ donation system adapted to Chinese cultural values and social reality. Liver Transpl. 2015;21:419–22.](http://paperpile.com/b/YwsriB/KUYYl)

[10. Fung J. The sleeping giant awakens-liver transplantation in China. Am J Transplant. 2010;10:1723–4.](http://paperpile.com/b/YwsriB/0qKMr)

[11. Center P. 中国肝移植注册中心落户浙大一院. [The China Liver Transplant Registry arrives at Zhejiang First Affiliated]. Zhejiang First Affiliated Hospital. 2014.](http://paperpile.com/b/YwsriB/4HIc0) <http://www.zy91.com/zxxw/2136.jhtml.> [Accessed 1 Dec 2017.](http://paperpile.com/b/YwsriB/4HIc0)

[12. Xu G. 原卫生部副部长：周永康落马打破死囚器官移植利益链 [Former Health Minister: Zhou Yongkang’s downfall broke the profit chain using death row prisoner organs]. Phoenix (via Caijing). 2015.](http://paperpile.com/b/YwsriB/mIsG) [http://politics.caijing.com.cn/20150316/3840670.shtml; https://web.archive.org/web/20180611154503/http://politics.caijing.com.cn/20150316/3840670.shtml.](http://politics.caijing.com.cn/20150316/3840670.shtml;%20https://web.archive.org/web/20180611154503/http://politics.caijing.com.cn/20150316/3840670.shtml.) [Accessed 1 Dec 2018.](http://paperpile.com/b/YwsriB/mIsG)

[13. Chang C. 留下最后的“生命礼物”. [Leave behind the final “gift of life”]. People’s Daily. 2015.](http://paperpile.com/b/YwsriB/7qlSX) <http://www.bjwmb.gov.cn/zxgc/wmsj/t20150410_641507.htm.> [Accessed 29 Jul 2017.](http://paperpile.com/b/YwsriB/7qlSX)

[14. Xu X. 我省首例DCD捐献完成. [First DCD donation carried out in the province]. Heilongjiang Daily. Heilongjiang Daily. 2015.](http://paperpile.com/b/YwsriB/biBYJ) <http://news.xinhuanet.com/local/2015-06/05/c_127881972.htm.> [Accessed 6 Oct 2017.](http://paperpile.com/b/YwsriB/biBYJ)

[15. Department of Health & Human Services (DHHS) Centers for Medicare & Medicaid Services (CMS). CMS Manual System. 2014.](http://paperpile.com/b/YwsriB/ubGG1) <https://www.cms.gov/Regulations-and-Guidance/Guidance/Transmittals/downloads/R115SOMA.pdf>[.](http://paperpile.com/b/YwsriB/ubGG1)

[16. Liu YQ. “只要对推广器官捐献有帮助，登记平台越多越好”. [As long as it’s helpful to organ donation, the more who register on the platform the better]. China Economic Weekly. 2014.](http://paperpile.com/b/YwsriB/dQ4gG) <http://paper.people.com.cn/zgjjzk/html/2014-04/21/content_1418864.htm.> [Accessed 6 Oct 2017.](http://paperpile.com/b/YwsriB/dQ4gG)

[17. Yang J. 自愿捐献器官有多难. [The great difficulties of voluntary organ donation]. Xinmin Weekly. 2015.](http://paperpile.com/b/YwsriB/JaHOw) <http://www.xinminweekly.com.cn/News/Content/5445.> [Accessed 6 Oct 2017.](http://paperpile.com/b/YwsriB/JaHOw)

[18. 黄洁夫：死囚可否捐器官是伪命题 [Huang Jiefu: Whether prisoners can donate organs or not is a false question]. Beijing Youth Daily. 2015.](http://paperpile.com/b/YwsriB/8damK) <http://epaper.ynet.com/html/2015-11/23/content_167300.htm?div=1>[.](http://paperpile.com/b/YwsriB/8damK)

[19. 国家卫生计生委中国红十字会总会关于成立中国人体器官捐献与移植委员会的通知. [Notice by the National Health and Family Planning Commission and the Red Cross Society of China on the Establishment of the National Organ Donation and Transplantation Committee]. National Health and Family Planning Commission, Red Cross Society of China. 2014.](http://paperpile.com/b/YwsriB/9MdBH) <http://www.nhfpc.gov.cn/yzygj/s3585u/201403/7f8a8f02b890417281c75f6f83aeb215.shtml.> [Accessed 2 Dec 2018.](http://paperpile.com/b/YwsriB/9MdBH)

[20. Chen JY. 肺移植供体肺的维护及获取. [Preservation and procurement of transplant lungs from lung donors]. Medical Journal of Wuhan University. 37:540–2.](http://paperpile.com/b/YwsriB/ROZWL)

[21. Han W, Zhang HB. 奔走人生，玉汝于成——安贞医院二十二年心脏移植的历程. [Rushing through life, so a jade is formed -- The story of 22 years of heart transplants at Anzhen Hospital]. Anzhen Hospital, Heart Surgery Unit. 2015.](http://paperpile.com/b/YwsriB/HQ4Hs) <http://www.anzhen.org/Hospitals/Journals/ArticleIndex/890.> [Accessed 3 Dec 2018.](http://paperpile.com/b/YwsriB/HQ4Hs)

[22. Long YQ, Wang DB. 深度访谈：器官捐献无偿 为什么移植很贵. [In-depth interview: Organs are donated without compensation, so why is transplantation so expensive]. Southern Metropolis Daily. 2017.](http://paperpile.com/b/YwsriB/zgNPt) <http://gd.qq.com/a/20150306/007526.htm.> [Accessed 6 Mar 2015.](http://paperpile.com/b/YwsriB/zgNPt)

[23. Zhao WJ, Zhu S, Ma B, Zhao LP, Zhao WZ. 重症监护病房12例脑死亡器官捐献供体的维护及护理. [Maintenance and nursing of 12 brain death organ donation donor in ICU]. Prenat Neonatal Med. 19:62–4.](http://paperpile.com/b/YwsriB/zHmp2)

[24. Party Committee Office. 心外科党支部全体党员在呈贡医院进行第二例原位心脏移植术过程中吃苦在前. [The hardships of all Party members of the Party cell in the heart surgery unit during Kunming Hospital’s second orthotopic cardiac transplantation surgery]. First Affiliated Hospital of Kunming Medical University. 2016.](http://paperpile.com/b/YwsriB/xJ5jU) <http://www.ydyy.cn/Subject/DJGZ_WXJS/Article/5282.htm.> [Accessed 4 Dec 2017.](http://paperpile.com/b/YwsriB/xJ5jU)

[25. 大事记:慈溪实现第7例器官捐献. [Annal of Major Events: Cixi completes 7th organ donation]. Guangdong Branch Red Cross Society of China. 2017.](http://paperpile.com/b/YwsriB/Pc5I3) <http://www.gdredcross.org.cn/gyhsz/dsj/175.html.> [Accessed 9 Dec 2016.](http://paperpile.com/b/YwsriB/Pc5I3)

[26. Li WC, Zhao JW, Luan YX, Fu SL, He C, Pan J, et al. 吉林省颅脑损伤后器官捐献140例临床分析. [Clinical analysis of 140 cases of organ donation after brain injury in Jilin province]. Chin J Neurotrauma Surg (Electronic Edition). 2016;2:74–7.](http://paperpile.com/b/YwsriB/efwSH)

[27. 贵州女子福建南安遇车祸成植物人 捐献器官将救活三人. [Guizhou woman in car accident in Nan’an, Fuzhou, enters vegetative state, donates organs to save three people]. Quanzhou News. 2016.](http://paperpile.com/b/YwsriB/tEHOi) <https://read01.com/8k0ejG.html.> [Accessed 6 Oct 2017.](http://paperpile.com/b/YwsriB/tEHOi)

[28. 福建：器官捐献终突破100例 [Fujian: Organ donations finally break through 100 cases]. 2016.](http://paperpile.com/b/YwsriB/epMo7) <http://www.duxuan.cn/doc/21382350.html>[.](http://paperpile.com/b/YwsriB/epMo7)

[29. Wei ZH, Pan F, Jiang Y. 脂肪变性供肝对肝移植手术安全性的临床研究. [For hepatic steatosis influence of clinical research on the safety of a liver transplant]. Journal of Hepatobiliary Surgery. 2014;22:452–5.](http://paperpile.com/b/YwsriB/DkwAJ)

[30. Yu Z, Sun Z, Yu S, Zhang L, Feng X, Xu X, et al. Safety limitations of fatty liver transplantation can be extended to 40%: Experience of a single centre in China. Liver Int. 2016.](http://paperpile.com/b/YwsriB/5Bt3V) <http://simsrad.net.ocs.mq.edu.au/login?url=http://ovidsp.ovid.com/ovidweb.cgi?T=JS&CSC=Y&NEWS=N&PAGE=fulltext&D=emed18b&AN=613492928>[.](http://paperpile.com/b/YwsriB/5Bt3V)

[31. Rogers WA, Fiatarone Singh MA, Lavee J. Papers based on data concerning organs from executed prisoners should not be published. Liver Int. 2017;37:769.](http://paperpile.com/b/YwsriB/j6Is3)

[32. Publisher’s Note. Liver Int. 2017;37:770–770.](http://paperpile.com/b/YwsriB/nIoos)

[33. Rogers WA, Fiatarone Singh MA, Lavee J. Papers based on data concerning organs from executed prisoners should not be published: Response to Zheng and Yan. Liver Int. 2017;37:771–2.](http://paperpile.com/b/YwsriB/2Wuxc)

[34. Mondelli M, Younossi Z, Negro F. Editor’s Note. Liver Int. 2017;37:768.](http://paperpile.com/b/YwsriB/VOhBZ)

[35. Wang YY. 黄洁夫谈院士因器官来源被撤稿: 实事求是，依法推进移植事业. [Huang Jiefu discusses the retraction due to organ sourcing: seek truth from facts, promote the enterprise of transplantation in accordance with the law]. The Paper. 2017.](http://paperpile.com/b/YwsriB/MT1cT) <https://archive.is/QKQxf.> [Accessed 7 Oct 2017.](http://paperpile.com/b/YwsriB/MT1cT)

[36. Hospital Introduction. The First Affiliated Hospital, Zhejiang University. 2016.](http://paperpile.com/b/YwsriB/krOPM) <https://archive.is/b7WIs.> [Accessed 5 Oct 2017.](http://paperpile.com/b/YwsriB/krOPM)

[37. Chinese Journal of Transplantation. Chinese Medical Association.](http://paperpile.com/b/YwsriB/b3jGw) <http://www.zhyzzz.org/.> [Accessed 5 Oct 2017.](http://paperpile.com/b/YwsriB/b3jGw)

[38. 2017 Chinese Transplant Congress & 4[th] Chinese Transplant Doctors Congress. Medcon Conference Management System. 2017.](http://paperpile.com/b/YwsriB/TTkuX) <http://www.medmeeting.org/Home/Program/5083.> [Accessed 5 Oct 2017.](http://paperpile.com/b/YwsriB/TTkuX)

[39. Wang HB, Jiang WS, Zhou ZY, Long JY, Li W, Sheung TF. Liver transplantation in mainland China: the overview of CLTR 2011 annual scientific report. Hepatobiliary Surg Nutr. 2013;2:188–97.](http://paperpile.com/b/YwsriB/XnzqO)

[40. Xu X. 浙大一院肝移植团队闪耀 “2015国际肝移植协会中国行” [Zhejiang First Affiliated Hospital’s liver transplant team radiates, “2015 international liver transplant conference is held in China”]. Good Doctor Online. 2015.](http://paperpile.com/b/YwsriB/lOPqA) [http://www.haodf.com/zhuanjiaguandian/zyxuxiao_3890910923.htm; https://archive.is/ROnto.](http://www.haodf.com/zhuanjiaguandian/zyxuxiao_3890910923.htm;%20https://archive.is/ROnto.) [Accessed 5 Oct 2017.](http://paperpile.com/b/YwsriB/lOPqA)

[41. 肺移植：37年来不能忘却的纪念. [Lung Transplantation: Memories from 37 years that will never be forgotten]. Headline News. 2016.](http://paperpile.com/b/YwsriB/AOXmq) <http://www.toutiao.com/i6241310365456531970/.> [Accessed 3 Oct 2017.](http://paperpile.com/b/YwsriB/AOXmq)

[42. 无锡市人医与北京中日医院强强联手成立医联体. Wuxi Municipal People’s Hospital establishes strong ties with China-Japan Friendship Hospital in Beijing. Xinhua. 2017.](http://paperpile.com/b/YwsriB/Gw9JW) <https://archive.is/y8hPu.> [Accessed 5 Oct 2017.](http://paperpile.com/b/YwsriB/Gw9JW)

[43. Yang MJ. 无锡4年仅实现10例器官捐献. [Wuxi has only performed 10 organ donations in 4 years]. Wuxi Daily. 2014.](http://paperpile.com/b/YwsriB/V2snh) <https://archive.is/YwMkw.> [Accessed 1 Oct 2017.](http://paperpile.com/b/YwsriB/V2snh)

[44. 桂林患者捐双肺“飞”来无锡救人. [Two lungs from Guilin patient “fly” to Wuxi to save people]. Jiangnan Wanbao. 2015.](http://paperpile.com/b/YwsriB/2ZLNS) <http://www.organdonation.org.cn/ZJiangSuDiQu/2015-03/6458.htm.> [Accessed 3 Oct 2017.](http://paperpile.com/b/YwsriB/2ZLNS)

[45. Yang MJ. 无锡5年多成功完成29例器官捐献. [Wuxi successfully performed 29 organ donations in 5 years]. Wuxi Daily. 2017.](http://paperpile.com/b/YwsriB/QKVKf) <https://archive.is/Rqu2O.> [Accessed 28 Sep 2017.](http://paperpile.com/b/YwsriB/QKVKf)

[46. Tatlow DK. Organ Transplants Suffer Amid China’s Transportation Delays. The New York Times. 2015.](http://paperpile.com/b/YwsriB/YYzYc)

[47. Jiang HC, Chen Y, Chen JY. 心脏死亡供肺肺移植临床分析 [Clinical Analysis of Lung Transplantation from Donor of Cardiac Death]. J Nanjing Norm Univ. 2017;40:92–7.](http://paperpile.com/b/YwsriB/3u6uM)

[48. Wang P. 青岛大学附属医院成立器官移植中心. [Qingdao University Affiliated Hospital established organ transplant center]. Qingdao News Online. 2014.](http://paperpile.com/b/YwsriB/l2Y8y) <http://news.eastday.com/eastday/13news/auto/news/society/u7ai828453_K4.html.> [Accessed 4 Oct 2017.](http://paperpile.com/b/YwsriB/l2Y8y)

[49. Propaganda office. 攀登永不止步打造国内一流的器官移植中心. [Forging ahead will never cease. Create the best organ transplant center in China]. Qingdao University Affiliated Hospital. 2014.](http://paperpile.com/b/YwsriB/foybi) <http://qdumh.qd.sd.cn/a/yiyuandongtai/yiyuanxinwen/2014/0805/3287.html.> [Accessed 9 Oct 2017.](http://paperpile.com/b/YwsriB/foybi)

[50. 器官移植中心成立两周年. [Anniversary of the Second Year of the Founding of the Organ Transplant Center]. Qingdao, Shandong (China): Qingdao University Affiliated Hospital. 2016.](http://paperpile.com/b/YwsriB/7LgDL) <http://qdumh.qd.sd.cn/index.php/yiyuanxinwen/8-yiyuanxinwen/3271-2016-03-02-12-49-39.> [Accessed 5 Oct 2017.](http://paperpile.com/b/YwsriB/7LgDL)

[51. 2015年的最后奇迹 浙医二院移植团队12小时完成8台移植手术. [The final miracle of 2015. Zhejiang Second Affiliated Hospital transplant team performs 8 transplants within 12 hours]. Beijing: China Organ Transplantation Development Foundation. 2016.](http://paperpile.com/b/YwsriB/rCv9q) <http://savelife.org.cn/outer/index4.html?newsId=155.> [Accessed 10 Oct 2017.](http://paperpile.com/b/YwsriB/rCv9q)

[52. Zhang XJ, Wang R. 创纪录！浙大一院48小时完成14台大器官移植. [A record is set! Zhejiang University First Affiliated Hospital performs 14 organ transplants within 48 hours]. Zhejiang News Client. 2017.](http://paperpile.com/b/YwsriB/KGyIp) <http://zj.zjol.com.cn/news/589142.html.> [Accessed 9 Oct 2017.](http://paperpile.com/b/YwsriB/KGyIp)

[53. Chen ZQ. 原卫生部副部长黄洁夫：有器官移植资质医院将“洗牌”. [Former vice minister of health Huang Jiefu: Organ transplant-authorized hospitals to be “reshuffled”]. The Paper. 2017.](http://paperpile.com/b/YwsriB/bhfp6) <http://www.thepaper.cn/newsDetail_forward_1632010.> [Accessed 2 Sep 2017.](http://paperpile.com/b/YwsriB/bhfp6)

[54. Zhong Y, Zhao Y, Zhang WY, Cai JZ, Deng YL, Shen ZY. DCD器官捐献者家属访谈心理因素调查分析 [Interview-based investigation and analysis of psychological factors in DCD organ donor families]. Journal of Nurses Training. 2012;27:1657–9.](http://paperpile.com/b/YwsriB/1BBBG)

[55. Si J, Huang W, Gu Y, Yan BB, Xu Z, Li ZJ, Zheng Z, Ye QF. 中国公民逝世后器官捐献者家属捐献意愿影响因素分析. [A study of factors influencing family decision of Chinese donation after citizen’s death]. Chin J Transplant (Electronic Edition). 2015;9:174–7.](http://paperpile.com/b/YwsriB/yaaO2)

[56. Pan J, Wang HF, Zhang DH, Li WC, Chen B, He X, et al. 公民逝世后潜在供者器官捐献效率的影响因素分析 [Analysis of Influencing Factors of Donation Efficiency Among Cardiac Death Potential Donors]. Chinese Hospital Management. Aug 2015;35:62–4.](http://paperpile.com/b/YwsriB/gOeDT)

[57. Zhao JQ, Huo F, Li P, Wang SP, Zheng YJ, Li H. 中国心脏死亡器官捐献工作发展及影响因素:单中心经验. [Development and influence factors of China donation after cardiac death: single-center experience]. Chin J Transplant (Electronic Edition). 2017;11:32–6.](http://paperpile.com/b/YwsriB/zQAqk)

[58. Su Q. 如果生命马上结束，你愿意用器官帮助别人吗？[If you were about to die, would you donate your organs to help others?]. Caijing. 2016.](http://paperpile.com/b/YwsriB/5ELV0) [http://yuanchuang.caijing.com.cn/2016/1210/4209909.shtml; http://archive.is/ZDb8c.](http://yuanchuang.caijing.com.cn/2016/1210/4209909.shtml;%20http://archive.is/ZDb8c.) [Accessed 8 Oct 2017.](http://paperpile.com/b/YwsriB/5ELV0)

[59. Sun GX, ed. 传递生生不息的力量——天津市第一中心医院成功举办全国第二期器官捐献协调员强化. [Pass on the energy—Tianjin First Central Hospital successfully holds second national training class for strengthening transplant coordinators]. Tianjin (China): Tianjin First Central Hospital. 2017.](http://paperpile.com/b/YwsriB/rvM2s) <http://www.tj-fch.com/sitecn/yyxw/1596_10550.html.> [Accessed 12 Oct 2017.](http://paperpile.com/b/YwsriB/rvM2s)

[60. 卫生部原副部长黄洁夫:我要带头向捐献者鞠躬. [Former vice minister of health Huang Jiefu: I want to be the first to bow before organ donors]. Guangzhou Daily. 2017.](http://paperpile.com/b/YwsriB/k5OtU) <http://news.21cn.com/caiji/roll/a/2013/0313/09/14785992.shtml.> [Accessed 13 Mar 2013.](http://paperpile.com/b/YwsriB/k5OtU)

[61. Huang J. The “Chinese Mode” of organ donation and transplantation. Hepatobiliary Surg Nutr. 2017;6:212–4.](http://paperpile.com/b/YwsriB/Zear3)

[62. Wu X, Fang Q. Financial compensation for deceased organ donation in China. J Med Ethics. 2013;39:378–9.](http://paperpile.com/b/YwsriB/LVR3s)

[63. 湖北红会拨付人体器官捐献救助金. [Hubei Red Cross appropriates organ donation humanitarian aid funds]. Red Cross Society of China Press. 2015.](http://paperpile.com/b/YwsriB/qHOOD) <http://news.redcrossol.com/miropaper/article.aspx?aid=3209&ty=zghszbkzk.> [Accessed 12 Oct 2017.](http://paperpile.com/b/YwsriB/qHOOD)

[64. Wang QY. Organ donors’ families “need financial help.” China Daily. 2012.](http://paperpile.com/b/YwsriB/irL2x) <http://www.chinadaily.com.cn/china/2012-09/22/content_15775024.htm.> [Accessed 5 Jul 2017.](http://paperpile.com/b/YwsriB/irL2x)

[65. Mengjie, ed. Facts & Figures: China’s fight against poverty. Xinhua. 2017.](http://paperpile.com/b/YwsriB/k2zuX) <http://news.xinhuanet.com/english/2017-05/24/c_136311122.htm.> [Accessed 1 Oct 2017.](http://paperpile.com/b/YwsriB/k2zuX)

[66. MacLeod C. Organ harvesting changes in China will be tough to realize. USA Today. 2012.](http://paperpile.com/b/YwsriB/S6kwt) <http://www.usatoday.com/news/world/story/2012-05-14/china-organ-transplant-ban/54964098/1.> [Accessed 29 Mar 2018.](http://paperpile.com/b/YwsriB/S6kwt)

[67. Capron AM, Delmonico FL, Dominguez-Gil B, Martin DE, Danovitch GM, Chapman J. Statement of the Declaration of Istanbul Custodian Group Regarding Payments to Families of Deceased Organ Donors. Transplantation. 2016;100:2006.](http://paperpile.com/b/YwsriB/R4S3M)
